# Calmodulin‐like protein CML15 interacts with PP2C46/65 to regulate papaya fruit ripening via integrating calcium, ABA and ethylene signals

**DOI:** 10.1111/pbi.14297

**Published:** 2024-02-06

**Authors:** Qiunan Zhu, Qinqin Tan, Qiyang Gao, Senlin Zheng, Weixin Chen, Jean‐Philippe Galaud, Xueping Li, Xiaoyang Zhu

**Affiliations:** ^1^ Guangdong Provincial Key Laboratory of Postharvest Science of Fruits and Vegetables/Engineering Research Center for Postharvest Technology of Horticultural Crops in South China, Ministry of Education, College of Horticulture South China Agricultural University Guangzhou China; ^2^ Laboratoire de Recherche en Sciences Végétales Université de Toulouse, CNRS, UPS Castanet‐Tolosan France

**Keywords:** calcium signal, calmodulin‐like protein, ABA signal, protein interaction, signal cross‐talk, fruit ripening

## Abstract

It is well known that calcium, ethylene and abscisic acid (ABA) can regulate fruit ripening, however, their interaction in the regulation of fruit ripening has not yet been fully clarified. The present study found that the expression of the papaya calcium sensor *CpCML15* was strongly linked to fruit ripening. CpCML15 could bind Ca^2+^ and served as a true calcium sensor. CpCML15 interacted with CpPP2C46 and CpPP2C65, the candidate components of the ABA signalling pathways. *CpPP2C46/65* expression was also related to fruit ripening and regulated by ethylene. CpCML15 was located in the nucleus and CpPP2C46/65 were located in both the nucleus and membrane. The interaction between CpCML15 and CpPP2C46/65 was calcium dependent and further repressed the activity of CpPP2C46/65 *in vitro*. The transient overexpression of *CpCML15* and *CpPP2C46/65* in papaya promoted fruit ripening and gene expression related to ripening. The reduced expression of *CpCML15* and *CpPP2C46/65* by virus‐induced gene silencing delayed fruit colouring and softening and repressed the expression of genes related to ethylene signalling and softening. Moreover, ectopic overexpression of *CpCML15* in tomato fruit also promoted fruit softening and ripening by increasing ethylene production and enhancing gene expression related to ripening. Additionally, CpPP2C46 interacted with CpABI5, and CpPP2C65 interacted with CpERF003‐like, two transcriptional factors in ABA and ethylene signalling pathways that are closely related to fruit ripening. Taken together, our results showed that *CpCML15* and *CpPP2Cs* positively regulated fruit ripening, and their interaction integrated the cross‐talk of calcium, ABA and ethylene signals in fruit ripening through the CpCML15‐CpPP2Cs‐CpABI5/CpERF003‐like pathway.

## Introduction

The calcium ion (Ca^2+^) is a crucial regulator in various horticultural crops as a multifunctional signalling ion. Indeed, calcium can maintain fruit firmness and regulate fruit quality and ripening (Aghdam *et al*., 2012; Gao *et al*., 2019, 2020; Wei and Zhao, 2020). As a safe and nontoxic preservative, calcium application can effectively delay ripening and senescence and maintain the quality of fruits and vegetables, thereby effectively prolonging their shelf life (Aghdam *et al*., 2012; Gao *et al*., 2020; Madani *et al*., 2014; Xu *et al*., 2022; Zhao and Wang, 2015). In papaya, pre‐ or post‐harvest calcium applications can also efficiently delay fruit ripening and senescence, reduce the occurrence of post‐harvest diseases, and maintain fruit quality in papaya (Gao *et al*., 2020; Madani *et al*., 2014, 2016). However, the underlying molecular mechanism of calcium in fruit ripening regulation remains unclear.

Calcium signals are sensed by calcium sensors and translated, amplified, and transduced to downstream targets to elicit cellular responses. In plants, there are three main kinds of calcium‐sensor proteins: calmodulin proteins (CaMs) and calmodulin‐like proteins (CMLs), calcium‐dependent protein kinases (CDPKs) and calcineurin B‐like proteins (CBLs) (Luan *et al*., 2002). Several studies have reported that calcium‐sensor proteins are involved in fruit ripening or senescence (Ding *et al*., 2018; Gao *et al*., 2019). For example, CDPK‐related kinase LeCRK1 expression increases at the late stage of the fruit ripening period and was induced by ethylene, which may have participated in the ripening of tomato (Leclercq, 2004). ACC synthase (ACS) works as a critical rate‐limiting enzyme for ethylene synthesis and CDPK participates in ethylene signalling by phosphorylating ACS to affect fruit ripening (Gallardo *et al*., 1999). LeACS2 can be phosphorylated by the calcium sensor CDPK, and its phosphorylation site is conserved in the ACS family in tomato (Sebastià *et al*., 2004). In apple fruit, MdCDPK7 phosphorylates 1‐AMINOCYCLOPROPANE‐1‐CARBOXYLIC ACID OXIDASE1 (MdACO1), resulting in the degradation of MdACO1 and reduction of ethylene, involved in Ca^2+^ delayed fruit ripening (Xu *et al*., 2023). CaCl_2_ treatment can delay fruit ripening and repress ethylene production. A recent study in apple reveals that a key player, MdCYTOKININ RESPONSE FACTOR4 (MdCRF4), can positively regulate the expression of MdACS1 and MdERF3, which further regulate ethylene biosynthesis and fruit ripening. More importantly, CaCl_2_ application can promote the Ca^2+^/CaM‐mediated phosphorylation of MdCRF4, involving its ubiquitination and degradation, thus suppressing ethylene production and delaying fruit ripening (Li *et al*., 2023). These findings indicates that calcium‐sensor proteins play critical roles in regulating fruit ripening and senescence.

CaMs are a core component of the calcium signalling network and the most well‐studied calcium sensors in plants. CaMs participate in various cellular life activities by interacting with target proteins (Yang and Poovaiah, 2002). When binding to calcium, CaMs undergo structural changes and bind to target proteins, allowing signal transduction to downstream proteins, and triggering specific physiological responses (DeFalco *et al*., 2010). CaMs are related to fruit ripening. For example, *SlCaM2* is highly expressed and strongly induced by exogenous ethylene during tomato fruit ripening. Transient *SlCaM2* overexpression can delay fruit ripening, while the inhibition of its expression accelerates fruit ripening in tomato (Yang *et al*., 2014). Calmodulin‐like proteins (CMLs) are calcium sensors that belong to the same family as CaMs, but unlike CaMs, much of their function remain unknown (DeFalco *et al*., 2010). An increasing number of CML family genes have been cloned and identified in horticultural crops including strawberry, grape, tomato and apple, and their regulatory roles in fruit growth, fruit development, ripening and stress responses have been revealed (Tang *et al*., 2021; Vandelle *et al*., 2018; Zhang *et al*., 2016, 2017). Recently, it has been reported that EjCML19 in loquat interacts with EjWRKY7 to synergistically regulate the expression of browning‐related genes, thus participating in the regulation of CaCl_2_‐alleviated chilling injury (Hou *et al*., 2023). Nevertheless, the function of CMLs in fruit ripening and senescence has rarely been reported and only a few has been reported as expressed during fruit ripening and regulated by ethylene (Munir *et al*., 2016) and senescence (Tang *et al*., 2020). It has been demonstrated that apple MdCML15 is involved in the wound‐triggered leaf senescence, and transiently overexpressing *MdCML15* in tobacco and apple leaves accelerate leaf senescence. In addition, MdCML15 interactes with MdVQ10 and further enhances the interaction between MdVQ10 and MdWRKY75, for the regulation of senescence‐related genes (Zhang *et al*., 2023). In papaya, the *CpCML15* gene expression is closely related to fruit ripening, which is strictly regulated by ethylene and may participate in fruit ripening (Ding *et al*., 2018). These results demonstrate that CaM/CMLs might play critical roles in fruit ripening and senescence, but the possible mechanism is still unclear.

Both ethylene and Abscisic acid (ABA) are known to play crucial roles in fruit ripening and senescence (Fenn and Giovannoni, 2021). Ethylene is the most studied hormone in fruit ripening, but ABA also regulates ripening and senescence process by regulating ABA‐related transcription factors in an ethylene‐dependent or ‐independent manner (Bai *et al*., 2021; Jia *et al*., 2016; Leng *et al*., 2013). In non‐climacteric fruits and vegetables, such as citrus, grapes, strawberries and cucumbers, ABA regulates fruit development, coloration, softening and quality formation and functions critically in regulating fruit ripening (Bai *et al*., 2021; Setha, 2012; Shen and Rose, 2014). Therefore, ABA is a crucial trigger signal for fruit development and a key hormone regulating fruit development and senescence of non‐climacteric and climacteric fruits (Kou *et al*., 2021; Sun *et al*., 2012).

Type 2C protein phosphatases (PP2Cs) are a major class of protein phosphatase enzymes that dephosphorylates Ser/Thr residues, which are involved in stress signalling, ABA signalling, plant immunity, plant development and nutrient signalling (Qiu *et al*., 2022). The PP2Cs group A is a key regulator of the ABA signalling pathway (Ma *et al*., 2009; Wang *et al*., 2021). PP2Cs interact with SNF1‐related protein kinase 2 (SnRK2) to repress SnRK2 phosphokinase activity in the absence of ABA, thereby repressing ABA signalling. The binding of ABA molecules to the receptors pyrabactin resistant (PYR), pyrabactin resistant‐like (PYL) and regulatory component of the ABA receptor (RCAR) leads to structural modification of the receptors so that the ABA receptors interact with PP2Cs, thereby breaking the interaction between SnRK2 and PP2Cs. Furthermore, PP2Cs activate downstream targets, initiate ABA signalling responses, and are involved in various plant development and stress adaption processes (Klingler *et al*., 2010). The roles of clade A PP2Cs are extensively studied. In *Arabidopsis*, the clade A PP2Cs such as PP2CA, ABA‐insensitive 1 (ABI1), ABI2 and hypersensitive to ABA 1 (HAB1), negatively regulate ABA signalling (Singh *et al*., 2015). The clade B of PP2Cs act as the mitogen‐activated protein kinase (MAPK) phosphatases. For example, the AtAP2C1 interacts with MPK4 and MPK6 and participates in defence and phytohormone responses (Schweighofer *et al*., 2007). It has been recently revealed that the clade I protein phosphatase 2C, TaPP2C158 is also a negative regulator in ABA signal pathway and negatively related to wheat drought tolerance through TaSnRK1.1‐TaAREB3 pathway (Wang *et al*., 2023). Another study reports that the clade D‐member of the PP2C protein, SAL1, positively regulates aluminium resistance in a ABA‐independent way (Xie *et al*., 2023). These findings indicate that PP2Cs have diverse roles in plants, but their specific functions still need to be deciphered.

Although calcium and ABA play critical functions in fruit ripening and plant stress responses, few studies have reported their interaction in the regulation of fruit ripening (Song *et al*., 2018). Our previous work identified a group of ripening‐related CpCMLs in papaya fruit, and *CpCML15* showed the highest expression level among all other *CpCMLs/CpCaMs* in fruit, and strictly regulated by ethylene. The present study further confirmed that CpCML15 was involved in fruit ripening and it interacted with CpPP2C46/65, the candidate elements of ABA signalling, whose expression was also closely co‐related to fruit ripening. The roles of CpCML15 and CpPP2C46/65 in fruit ripening were verified by ectopic or transient overexpression and virus‐induced gene silencing (VIGS) in tomato and papaya fruit. The target proteins of CpPP2C46/65 were also identified. The possible molecular mechanism of the interaction of calcium and ABA and ethylene signals in regulating fruit ripening was investigated. The results provide a theoretical research foundation for the regulation of postharvest fruit ripening in papaya fruit.

## Results

### *CpCML15* expression was closely related to fruit ripening

**Figure 1:**
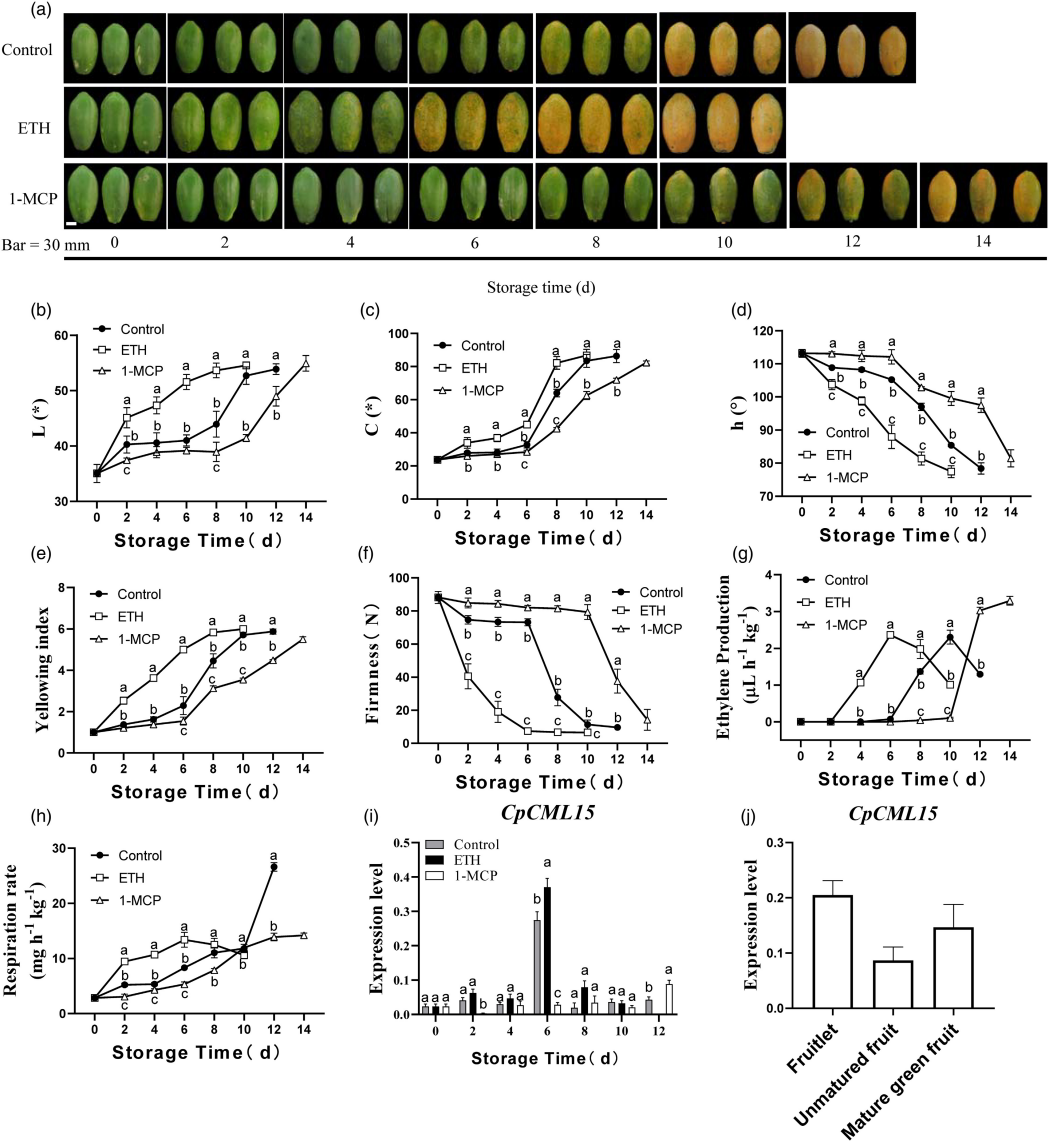
Manipulation of papaya ripening by ethephon (ETH) and 1‐MCP treatments and expression profiles of *CpCML15* under different conditions. (a) Images of papaya fruit during storage after ETH/1‐MCP treatments. (b–h) Effects of ETH/1‐MCP treatments on colour index included L value (b), C value (c) and h value (d), yellowing index (e), pulp firmness (f), ethylene production (g) and respiration rate (h) in papaya fruit. (i, j) The expression of *CpCML15* under ethephon/1‐MCP treatments during storage (i) and during fruit development (j). The fruitlet samples were collected about 30 days after anthesis. The unmatured fruit were collected about 90 days after anthesis. The mature green fruit were collected about 120 days after anthesis, when fruit showed slight yellow around the blossom end/fruit top. The gene expression analysis was determined by RT–qPCR. The *CpTBP1* and *CpTBP2* were used as reference genes. The gene expression level of *CpCML15* was related to the reference genes (geometric mean of *CpTBP1* and *CpTBP2*), which calculated by $2^{-\Delta C_{t}}$ formula. Data are presented as the means ± SD of three biological replicates. The different letters indicate statistical differences between treatments at the 5% level.

Our previous work identified several CMLs in papaya, and gene expression analysis revealed of a group of CMLs closely related to fruit ripening, including *CpCML15*. We further investigated the expression profiles of *CpCML15* under different conditions. As shown in Figure 1, ethephon treatment accelerated fruit ripening including fruit colouring, softening, respiration rate and ethylene production as compared with control group (Figure 1a–h). However, 1‐methylcyclopropene (1‐MCP, an ethylene action inhibitor) treatment delayed ripening by repressing these processes. *CpCML15* showed relative stable expression levels during the early storage, but increased dramatically when fruit initially ripen, with a peak observed on the 6^th^ day of storage and then decreased in the control condition (Figure 1i). Ethephon treatment induced the expression of *CpCML15*, which showed the highest expression on the 6^th^ day. However, 1‐MCP severely suppressed *CpCML15* expression (Figure 1i). The expression of *CpCML15* decreased slightly during the development, and then increased during the maturation stage (Figure 1j). The expression profile of *SlCML15*, the homologous gene of *CpCML15* in tomato, was obtained from Tomexpress and also characterized, which gradually increased with fruit development and ripening, both in fruit flesh and peel. A higher expression level was observed in the fruit peel than in the pulp (Figure S1a). The expression of *SlCML15* was also induced by 1‐aminocyclopropane‐1‐carboxylic acid (ACC) treatment, indicating that *SlCML15* may also be involved in the fruit ripening (Figure S1b). These results provided a basis for the functional validation of *CpCML15* in tomato fruit.

### *CpCML15* encoded a CaM‐like Ca^2+^‐binding protein

**Figure 2:**
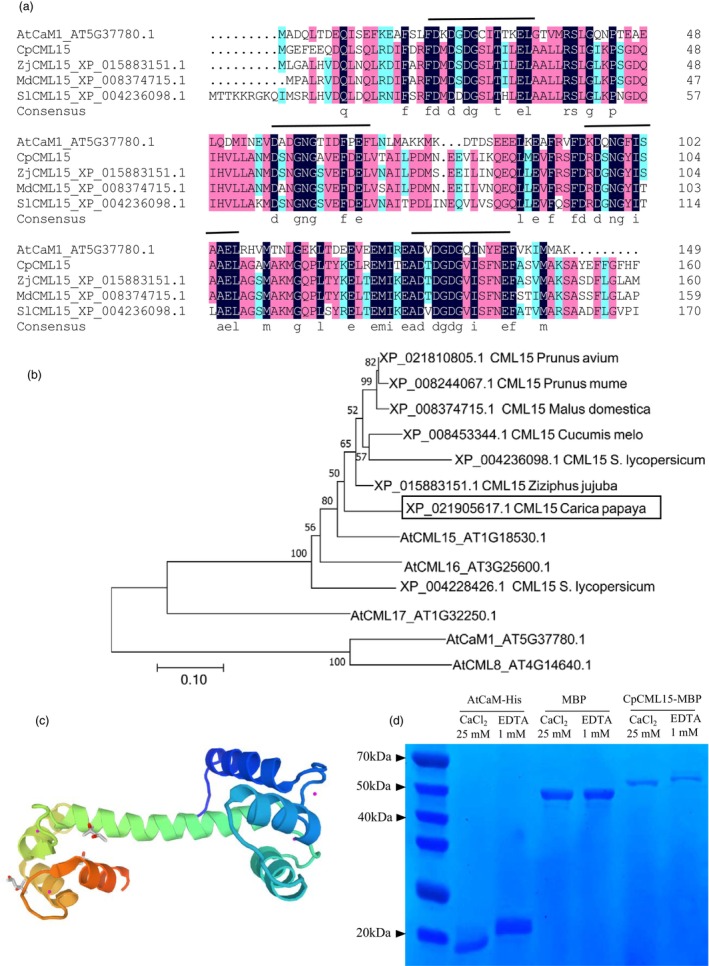
CpCML15, a calmodulin‐like protein, binds Ca^2+^. (a) Amino acid sequence alignment of CpCML15 and CMLs from other species. Sequence alignment was conducted using DNAMAN. The conserved and invariant residues are marked in pink and blue, and alignment gaps are presented as dashes. Domains of Ca^2+^‐binding EF‐hands for AtCaM1 and the CMLs are marked with a line. (b) Phylogenetic analysis of CpCML15 and CMLs from other plant species. A neighbour‐joining phylogenetic tree was constructed using the MEGA7.0 program. (c) Protein structure prediction of CpCML15 generated using the SWISS–MODEL Homology Modelling server (swissmodel.expasy.org). The pink dot indicates Ca^2+^. (d) Ca^2+^‐dependent electrophoretic mobility analysis of recombinant CpCML15. Purified proteins of recombinant AtCaM1‐His (positive control), recombinant maltose‐binding protein (MBP) (negative control), and CpCML15‐MBP were electrophoresed on SDS‐PAGE in the presence of 1 mm EGTA or 25 mm CaCl_2_. Protein bands were stained and visualized with Coomassie Brilliant Blue. Molecular weight markers (MW, kDa) are presented on the left.

The open reading frame (ORF) of *CpCML15* was 486 bp in length and encoded a CML Ca^2+^‐binding protein of 161 amino acid residues. The predicted molecular mass of CpCML15 was 20.3 kDa. BLAST in NCBI showed that *CpCML15* shared 81.65%, 80.96%, and 80.97% similarity with *Cucurbita maxima CML15* (XM_023150830.1), *Cucurbita moschata CML15* (XM_023077709.1), and *Benincasa hispida CML15* (XM_039024238.1), respectively. A BlastP search showed that CpCML15 contained four EF‐hand motifs, and showed high‐sequence identity with AtCaM1 and SlCML15 in *Arabidopsis* and tomato, respectively (Figure 2a).

As shown in Figure 2a, the conserved and identical residues were marked in blue and pink, respectively. Four EF‐hand motifs were predicted in CpCML15 and were also presented in Figure 2a via the amino acid sequence comparison, which was displayed with the underline marker. The phylogenetic analysis of CpCML15 and CMLs from other plants showed that CpCML15 had a close evolutionary relationship with CML15 in *Ziziphus jujuba*, tomato and *Arabidopsis* (Figure 2b). The predicted three‐dimensional protein structure of CpCML15 showed four EF‐loops and calcium binding sites (Figure 2c).

To demonstrate the Ca^2+^ binding ability of CpCML15, a sodium dodecyl sulfate polyacrylamide gel electrophoresis (SDS–PAGE) mobility shift assay was conducted. As shown in Figure 2d, the electrophoretic migration rates of a conserved CaM, AtCaM1, showed a faster migration rate in the presence of Ca^2+^ than in the presence of ethylenediaminetetraacetic acid (EDTA), a Ca^2+^ chelator (Figure 2d). Similar results were observed for CpCML15, in which the mobility of CpCML15 increased in the presence of Ca^2+^ compared to the presence of EDTA, indicating that CpCML15 acts as a calcium binding protein with Ca^2+^ binding ability to trigger Ca^2+^‐dependent conformational change (Figure 2d). No migration shift was observed for the control using maltose‐binding protein (MBP) with Ca^2+^ or with EDTA. All these results showed that CpCML15 could be considered as a Ca^2+^ sensor candidate in plant cell signalling.

### CpCML15 interacted with CpPP2C46/65

**Figure 3:**
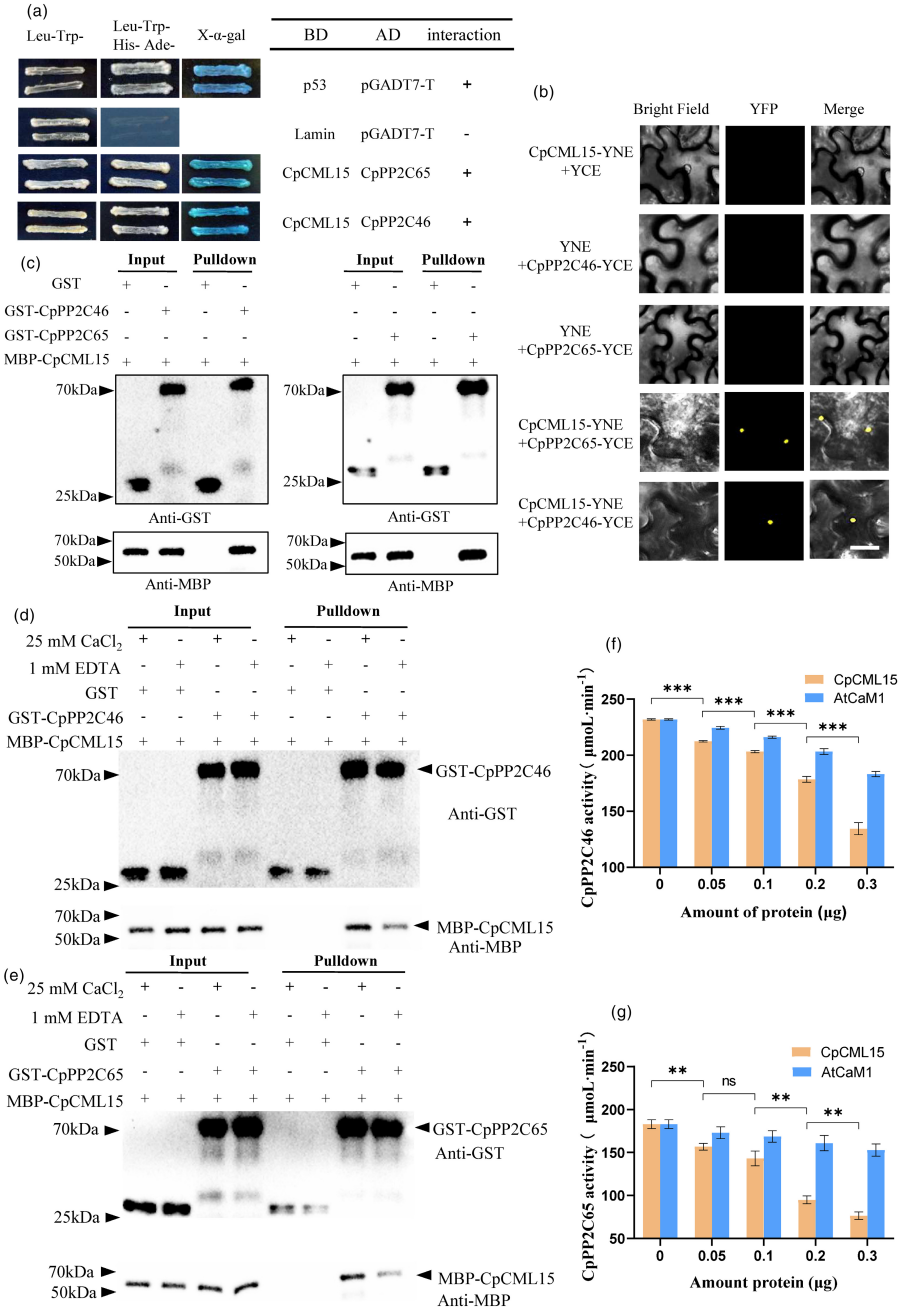
CpCML15 physically interacts with CpPP2C46/65 and inhibits the phosphatase activity of PP2C46/65. (a) Yeast two‐hybrid (Y2H) assays show interactions between CpCML15 and CpPP2C46/65. *CpCML15* was introduced into the pGBKT7 vector. The CpCML15 recombinant protein served as a bait protein. CpPP2C46/65 were the prey proteins. The Y2H yeast strains were co‐transformed with CpCML15 + CpPP2C46/65. pGADT7‐T + pGBKT7‐53 or pGADT7‐T + pGBKT7‐lam were set as the positive and negative controls, respectively. SD medium for yeast growth was lacking Trp, His, Leu, and Ade. Blue plaques display the interaction of protein staining with *X‐α‐gal*. (b) BiFC assays show the interaction between CpCML15 and CpPP2C46/65 in tobacco leaves. CpCML15 and CpPP2C46/65 were fused with the N and C terminals of YFP. CpCML15 with either the C or CpPP2C46/65 with N terminal alone were set as the negative control. A photo of the overlay of light and fluorescence views was taken. Bars = 25 μm. (c) *In vitro* GST pull‐down assays show the interaction between CpCML15 and CpPP2C46/65. The CpCML15‐MBP protein was co‐incubated with GST‐CpPP2C46/65 or GST, and the interacting proteins were detected via immuno‐blotting assays by the anti‐MBP or anti‐GST antibody, respectively. (d, e) Effect of Ca^2+^ on *in vitro* protein interaction between CpCML15 and CpPP2C46/65. (f, g) Effect of protein interaction between CpCML15 and CpPP2Cs on the phosphatase activity of PP2C46/65. The assay was conducted with a phosphopeptide as substrate. The activity of PP2C46/65 was tested with different amounts of CpCML15 or AtCaM1 proteins. AtCaM1 was added as the negative control. Values represent the means ± SD of three biological replicates, ** and *** indicate statistical differences at the 0.01 and 0.001 level.

Indeed, CMLs have no other functional domain than the EF‐hand motifs (Figure 2a,c) and it is often necessary to identify the interacting proteins of CMLs, to better understand their functions. Hence, CpCML15 was then used as bait for screening in the yeast‐two‐hybrid (Y2H) cDNA library, to identify CpCML15 interacting protein. CpPP2C46/65 were identified via this screening. The Y2H assay was then conducted to validate the interaction. As shown in Figure 3a, yeast cells grown on SD/Trp‐Leu‐His‐Ade medium and were stained blue with *X‐α‐gal*, indicating that CpCML15 interacted with CpPP2C46/65 *in vivo*. Positive and negative controls were also performed to validate the experimental procedure (Figure 3a). The interactions of CpCML15 and CpPP2C46/65 were then validated using a bimolecular fluorescence complementation (BiFC) assay *in planta* (Figure 3b). A pull‐down assay was performed to further confirm the interaction between these proteins. Glutathione S‐transferase (GST)‐tagged CpPP2C46/65 (CpPP2C46/65‐GST) and MBP‐tagged CpCML15 were produced and the proteins were purified before the pull‐down assay. As shown in Figure 3c, the interaction between CpCML15 and CpPP2C46/65 was confirmed *in vitro* (Figure 3c).

### Calcium affected the interaction between CpCML15 and CpPP2C46/65

Next, we wonder whether the interaction of CpCML15 and CpPP2C46/65 is Ca^2+^‐dependent. As shown in Figure 3d, the GST‐pull‐down assay showed that CpPP2C46 interacted with CpCML15 with the present of CaCl_2_. In contrast, the signal intensity reduced in the presence of EDTA (Figure 3d). The same result was also observed for the case of CpPP2C65 and CpCML15 (Figure 3e). These results indicated that Ca^2+^ affected the interaction of CpCML15 and CpPP2C46/65, and their interaction may occur in a Ca^2+^‐dependent manner.

### CpCML15 interacted with and reduced the activity of CpPP2C46/65

To better understand the significance of these interactions, we analysed the phosphatase activity of CpPP2C46/65 in the presence of CpCML15, using the typical CaM protein AtCaM1 as a control. AtCaM1 did not interact with CpPP2Cs (Figure S2), so the slightly reduced activity was observed in Figure 3f,g. By contrast, the activities of CpPP2C46/65 significantly decreased with the increase of exogenous CpCML15 protein addition (Figure 3f,g). These *in vitro* results supported that CpCML15 could reduce the phosphatase activity of CpPP2C46/65 through interacting with them.

### Characterization of CpPP2Cs in papaya

**Figure 4:**
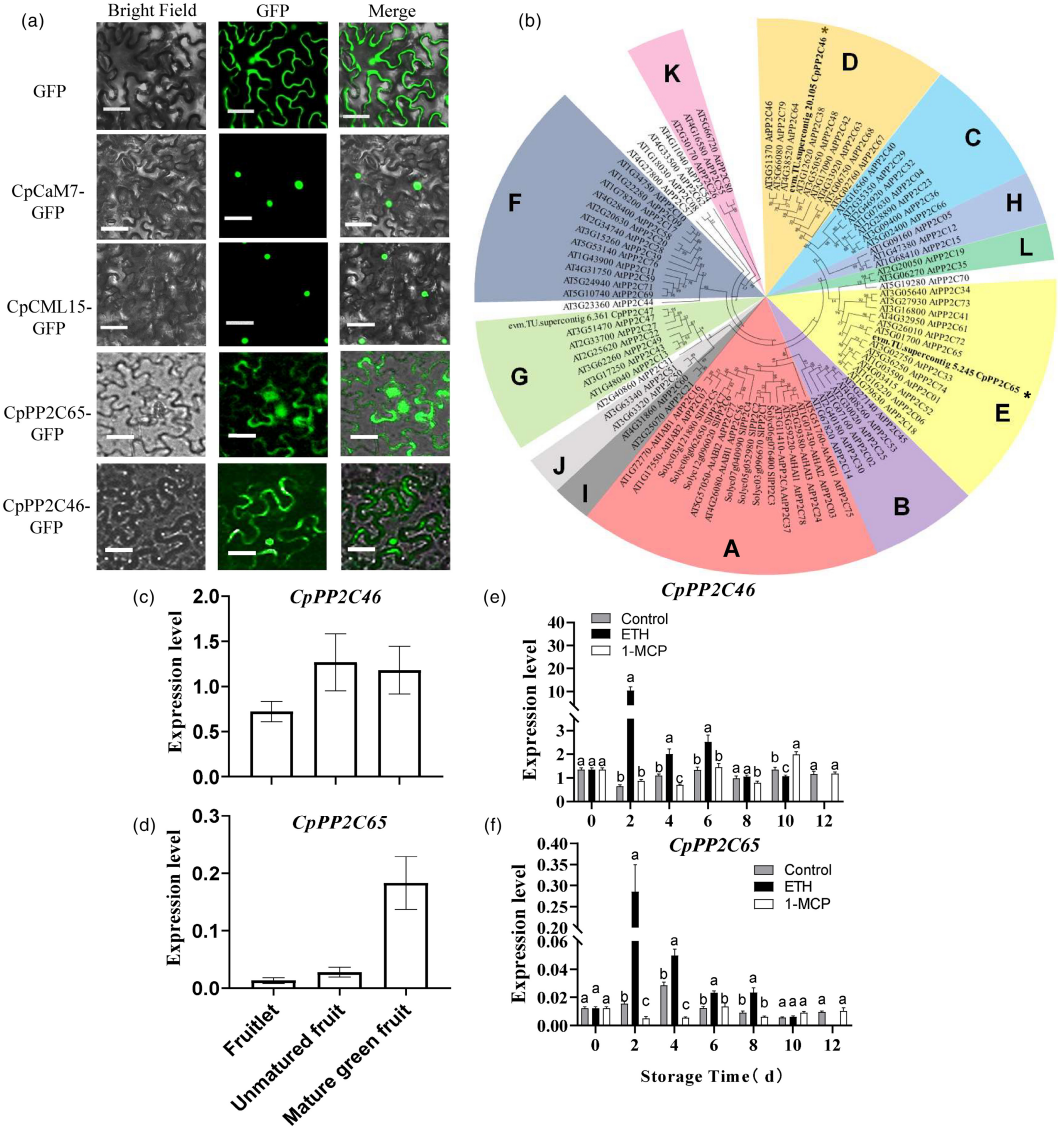
Characterization of CpPP2C46/65 in papaya. (a) Subcellular localization of CpCML15, CpCaM7 and CpPP2C46, and ‐65 in *N. benthamiana* leaves. The CpCML15, CpCaM7, CpPP2C46 and ‐65, ‐GFP fusion protein, and GFP control were injected and transiently expressed in *N. benthamiana* leaves. Then, images were obtained in the dark field for green fluorescence, and the merged images were taken in the bright field. Scale bars = 50 μm. (b) Phylogenetic analysis of CpPP2C46/65 and PP2Cs from *Arabidopsis* and tomato. The neighbour‐joining phylogenetic tree was constructed using the MEGA7.0 program (Sun *et al*., 2011). (c, d) The expression patterns of *CpPP2C46*/*65* during fruit development. Expression data on different development were relative to fruitlet, which was set as 1. (e, f) The expression patterns of *CpPP2C46*, and *‐65* under ethephon and 1‐MCP treatments by RT–qPCR. The *CpTBP1* and *CpTBP2* were used as reference genes. The gene expression level was related to the reference genes (geometric mean of *CpTBP1* and *CpTBP2*), which calculated by $2^{-\Delta C_{t}}$ formula. Data are presented as the means ± SD of three biological replicates. The different letters indicate statistical differences between treatments at the 5% level by one‐way analysis of variance (ANOVA).

To confirm the interaction of CpCML15 and CpPP2C46/65 in the nucleus as observed in Figure 3b, the subcellular localization of CpCaM/CMLs and CpPP2C46/65 in papaya was further determined. As shown in Figure 4a, fluorescence images from the *CpCaM7*‐GFP and *CpCML15*‐GFP constructs were detected in the nucleus of tobacco leaf cells, whereas those of the control GFP and CpPP2C46/65‐GFP were observed both in the cytomembranes and nucleus.

PP2C sequences from tomato and *Arabidopsis* were selected for multiple sequence alignment. As shown in Figure S3, based on multiple alignments of these selected representative protein sequences, CpPP2C46/65 contained most of the functional residues or domains that are well conserved within the protein family (Figure S3). Based on the phylogenetic analysis and the subclasses/subfamilies class according to the work of Sun *et al*. (2011) and Xue *et al*. (2008), CpPP2C46/65 belonged to clades D and E, but not the clade A, which contains most of conservative PP2Cs in the core ABA signalling pathway (Figure 4b), indicating different roles of CpPP2C46/65 from the clade A of PP2Cs.

Based on our previous RNA‐seq data, a group of *PP2C* genes was identified as differentially expressed after ethylene treatment during fruit ripening (Figure S4). The expression of *CpPP2C46/65* increased with fruit development process as validated by RT‐qPCR (Figure 4c,d), and the expression of *CpPP2C46* kept relatively stable during the storage under control condition, but induced by ethephon treatment. 1‐MCP repressed the expression during the early period (Figure 4e). The expression of *CpPP2C65* increased after the storage during early period and then decreased with storage, and dramatically induced by ethylene treatment, but severely repressed by 1‐MCP treatment (Figure 4f). These data suggested that the expression of *CpPP2C46/65* was positively related to fruit ripening. Additionally, the expression of genes related to ethylene and ABA biosynthesis and signalling pathways were also analysed. It was shown that the positive regulators in ethylene and ABA biosynthesis and signalling pathways were induced by ethylene treatment, but repressed by 1‐MCP treatment. On the contrary, the negative regulators were repressed by ethylene treatment, but induced by 1‐MCP treatment (Figure S5).

### Transient overexpression or silencing of *CpCML15* altered papaya fruit ripening

**Figure 5:**
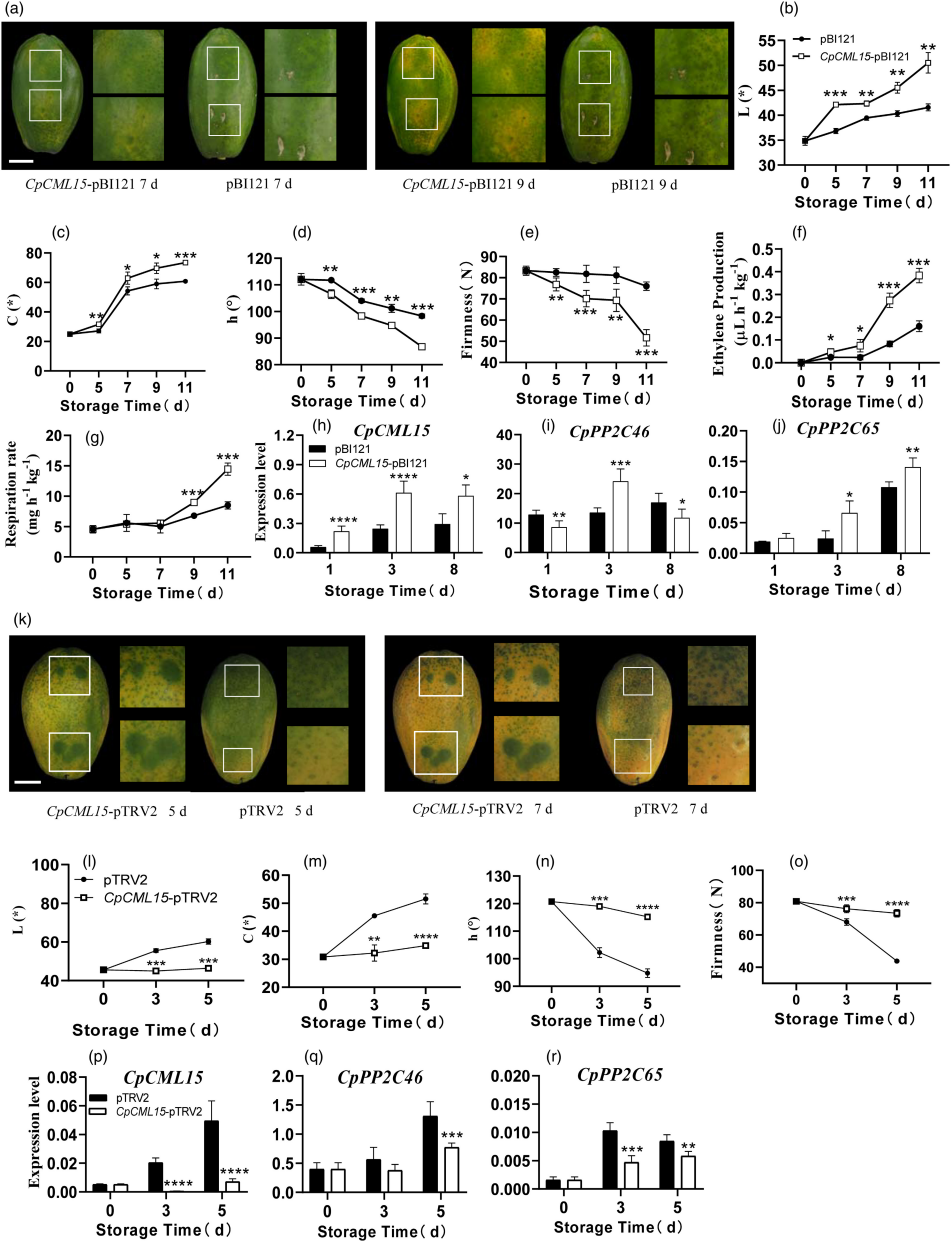
The transient overexpression or silencing of *CpCML15* in papaya altered fruit ripening process. (a) Transient overexpression of *CpCML15* (*CpCML15‐*pBI121) was used for overexpression with the 35S promoter; the empty pBI121 vector was used as the control. Bars = 30 mm. (b–g) The fruit colour including L (b), C (c), and h (d) values, firmness (e), ethylene production (f) and respiration rate (g). (h–q) Transcripts of *CpCML15*, *CpPP2C46/65* in the overexpression fruit around the injection sites were tested using RT–qPCR. (k) Papaya fruit transient silencing assays. Transient silencing of *CpCML15* (*CpCML15*‐pTRV2) was used for reduction with the pTRV2 vector. Empty pTRV2 vectors were set as controls. Bars = 30 mm. (l–o) The fruit colour including L (l), C (m) and h (n) values and firmness (o). (p–r) Transcripts of *CpCML15*, *CpPP2C46/65* were determined using RT‐qPCR in transient silencing fruit around the injection sites. The *CpTBP1* and *CpTBP2* were used as reference genes. The gene expression level was related to the reference genes (geometric mean of *CpTBP1* and *CpTBP2*), which calculated by $2^{-\Delta C_{t}}$ formula. Data are presented as the means ± SD of three biological replicates. Asterisks (*, **, *** and ****) present significant differences at the *P* < 0.05, *P* < 0.01, *P* < 0.001 and *P* < 0.0001 levels.

The function of *CpCML15* in fruit ripening was studied by transient overexpression assay and transiently silencing using VIGS system in papaya fruit (Figure 5). *CpCML15* overexpression accelerated fruit ripening, where the fruit displayed by a more rapid fruit yellowing than the control fruit (Figure 5a). The colouring index, including the L, C and h values, also showed a significant difference between the fruit overexpressing *CpCML15* and the control (Figure 5b,d). Compared to the control, the *CpCML15*‐overexpressing fruit also showed a faster rate of change in firmness and ethylene production and respiration rate (Figure 5f,g). Correspondingly, a higher expression levels of *CpCML15* was observed in the overexpressed fruit during ripening than in with the control fruit (Figure 5h). *CpPP2C46* and *CpPP2C65* expression were induced in the overexpressed fruit (Figure 5i,j). Additionally, the expression levels of ethylene synthetase related genes, including *CpACO1*, *CpACS10*, were upregulated in *CpCML15*‐overexpressing fruit (Figure S6a,b). The transcript levels of genes related to fruit softening, including *CpXYL*, *CpPG1*, *CpPG2*, *CpPME1* and *CpEXP1*, were significantly upregulated in the *CpCML15*‐overexpressing fruit compared to the control (Figure S6c,g).

On the contrary, fruit with transient silencing of *CpCML15* had delayed ripening phenotype (Figure 5k). Significant differences in colour changes were detected between the silenced fruit and the pTRV2‐empty vector control. Obvious green spots were observed at the injection sites in the silenced fruit compared to the control fruit, as well as at other sites without silencing (Figure 5k). The colouring index, including the L, C and h values, also showed a significant difference between the silenced fruit and the control fruit (Figures 5l,n). A significant higher firmness was observed in the silenced fruit than in the control fruit at different stages (Figure 5o). A significant decrease in *CpCML15* expression level was detected in the silenced fruit compared to the control fruit (Figure 5p). The expression of *CpPP2C46* and *CpPP2C65* were reduced in the silenced fruit compared to the control fruit (Figure 5q,r). Furthermore, the expression level of genes related to the ethylene synthesis pathway and fruit softening, was also reduced in the silenced fruit compared with the pTRV2‐empty vector control fruit, consistent with the fruit ripening phenotype (Figure S6h–n).

All these results indicated that *CpCML15* positively controlled the ripening and softening of papaya fruit, probably by indirectly regulating the transcription of genes related to ethylene and fruit softening.

### Transient overexpression or silencing of *CpPP2C46/65* altered papaya fruit ripening

**Figure 6:**
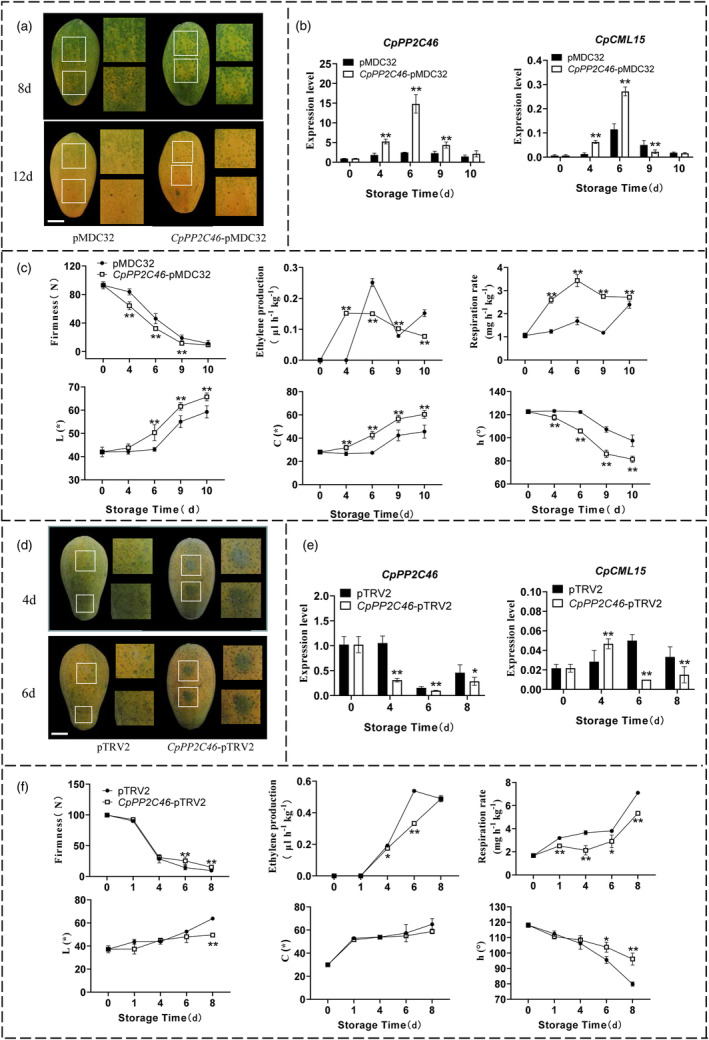
The altered expression (transient overexpression and silencing) of *CpPP2C46* in papaya fruit changed fruit ripening. (a) The construct of *CpPP2C46*‐pMDC32 was used for transient overexpression of *CpPP2C46* with the 35S promoter; the empty pMDC32 vector was used as the control. Bars = 30 mm. (b) Transcripts of *CpPP2C46* and *CpCML15* in the overexpression fruit around the injection sites were tested using RT–qPCR. (c) The fruit firmness, ethylene production, respiration rate and colour index including L, C and h values in the transient overexpression of *CpPP2C46* fruit during ripening. (d) Papaya fruit transient silencing assays. Transient silencing of *CpPP2C46* (*CpPP2C46*‐pTRV2) was used for reduction with the pTRV2 vector. Empty pTRV2 vectors were set as controls. (e) Transcripts of *CpPP2C46* and *CpCML15* in the silencing fruit around the injection sites were tested using RT–qPCR. (f) The fruit firmness, ethylene production, respiration rate and colour index including L, C and h values in the transient silencing of *CpPP2C46* fruit during ripening. The *CpTBP1* and *CpTBP2* were used as reference genes. The gene expression level was related to the reference genes (geometric mean of *CpTBP1* and *CpTBP2*), which calculated by $2^{-\Delta C_{t}}$ formula. Data are presented as the means ± SD of three biological replicates. Asterisks (* and **) present significant differences at the *P* < 0.05 and *P* < 0.01 levels.

**Figure 7:**
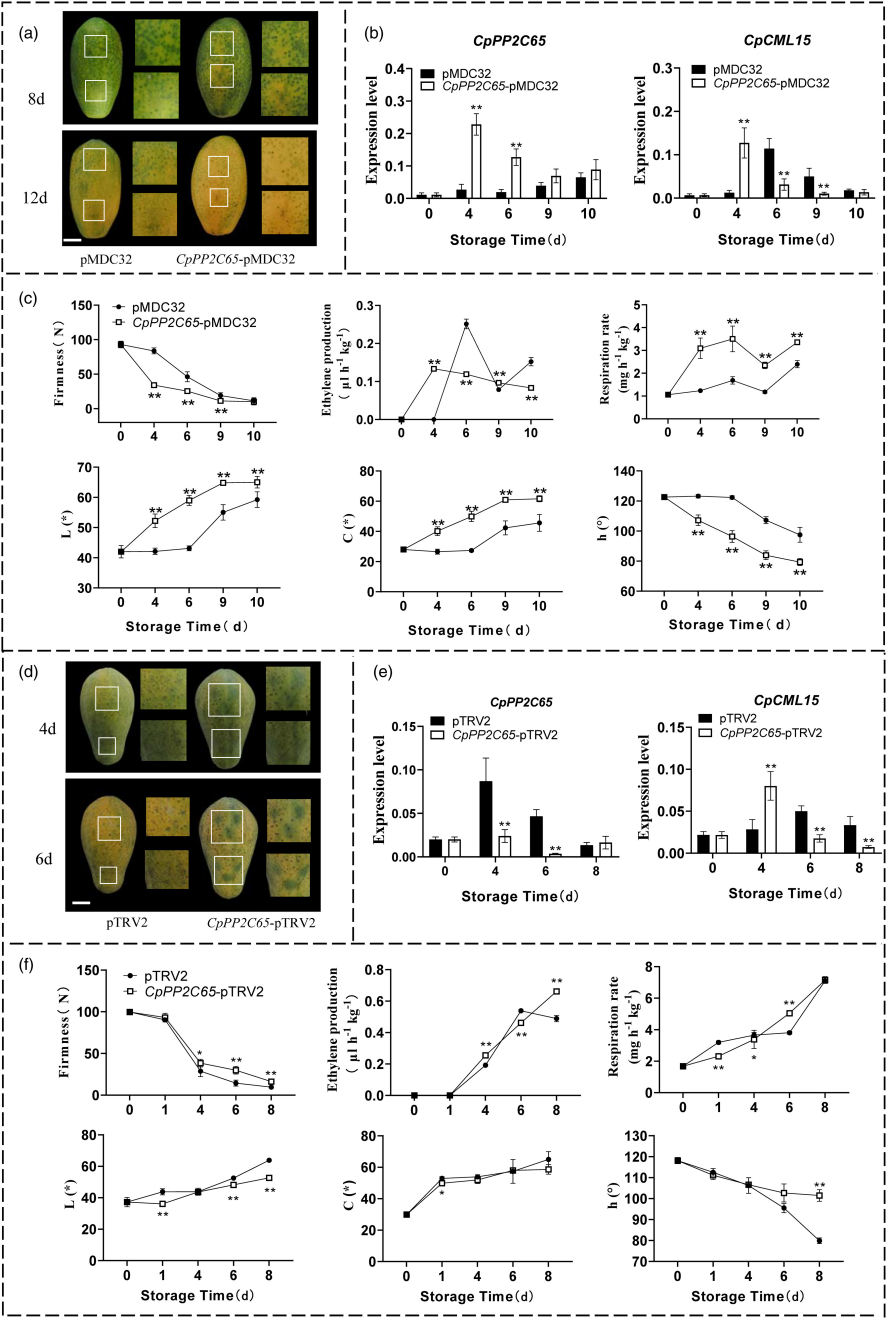
The altered expression (transient overexpression and silencing) of *CpPP2C65* in papaya fruit changed fruit ripening. (a) The construct *CpPP2C65‐*pMDC32 was used for transient overexpression of *CpPP2C65* with the 35S promoter; the empty pMDC32 vector was used as the control. Bars = 30 mm. (b) Transcripts of *CpPP2C65* and *CpCML15* in the overexpression fruit around the injection sites were tested using RT–qPCR. (c) The fruit firmness, ethylene production, respiration rate and colour index including L, C and h values in the transient overexpression of *CpPP2C65* fruit during ripening. (d) Papaya fruit transient silencing assays. Transient silencing of *CpPP2C65* (*CpPP2C65*‐pTRV2) was used for reduction with the pTRV2 vector. Empty pTRV2 vectors were set as controls. (e) Transcripts of *CpPP2C65* and *CpCML15* in the silencing fruit around the injection sites were tested using RT–qPCR. (f) The fruit firmness, ethylene production, respiration rate and colour index including L, C and h values in the transient silencing of *CpPP2C65* fruit during ripening. The *CpTBP1* and *CpTBP2* were used as reference genes. The gene expression level was related to the reference genes (geometric mean of *CpTBP1* and *CpTBP2*), which calculated by $2^{-\Delta C_{t}}$ formula. Data are presented as the means ± SD of three biological replicates. Asterisks (* and **) present significant differences at the *P* < 0.05 and *P* < 0.01 level.

The function of *CpPP2C46/65* in fruit ripening was also studied by transient overexpression and VIGS system in papaya fruit (Figures 6 and 7). *CpPP2C46/65* overexpression accelerated fruit ripening, and a more rapid fruit yellowing rate than the empty vector control fruit was observed (Figures 6a and 7a). Corresponding higher expression levels of *CpPP2C46* and *CpPP2C65*, as well as *CpCML15*, were observed in the overexpressing fruit during ripening compared to the empty vector control fruit (Figures 6b and 7b). A significant more rapid decreasing in the fruit firmness was observed in overexpressed fruit than in the empty control fruit at different stages (Figures 6c and 7c). The overexpression of *CpPP2C46/65* promoted the ethylene production and respiration rate compared to the control (Figures 6c and 7c). The colouring index, including the L, C and h values, also showed a significant difference between the overexpressing fruit and the control fruit (Figures 6c and 7c). Additionally, the expression levels of genes related to ethylene synthesis and fruit softening, including *CpACO1*, *CpACS10*, *CpEXP1*, *CpPG1*, *CpPG2*, *CpPME1* and *CpXYL*, were significantly upregulated in *CpPP2C46/65*‐overexpressing fruit compared to the control fruit (Figures S7a–g and S8a–g).

The significant differences in colour changes were detected between the silenced fruit of *CpPP2C46/65* and the empty vector control. Obvious green spots were observed at the injection sites in the silenced fruit compared to the pTRV2‐empty vector fruit, as well as at other sites without silencing (Figures 6d and 7d). A significant decrease in *CpPP2C46/65*, as well as the *CpCML15*, expression levels was detected in the silenced fruit compared to the pTRV2‐empty vector control fruit (Figures 6e and 7e). A significant difference was also displayed in the fruit firmness between silenced fruit and empty control fruit, with higher firmness observed in the silenced fruit compared to the empty vector control fruit at different stages (Figures 6f and 7f). The reduced expression of *CpPP2C46/65* also reduced or delayed the ethylene production and respiration rate than the empty control (Figures 6f and 7f), and the colouring index (L, C, h values) also showed a significant difference between the silenced fruit and the control fruit (Figures 6f and 7f). Furthermore, the expression level of genes related to the ethylene synthesis and fruit softening, including *CpACO1*, *CpACS10*, *CpEXP1*, *CpPG1*, *CpPG2*, *CpXYL* and *CpPME1*, was also reduced in the silenced fruit compared with the pTRV2‐empty vector control fruit, consistent with the fruit ripening phenotype (Figures S7h–n and S8h–n). These results showed that *CpPP2C46/65* positively regulate papaya fruit ripening and softening by probably indirectly affecting *CpCML15* expression and regulating the transcription of genes related to ethylene and softening.

### CpPP2C46/65 interacting with CpABI5 and CpERF003‐like to regulate papaya fruit ripening

**Figure 8:**
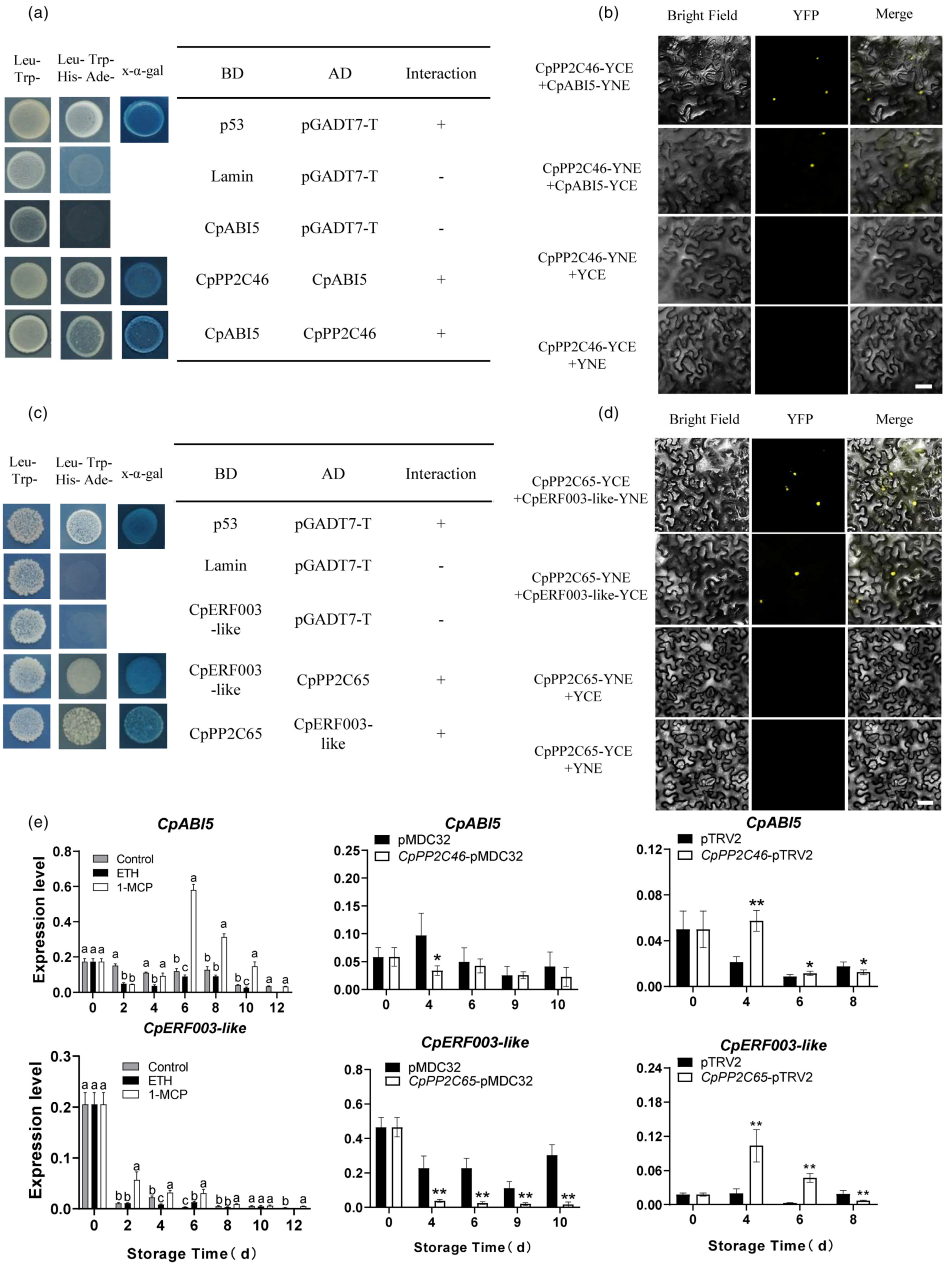
CpPP2C46 and CpPP2C65 physically interact with CpABI5 and CpERF003‐like, respectively. (a, c) Yeast two‐hybrid (Y2H) assays show interactions between CpPP2C46 and CpABI5 (a), CpPP2C65 and CpERF003‐like (c). The Y2H yeast strains were co‐transformed with CpPP2C46 + CpABI5 and CpPP2C65 + CpERF003‐like. pGADT7‐T + pGBKT7‐53 or pGADT7‐T + pGBKT7‐lam were set as the positive and negative controls, respectively. SD medium for yeast growth lack Trp, His, Leu, and Ade. Blue plaques display the interaction of protein staining with *X‐α‐gal*. (b, d) BiFC assays show the interactions between CpPP2C46 and CpABI5, CpPP2C65 and CpERF003‐like in tobacco leaves, respectively. CpPP2C46/65 and CpABI5/CpERF003‐like were fused with the N and C terminals of YFP. CpPP2C46/65 with either the C or N terminal alone was set as the negative control. A photo of the overlay of light and fluorescence views was taken. Bars = 50 μm. (e) The expression profile of *CpABI5* and *CpERF003‐like* under ethephon and 1‐MCP treatments, the transient overexpression and silencing of *CpPP2C46* and *CpPP2C65* fruit during fruit ripening. The *CpTBP1* and *CpTBP2* were used as reference genes. The gene expression level was related to the reference genes (geometric mean of *CpTBP1* and *CpTBP2*), which calculated by $2^{-\Delta C_{t}}$ formula. Data are presented as the means ± SD of three biological replicates. *, ** indicate statistical differences at the 0.05 and 0.01 levels. The different letters indicate statistical differences between treatments at the 5% level.

The Y2H assay was then conducted for the screening of target proteins of CpPP2C46/65. As shown in Figure 8a, yeast cells grew on SD/Trp‐Leu‐His‐Ade medium and were stained blue with *X‐α‐gal*, indicating that CpPP2C46 interacted with CpABI5 *in vivo*. The interaction of CpPP2C46 and CpABI5 was then validated using a BiFC assay. As shown in Figure 8b, significant YFP fluorescence signals were detected in the nucleus with co‐transformation of *CpPP2C46* and *CpABI5*, while no YFP fluorescence signal was detected with expression of a single protein. The Y2H and BiFC assays also showed that CpPP2C65 interacted with CpERF003‐like (Figure 8c,d). Gene expression analysis showed that both *CpABI5* and *CpERF003‐like* decreased with papaya fruit ripening, and was induced by 1‐MCP treatment but repressed by ethephon treatment (Figure 8e). The expression of *CpABI5* and *CpERF003‐like* was also repressed in the overexpressed fruit, but induced in the silenced fruit of *CpPP2C46* and *CpPP2C65*, respectively (Figure 8e). These results indicated that *CpABI5* and *CpERF003‐like* might be negatively related to fruit ripening.

### Overexpression of *CpCML15* in tomato accelerated fruit ripening

**Figure 9:**
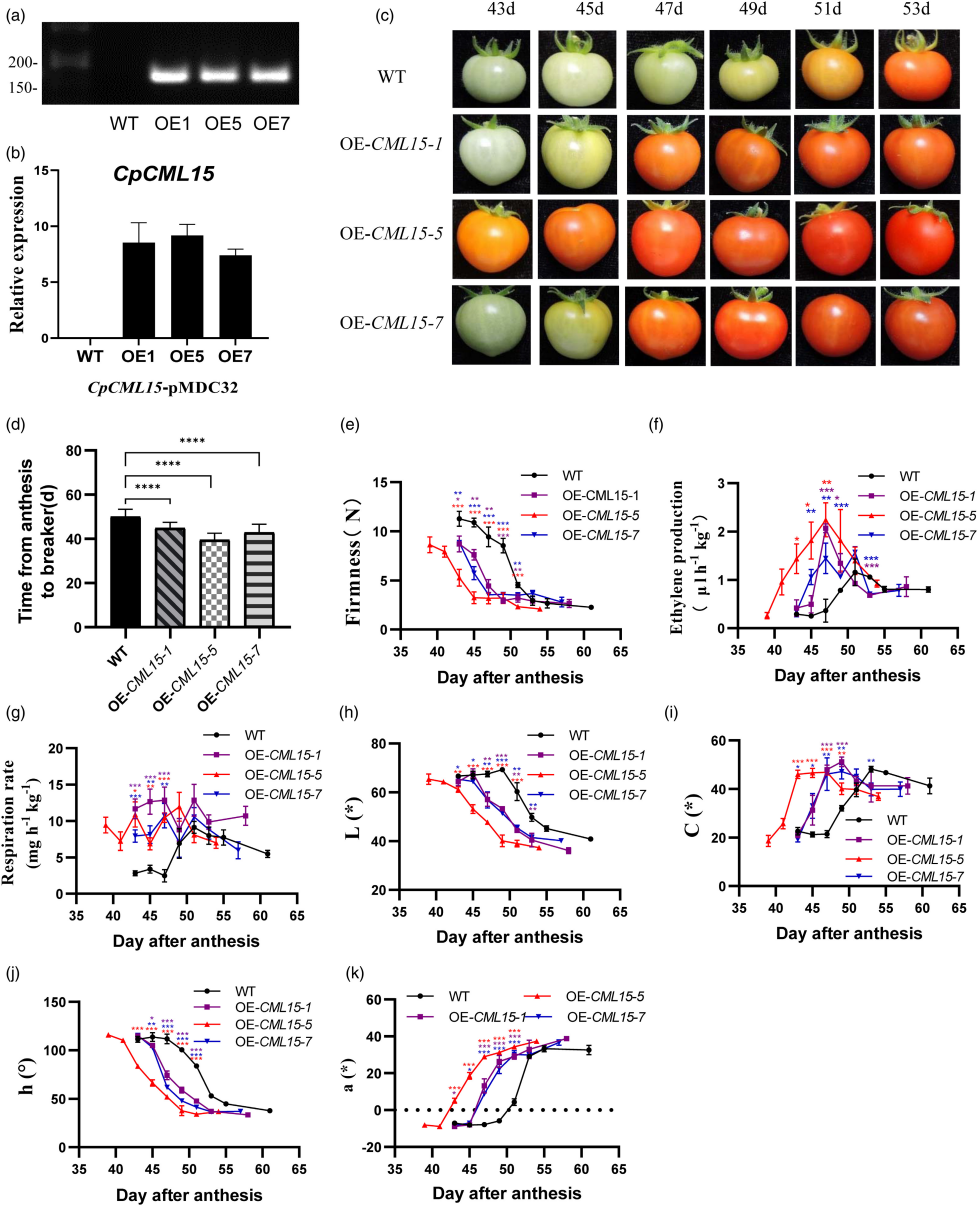
Overexpression of *CpCML15* in tomato accelerates fruit ripening and promotes fruit colouring and softening. (a–b) The transcriptional level of *CpCML15* in the fruit of the wild‐type (WT) and *CpCML15‐*overexpressing lines (OE1, OE5 and OE7) was detected by RT–PCR (a) and RT–qPCR (b). Pericarp tissues of *CpCML15*‐overexpressing and WT fruit at 30 dpa were used for the experiments. The gene expression profiles were normalized using the reference gene of *SlUBI*. (c) Fruit ripening phenotype of WT and *CpCML15*‐overexpressed lines. (d) Days from anthesis to the breaker stage of WT and *CpCML15*‐overexpressing lines. (e–k) Changes in fruit firmness (e), ethylene production (f), respiration rate (g) and colouring index (h–k) in WT and *CpCML15*‐overexpressing lines during fruit ripening. Asterisks (*, **, ***, ****) indicate significant differences at the 0.05, 0.01, 0.001 and 0.0001 levels.

Given the similar expression patterns of *SlCML15* and *CpCML15* during fruit ripening, we considered testing the knowledge gained from papaya on tomato, to further confirm the possible common role of *CML15* in fruit ripening. Therefore, *CpCML15* was further stably overexpressed in Micro‐Tom tomato. More than 10 independent transgenic lines were obtained, exhibiting consistent phenotypes in which fruit ripening was promoted. Three independent *CpCML15*‐overexpressing lines (OE1, OE5, and OE7) were selected for further investigation with high levels of *CpCML15* expression (Figure 9a,b). All *CpCML15* overexpressing lines promoted tomato fruit ripening (Figure 9c,d). The time from days post‐anthesis (dpa) to the breaker (BR) stage was significantly different between OE1/5/7 and WT lines. OE5 showed the highest *CpCML15* expression among these lines and the earliest maturation line compared with the others, with about 39 dpa to the BR stage. The maturity time was about 45 days for OE1/7 lines and 49 days for the WT (Figure 9d). Additionally, the decrease in firmness was accelerated in the OE lines compared to the WT (Figure 9e). *CpCML15* overexpression promoted ethylene production where the OE lines showed earlier and higher ethylene peaks compared to the WT (Figure 9f). The colour changes were also monitored and accelerated in the OE lines compared with the WT (Figure 9h–k).

### *CpCML15* overexpression altered the transcriptional level of genes related to fruit ripening

RNA‐seq was performed on WT, OE1 and OE5 tomato fruit at BR and BR + 2 days stages to further study the effect of CpCML15 on the transcriptional level during fruit ripening. General information of the sequencing statistics was presented in Table S1 and Figure S9a, indicating that the accuracy and quality of the data were sufficient for further analysis. A large number of DEGs were screened out between the overexpressing lines and WT at different ripening stages (Figure S9b–e). A total of 2045 DEGs were identified in the OE1 and OE5 lines compared with the WT in the BR and BR + 2 days stages (Figure S10a). GO classification analysis showed that carbohydrate metabolic process and integral component of membrane, cell wall binding, and catalytic activity were the most frequently enriched pathways in the comparison of the WT and *CpCML15*‐OE lines (Figure S11). The KEGG classification of these DEGs showed that plant–pathogen interaction (11.96%), MAPK signalling pathway (8.76%), plant hormone signal transduction (8.62%) and phenylpropanoid biosynthesis pathways were the most abundant (Figure S10b). The above DEGs were enriched in the KEGG pathways of the MAPK signalling pathway and photosynthesis, flavonoid biosynthesis, butanoate metabolism, phenylpropanoid biosynthesis and amino sugar and nucleotide sugar metabolism (Figure S10c). We selected genes closely related to fruit ripening, which are involved in abscisic acid, ethylene synthesis or signal transduction, calcium sensor genes and cell wall degradation, for expression analysis, which showing that most of these genes were upregulated during the ripening (Figure S10d).

### Overexpression of *CpCML15* and *CpPP2C46* altered ABA/ethylene sensitivity of tomato

As shown above, CpCML15 interacted with CpPP2C46/65, which not function as the group A PP2Cs in core ABA signal pathway, but may be involved in other ABA signals in regulating fruit ripening. We then wondered whether ABA sensitivity could be altered by *CpCML15* and *CpPP2C46/65* manipulation. Firstly, we tested the ABA sensitivity in the *CpCML15*‐overexpression tomato seedlings. The germination of *CpCML15*‐OE seeds was accelerated, and was less sensitive to exogenous ABA (Figure S12a–c). Additionally, the primary root growth of *CpCML15*‐OE seedlings was less sensitive to exogenous ABA treatment compared with that of WT seedlings (Figure S13a,b). It is indicated that overexpression of *CpCML15* alters the ABA sensitivity by affecting the ABA signalling pathways. CpPP2C46/65 are putative components in ABA signalling, so we tested tomato seedlings with *CpPP2C46* overexpression just as we did with *CpCML15*‐OE lines (data now shown). Similarly, the germination of *CpPP2C46‐*OE seeds was accelerated, and was less sensitive to exogenous ABA (Figure S12a,d–e). And the primary root growth of *CpPP2C46‐OE* seedlings was less sensitive to exogenous ABA treatment compared with that of WT seedlings (Figure S13a,c). These results indicated the heterologous overexpression of *CpPP2C46* altered the ABA sensitive and might act as a negative regulator in ABA signalling of tomato seed germination and root development.

The overexpression of *CpCML15* also altered the sensitivity of tomato to ethylene. Under the control condition, there is no difference in the hypocotyl elongation between WT and *CpCML15*‐OE lines. However, *CpCML15*‐OE seedlings displayed an enhanced triple response when treated with 10 μm 1‐aminocyclopropane‐1‐carboxylic acid (ACC) than the WT seedlings, with shorter hypocotyl elongation than the WT (Figure S14), but longer hypocotyl was observed for the *CpCML15*‐OE seedlings than WT under 1‐MCP treatment, suggesting the enhanced ethylene sensitivity in the *CpCML15*‐OE lines (Figure S14).

## Discussion

Ca^2+^ is a crucial regulator in plant development and stress response. CMLs are proteins that share the typical EF hands domain for Ca^2+^ binding without other known functional domains. CMLs are plant‐specific calcium sensors with a super‐gene family. So far, 50 and 32 *CML* genes have been reported in *Arabidopsis* and rice (Boonburapong and Buaboocha, 2007; McCormack and Braam, 2003), in addition to other species from algae to land plants (Zhu *et al*., 2015). The diversity of sequences and EF‐hand numbers may indicate the functional diversity of CMLs. However, most of the functions of CMLs in fruit ripening remain unclear. In the present work, we showed that a papaya CML, CpCML15, functioned in regulating the fruit ripening by interacting with PP2C proteins, the candidate components in the ABA signalling pathway. This finding significantly expanded the function of CML in plants.

The EF hands of CaM/CMLs have different affinities to Ca^2+^, and the binding affinities of Ca^2+^ are often altered by the interaction with partner proteins. In the present work, CpCML15 worked as an authentic Ca^2+^ binding protein that could acts as a calcium sensor in plants (Figure 2d). CML15 in *Arabidopsis* also showed a calcium‐binding characteristic and displayed calcium‐dependent conformational changes, acting as a real calcium sensor (Ogunrinde *et al*., 2017). Meanwhile, the identification of the target proteins is crucial to understanding the molecular mechanism of Ca^2+^ signal transduction. To date, CaM/CML targets in plants have been revealed to involved in various plant biological processes, including cell division, morphogenesis, ion transport, cell elongation and stress responses (Yang and Poovaiah, 2002).

The partner proteins of CaMs include kinases and phosphatases, transcription factors, ion channels, metabolic regulators and membrane transporters, such as MAPKs, WRKYs and IQD proteins (Poovaiah *et al*., 2013). For fruit ripening, a recent work reports that MaCaM could interact with a methionine sulfoxide reductase (Msr)‐MaMsrA7 and repair the sulfoxidated MaCaM1 in banana fruit. Moreover, MaCaM1 interacts with MaHY5‐1 and catalase (MaCAT1) to enhance the transcriptional repression activity of MaHY5‐1 on MaPSY2, and the catalytic activity of MaCAT1 to regulate fruit ripening (Jiang *et al*., 2018). For CMLs, a recent study has shown that AtCML24 interacts with WRKY46 and CAMTA2 to regulate ALUMINIUM‐ACTIVATED MALATE TRANSPORTER1 (ALMT1)‐dependent aluminium resistance in *Arabidopsis thaliana* and positively regulates Al stress response (Zhu *et al*., 2022). In rice, several target partners have been reported as binding targets of OsCML16, such as OsERD2 and OsCaM1, which are involved in calcium‐ and ABA‐mediated abiotic stress responses (Yang *et al*., 2020). In tomato fruit, SlCML37 cooperates with the partner proteasome maturation factor SlUMP1 and regulates fruit chilling stress tolerance (Tang *et al*., 2021). Actually, almost all of these CML target proteins have been identified in the model plants, and few have been reported in fruit during ripening process. In the present work, CpCML15 interacted with CpPP2C46/65 (Figure 3), which further target key members of the ABA and ethylene signal pathways. *CpPP2C46/65* expression and function analysis showed that they positively regulated fruit ripening (Figures 6 and 7). These findings greatly expanded our knowledge of the function of CMLs and their targets.

The clade A of PP2Cs, is the well‐studied group in plants, which negatively regulates ABA signalling by regulating the activity of the downstream target protein SnRK2 (Klingler *et al*., 2010), and the downstream partners of SnRK2 are mainly the basic leucine zipper (bZIP) transcription factors, including ABFs and AREBs. The targets of PP2Cs also include other transcription factors, such as MYB, MYC, NAC and MPK (Shen and Rose, 2014). PP2Cs of clade A affect fruit ripening by mediating the activity of SnRK2 and then a series of transcription factors (Shen and Rose, 2014; Zhang *et al*., 2018). AtABI1, a typical PP2C protein, interacts with key ethylene synthesis genes *AtACS2*, *AtACS6* and *AtACS7* and regulates its activity through dephosphorylation to regulate ethylene synthesis in *Arabidopsis* (Ludwików *et al*., 2014; Marczak *et al*., 2020). Inhibition of tomato *SlPP2C1* expression results in an increase in endogenous ABA content in fruit, which promotes ethylene release and cell wall metabolism, thus promoting tomato fruit ripening (Zhang *et al*., 2018). Further, a recent study reports that SlPPC3 in tomato interacts with SlSnRK2.8 to negatively regulate ABA signalling and fruit ripening (Liang *et al*., 2021). SlPP2C5 also belongs to the clade A, which functions as a negative regulator in ABA signalling during fruit development and ripening. Accordingly, *SlPP2C3/5*‐overexpressing fruit exhibited delayed ripening phenotype, whereas RNAi fruit accelerated fruit ripening, suggesting that *SlPP2C3/5* has an important function in fruit ripening (Liang *et al*., 2021). However, SAG113, a clade A PP2C member in *Arabidopsis*, negatively regulate ABA signal, but positively regulate leaf senescence, where *SAG113* knockout delayed leaf senescence but overexpression of *SAG113* accelerated leaf senescence (Zhang *et al*., 2012).

Most clade A PP2C members play negative roles in ABA signalling pathway, whereas BdPP2CA6, a clade A PP2C member, interacts with BdPYL11 in the absence of ABA, positively regulates ABA and stress signal pathway in transgenic *Arabidopsis* (Zhang *et al*., 2017). The *BdPP2CA6*‐overexpressing transgenic *Arabidopsis* seedling displayed ABA hypersensitive phenotype, with enhanced stomatal closure and salinity tolerance. A recent study also show that *OsPP108*, a clade A PP2C gene from rice, positively regulate abiotic stress response through ABA independent pathway, indicating multiple functions of clade A PP2Cs in stress signalling (Singh *et al*., 2015). Overexpression of *ZmPP2C2* can also greatly enhances the cold tolerance in tobacco under chilling stress (Hu *et al*., 2010). The overexpression of *FsPP2C2* from beech seeds (*Fagus sylvatica*) in *Arabidopsis* causes dwarf seedling and delayed flowering, with enhanced sensitivity to ABA and abiotic stress, suggesting that FsPP2C2 is a positive regulator of ABA signalling (Reyes *et al*., 2006). These results indicate the functional diversity of PP2Cs, even PP2Cs in clade A, in ABA signalling pathways in plants.

The expression pattern analysis indicated that some members of clade D in *Arabidopsis* and rice, might constitute positive regulators in ABA‐mediated signalling pathways (Xue *et al*., 2008). Our results also showed that CpPP2C46 belonged to the clade D and CpPP2C65 belonged to clade E (Figure 4b), which might play different roles than the clade A PP2Cs in regulating fruit ripening. This assumption was also validated by the gene functional analysis. Unlike several reported clade A PP2Cs (such as SlPP2C1, SlPP2C3 and SlPP2C5), which negatively regulate fruit ripening in tomato (Liang *et al*., 2021; Zhang *et al*., 2018), CpPP2C46/65 positively regulate fruit ripening (Figures 6 and 7). Similar to our work, recent work reports that a clade F PP2C, SlPP2C (Solyc07g062970) in tomato, positively regulates fruit ripening, as well as the leaf and flower senescence (Jiang *et al*., 2023). The expression of *SlPP2C* can be induced by ethylene and ABA. However, SlPP2C does not interact with SlPYLs like the clade A PP2Cs do (Jiang *et al*., 2023). Our study showed that seed germination and seedling primary root growth of *CpPP2C46‐*OE lines in tomato were enhanced, while they showed less sensitivity to exogenous ABA treatment compared with WT (Figures S12 and S13). These results indicated that heterologous overexpression of *CpPP2C46* altered the ABA sensitive and might act as a negative regulator in ABA signalling of tomato seed germination and root development. However, we still cannot conclude that CpPP2C46 is a negative regulator in ABA signalling, as the experiment was tested in *CpPP2C46* heterologous expression in tomato seedlings. The altered ABA sensitivity may due to the altered expression of tomato *PP2Cs* or to the function of CpPP2C46. Several ABA receptors such as CpPYL4/9, CpSRK2E/2A/CpSRK2A‐like, the homologous genes of ABA receptors PYLs and SnRK2s in *Arabidopsis*, were identified. However, the Y2H assay showed that there was no interaction between CpPP2C46/65 and CpPYL4/9 or CpSRK2E/2A/CpSRK2A‐like (Figure S15). Further works might be needed to validate the interaction of CpPP2C46/65 with other receptors. All these information indicated that PP2Cs in different groups could either negatively or positively regulate the ABA signalling, or involved in different ABA‐independent developmental processes.

The PP2C protein family is the main regulator of the interaction between Ca^2+^ and ABA signalling in stress responses (Edel and Kudla, 2016). PP2Cs can regulate various physiological activities of plants by interacting with the calcium‐sensing proteins CDPK and CBL‐CIPK of calcium signalling, regulating the activity of the slow anion channel 1‐S‐type anion channel SLAC1 (Edel and Kudla, 2016). In *Arabidopsis*, the PP2C encodes by the *ABI1* gene contains an EF motif that binds Ca^2+^ (Das *et al*., 1996). For fruit ripening, ABA interacts with calcium signals in mediating ethylene synthesis to regulate tomato fruit ripening (Xiong *et al*., 2021; Zhu *et al*., 2003). In the present work, CpABI5 and CpERF003‐like, two key transcriptional factors in the ABA and ethylene signalling pathways, were identified as target proteins of CpPP2C46 and CpPP2C65 (Figure 8a–d), respectively. The expression of *CpABI5* and *CpERF003‐like* were negatively related to fruit ripening (Figure 8e), indicating the negative roles of CpABI5 and CpERF003‐like in papaya fruit ripening.

Our results showed that calcium and ABA interacted and regulated papaya fruit ripening. Previous work has shown that exogenous calcium treatment delays fruit ripening (Gao *et al*., 2020). We also tested the effect of ABA on papaya fruit ripening. As shown in Figure S16, ABA treatment promoted fruit ripening, including colour change and ethylene production. The expression of *CpCML15* and *CpPP2C46/65* increased with fruit ripening and decreased during the later storage under the control condition. Their expression was induced by ABA treatment during the early period of ripening (Figure S17). The expression profiles of *ACO1*, *ACS10*, *ERF110* and genes related to fruit softening, such as *PG2*, *EXP1*, *PME1* and *XYL*, were also induced by ABA treatment (Figure S17). Differentially expressed genes (DEGs) in the ABA signalling were also analysed in fruit under 1‐MCP treatment, using RNA‐seq data (Zhu *et al*., 2019) and validated by RT‐qPCR. The expression levels of *PYR1/PYL*, *NCED3*, *CYP707A4‐like*, *PYL4‐like*, *ABIL1*, *PYL8*, and *ABF2‐like* were increased with fruit ripening and repressed by 1‐MCP treatment. However, the expression of other genes, such as *ABI5*, *ABIL46‐like*, *CYP707A1*, *ZEP* and *ABIL2‐like*, decreased during fruit ripening but was enhanced by 1‐MCP treatments (Figure S18). These DEGs may play key roles in papaya fruit ripening.

Protein phosphorylation is a common and important protein post‐translational modification, and it plays a very critical role in various biological processes (Kataya *et al*., 2019). Protein phosphorylation changes and regulates protein–protein interactions, protein stability, protein–DNA/RNA interactions and enzymatic activities, thereby affecting protein function. Some studies have reported that phosphorylation modification participates in the regulation of fruit ripening and senescence. For example, there are differences in protein phosphorylation levels at different developmental stages in strawberry, and a large number of phosphorylated proteins participate in the regulation of growth and fruit ripening (Lv *et al*., 2016). Many transcription factors in the ethylene signalling pathway have conserved phosphorylation modules (Li *et al*., 2009). Ethylene signal is transduced through protein phosphorylation events in plants, which in turn affects fruit ripening and plant stress responses (Raz and Fluhr, 1993). At the level of protein phosphorylation, ABA signalling and Ca^2+^ can regulate the common target proteins of kinases, such as RBOH proteins, which can be phosphorylated by CPK5, CBL1‐CIPK26, the calcium sensor proteins and the ABA signalling key factor SnRK2.6. The transcription factor ABI5/ABF1/ABF4 in the ABA signalling pathway is phosphorylated by CPK4/11/12/32, CIPK11/26 and SnRK2s (Edel and Kudla, 2016). The calcium sensor CIPK11/PKS5 regulates plant ABA signalling via ABI5 phosphorylation (Zhou *et al*., 2015). In the present work, we showed that the calcium sensor CpCML15 in papaya fruit could interact with CpPP2C46/65, which further target the CpABI5 in the ABA signalling pathway, and be involved in the fruit ripening. CpCML15 affected the phosphorylation level of fruit by dephosphorylating PP2C and affecting fruit ripening. A group of DEGs identified in the present work, including *CpACO1*, *CpABI5*, *CpERF003‐like*, *CpABIL46‐like*, *CpABIL1*, *CpABIL2‐like* and *CpABF2‐like*, could be candidate targets (Figures S5 and S17), and CpABI5 and CpERF003‐like were identified as the target protein of CpPP2C46 and CpPP2C65 (Figure 8), respectively. Actually, our previous work showed that various putative *cis*‐elements were identified in the promoters of key genes related to fruit softening, including *PG*, *PME*, *EXP1* and *XYL*, and most of them contained the binding motif of ERF and ABI, such as ABRE, W‐box motifs (Ding *et al*., 2018). It was proposed that CpPP2C46 and CpPP2C65 modulated the function of CpABI5 and CpERF003‐like by dephosphorylation, then regulating the activities of enzymes related to fruit softening and ripening. Further work will be conducted to confirm the function of these targets and the possible mechanism.

In conclusion, a possible model was proposed to explain the possible underlying mechanism of CML15‐PP2Cs‐ABI5/ERF003‐like pathway mediating papaya fruit ripening (Figure S19). ABA promoted papaya fruit ripening via ABA signalling pathway (Figures S16 and 17). Ethylene accelerated fruit ripening and induced the expression of *CpCML15* and *CpPP2C46/65* (Figure 1). CpCML15 interacted with CpPP2C46/65 and regulated their activity (Figure 3), thereby affecting their phosphorylation and modification of downstream target proteins CpABI5 and CpERF003‐like (Figure 8), or ACO (Figure S15b)/ACS (Ludwików *et al*., 2014; Marczak *et al*., 2020), further affecting the expression of downstream fruit ripening‐related genes (such as PG, PME…), and regulating fruit ripening. CpCML15 integrates the cross‐talk among the calcium, ABA and ethylene signals in regulating fruit ripening.

## Materials and methods

### Plant materials and treatments

Papaya fruit (*Carica papaya* L., cv. ‘Daqing’) at the mature green stage (about 120 days after anthesis, when fruit showed slight yellow around the blossom end/fruit top) were harvested from a commercial farm near Guangzhou, South China. The selected fruit were washed with water, soaked in hypochloride solution (0.2%, w/v) for 10 min, and then dipped in a 500 mg/mL mixture solution of prochloraz and iprodione (Huifeng and Kuaida, Jiangsu, China) for 1 min. Fruit were then divided into three groups under different treatments, including natural ripening (Control), delayed ripening by 1‐methylcyclopropene (100 nL/L 1‐MCP for 2 h, 1‐MCP), and accelerating ripening by ethephon (1000 μL/L ethephon, 1 min, ETH). Then, all fruit were packed with polyethylene film bags (thickness of 0.02 mm) without sealing and placed at 25 ± 1 °C for storage. Fruit pulp samples were taken at 0, 2, 4, 6, 8, 10 and 12 days, chopped, frozen in liquid nitrogen and stored at −80 °C. Three biological replicates were used in all treatments. Samples of different development stage, different size of the fruit during the development were also collected. The fruitlet samples were collected about 30 days after anthesis. The unmatured fruit were collected about 90 days after anthesis. The mature green fruit were collected about 120 days after anthesis, when fruit showed slight yellow around the blossom end/ fruit top.

### Yeast Two‐Hybrid assays and transcriptional activation analysis

To identify CpCML15 interacting protein, CpCML15 was used as bait for screening in the yeast‐two‐hybrid (Y2H) cDNA library, which constructed previously in the lab. CpPP2C46 and CpPP2C65 were screened out for the candidate interacting protein of CpCML15. For the interacting protein of CpPP2C46/65, we used the PP2Cs in *Arabidopsis* and papaya as the bait to screen in the string database (https://cn.string‐db.org/) for predicting the possible interacted protein. 31 transcription factors, plant hormone synthesis and plant hormone signalling transduction related proteins were found to have possible protein–protein interactions with 18 AtPP2Cs (Table S2). Afterwards, these proteins were BLASTP in NCBI (https://blast.ncbi.nlm.nih.gov/Blast.cgi) to find the protein with the highest homology in papaya. Then, we cloned the corresponding genes of papaya, and verify the protein interaction between these predicted protein and CpPP2C46/65 using Y2H test.

After screening, the Y2H assays were then performed as in our previous work by Song *et al*. (2022) for validation. Full‐length *CpCML15*, *CpABI5* and *CpERF003‐like* was introduced into the pGBKT7 vector and transformed into yeast cells. Colonies were cultivated on a medium lacking tryptophan (Trp), leucine (Leu), adenine (Ade), and histidine (His) and confirmed by staining with the chromogenic substrate *X‐α‐gal*. Full‐length *CpPP2C46/65* were ligated into the pGADT7 vector. Y2H yeast strains of CpCML15 and CpPP2C46, CpCML15 and CpPP2C65, CpABI5 and CpPP2C46, CpERF003‐like and CpPP2C65 were co‐transformed. The pGBKT7‐53 + pGADT7‐T and pGBKT7‐lam + pGADT7‐T were used as positive and negative controls, respectively. All primers used are listed in Table S3.

### Bimolecular fluorescence complementation (BiFC) assays

The BiFC assay was conducted as done in our previous work, following the method of Song *et al*. (2019). Briefly, the full‐length CDSs of *CpCML15*, *CpABI5*, *CpERF003‐like* and *CpPP2C46*, and ‐*65* were fused with pSP vectors using Gateway technology. These constructs were introduced into the *Agrobacterium* GV3101 strain. Combinations of different constructs were mixed in a 1 : 1 OD_600_ ratio and then injected into 3~4‐week‐old *Nicotiana benthamiana* epidermal cells. The fluorescence signal was recorded under confocal microscopy (Leica TCS SP5, Wetzlar, Germany) after 36 h of treatment. All primers used in the BiFC assay are listed in Table S3.

### Gel‐shift assay for recombinant proteins

Full‐length cDNA of *CpCML15* and *AtCaM1* was ligated into the pET‐28a vector. The resulting constructs and the pET‐GST were introduced into BM Rosetta (DE3) cells with 1.0 or 0.1 mm isopropyl‐b‐D‐thiogalactopyranoside (IPTG). CpCML15‐His and AtCaM1‐His were incubated overnight at 16 °C, and GST proteins were induced at 37 °C for 6 h (Figure S20). The full‐length cDNA of *CpCML15* was also ligated into the pMAL‐MBP vector. The resulting constructs were introduced into BM Rosetta (DE3) cells with 1.0 mm isopropyl‐b‐D‐thiogalactopyranoside (IPTG). CpCML15‐MBP was incubated 3 h at 37 °C (Figure S20).

The gel‐shift assay was perform as described by Tang *et al*. (2021). Briefly, the crude protein extracts were collected by soft centrifugation and sonication before protein purification with the His/MBP‐Tagged Protein Purification Kit (Clontech, Mountain View, CA, USA). The purified proteins were incubated with 50 mm CaCl_2_ or 50 mm ethylenediaminetetraacetic acid (EDTA) for 60 min, then boil with loading buffer for 10 min, and run on a 12.5% Tris‐glycine PAGE gel. The size and shift of the recombinant protein was analysed by staining with Coomassie brilliant blue.

### Pull‐down assay to verify protein interactions

Recombinant MBP‐tagged CpCML15 proteins was produced as previously described. Full‐length cDNA of *CpPP2C46/65* were ligated into the pET‐GST vector. The resulting constructs were introduced into BM Rosetta (DE3) cells with 1.0 mm isopropyl‐b‐D‐thiogalactopyranoside (IPTG). CpPP2C46, and ‐65‐GST proteins were induced at 28 °C for 6 h (Figure S20). The crude protein extracts were collected by soft centrifugation and sonication before protein purification with the GST‐tagged Protein Purification Kit (Clontech). Then, 5 μg each of CpCML15‐MBP and CpPP2C46/65‐GST proteins were incubated in binding buffer (1× PBS, 1% PMSF, 200 μL glutathione resin) for 8 h at 4 °C and separated by centrifugation. Proteins were washed with 1× PBS buffer at least 5 times. SDS loading buffer was added and boiled at 95~100 °C for 5 min, followed by SDS‐PAGE and Western blot. For Ca^2+^‐binding interactions between CpCML15 and CpPP2C46/65 assays, 25 mm CaCl_2_ or 1 mm EDTA was added to the binding buffer.

### Protein phosphatase assays

Recombinant His‐tagged CpCML15, AtCaM1 and GST‐tagged CpPP2C46/65 proteins were produced as previously described. The protein phosphatase assay was conducted using the serine–threonine phosphatase assay system (Promega, Madison, WI) according to the manufacturer's instruction. In brief, 10 μL of buffer and 5 μL substrate of phosphopeptide RRApTVA were added into 96‐well plates. Then 0.1 μg of CpPP2C46/65 proteins was added into plates, with different concentration of CpCML15 protein. Different concentration of AtCaM1 instead of CpCML15 protein were added as the control. Molybdate dye solution was added to the wells, incubated for 15 min at 25 °C. The absorbance at 630 nm was measured and the amount of phosphate released was calculated using a standard curve.

### Transient overexpression analysis of papaya fruit

The full CDSs of *CpCML15/CpPP2C46/65* were introduced into the pBI121 or pMDC32 vector. The *CpCML15/CpPP2C46/65‐*pPBI121/pMDC32 vectors were transformed into *A. tumefaciens* strain GV3101 using electroporation methods. Papaya fruit were harvested at the mature green stage (about 120 days after anthesis, when fruit showed slight yellow around the blossom end/fruit top), and infected according to Zhang *et al*. (2020). Then, the fruit were maintained at 22 °C with 90% relative humidity. The fruit colouring changes, firmness and gene expression were determined periodically. Gene expression was assayed periodically.

### Virus‐induced gene silencing in papaya fruit

The full CDS (486) of *CpCML15* cDNA and the 500 bp conserved fragment of the *CpPP2C46/65* genes were cloned into the pTRV2 vector. The *CpCML15/CpPP2C46/65*‐pTRV2 and pTRV2 empty constructs were then introduced into *A. tumefaciens*. Papaya fruit were harvested at the mature green stage and pre‐treated as described above, and all fruit were randomly divided into four separate groups. The mixture of *CpCML15/CpPP2C46/65*‐pTRV2 and pTRV2‐empty *A*. *tumefaciens* strain plus pTRV1 *A*. *tumefaciens* with a 1 : 3 ratio was injected into papaya fruit of different groups. All fruit groups were placed at 22 °C with 90% relative humidity. The fruit colouring changes, firmness and gene expression were determined periodically.

Other experimental methods are listed in Methods S1–S7.

### Statistical analysis

Each treatment contained independent biological triplicates. Data were analysed with variance analysis. The figures were drawn using Sigmaplot 12.0 software (San Jose, CA, USA). Ducan's multi‐range texts were used to compare the differences between the means of the groups. The data were plotted as the mean ± standard deviation (SD). The difference between groups with a *P* value <0.05 was regarded as significant.

## Funding

This work was supported by National Natural Science Foundation of China (32172640), Natural Science Foundation of Guangdong Province (2021A1515012435), the National Key Research and Development Program of China (2022YFD2100102), Science and Technology Program of Guangzhou (No. 201904010011) and Guangdong Pearl River Talents Program (2017GC010321).

## Authors' contribution

Xiaoyang Zhu designed the experiments; Qiunan Zhu, Qinqin Tan and Qiyang Gao conducted the experiments; Qiunan Zhu, Qinqin Tan and Senlin Zheng interpretated the data; Xiaoyang Zhu wrote the manuscript. Weixin Chen, Jean‐Philippe Galaud and Xueping Li reviewed the manuscript. All authors approved the paper.

## Ethics approval and consent to participate

The experiments did not involve endangered or protected species. No specific permits were required for these activities.

## Conflict of interest statement

The authors declare that the research was conducted in the absence of any commercial or financial relationships that could be construed as a potential conflict of interest.

## Significance statement

CpCML15 and CpPP2Cs positively regulate fruit ripening, integrating calcium, ABA and ethylene signals through the CpCML15‐CpPP2Cs‐CpABI5/CpERF003‐like pathway. This discovery greatly extends the knowledge of multiple signal cross‐talk in fruit ripening regulation.

## Supporting information


**Method S1** ABA treatment.
**Method S2** CpCML15 and CpPP2Cs protein evolutionary analyses and sequence analyses.
**Method S3** Subcellular localization analysis.
**Method S4** Tomato transformation.
**Method S5** Seed germination and primary root growth assay.
**Method S6** Triple‐response assay.
**Method S7** RNA‐seq analysis and RT–qPCR expression validation analysis.

**Table S1** The general information of sequencing statistics of all samples.

**Table S2** Predicted interacted proteins of CpPP2C46/65.

**Table S3** Primers used in present work.

**Figure S1** The expression profile of *SlCML15* during fruit development (a) and under ACC treatment (b). The RNA‐seq data for *SlCML15* were obtained from the TomExpress database (http://tomexpress.toulouse.inra.fr). DPA: days post‐anthesis.
**Figure S2** Y2H assay showed that AtCaM1 do not interact with CpPP2C46/65.
**Figure S3** Sequences analysis of CpPP2C46/65.
**Figure S4** Heatmap of the expression profiles of *CpPP2C*s during fruit ripening.
**Figure S5** Effect of 1‐MCP and ethephon treatments on the transcription of genes involved in ethylene and ABA synthesis and signal pathway.
**Figure S6** The transient overexpression or silencing of *CpCML15* in papaya fruit altered the expression of genes related to ethylene and fruit softening.
**Figure S7** The transient overexpression or silencing of *CpPP2C46* alters the expression profiles of genes associated with ethylene and fruit softening during fruit ripening.
**Figure S8** The transient overexpression or silencing of *CpPP2C65* alters the expression profiles of genes associated with ethylene and fruit softening during fruit ripening.
**Figure S9** Samples correlation map and volcano map show the distribution of significant DEGs.
**Figure S10** Overexpression of *CpCML15* alters the transcript profiles of tomato fruit.
**Figure S11** Comparison of the DEGs of the WT and *CpCML15*‐OE lines in GO classification.
**Figure S12** The heterologous overexpression of *CpCML15* and *CpPP2C46* in tomato altered plant sensitivity to ABA‐mediated inhibition of seeds germination.
**Figure S13** The heterologous overexpression of *CpCML15* and *CpPP2C46* in tomato altered plant sensitivity to ABA‐mediated inhibition of primary root growth.
**Figure S14** The triple‐response experiment of CpCML15‐OE and WT lines.
**Figure S15** Y2H was used to verify the interactions of CpCML15 (a), CpPP2C46 (b) and CpPP2C65 (c) with proteins in ethylene synthesis and ABA signal transduction pathway. SD medium for yeast growth was lacking Trp, His, Leu and Ade. Blue plaques display the interaction of protein staining with *X‐α‐gal*.
**Figure S16** The ABA treatment accelerated the fruit ripening.
**Figure S17** Transcripts of *CpCML15* and *CpPP2C46/65* and other genes involved in ethylene signal and fruit softening in fruit after ABA treatment were determined by RT–qPCR.
**Figure S18** The expression profiles of differential expression genes in ABA pathways under different 1‐MCP treatments.
**Figure S19** A proposed model of CML15‐PP2Cs‐ABI5/ERF003‐like on papaya fruit ripening.
**Figure S20** The recombinant proteins of CpCML15‐His (a), CpCML15‐MBP (b), CpPP2C46‐GST (c), CpPP2C65‐GST (d) and GST (e). SDS–PAGE gel stained with coomassie blue demonstrating affinity purification of the recombinant proteins. Lane 1: non‐induced protein; lane 2: induced protein; lane 3 (4, 5): after purification of then induced protein.

## Data Availability

All data that support the findings of this study are available from the corresponding author upon reasonable request.
