# Supplementary material for: Calmodulin‐like protein CML15 interacts with PP2C46/65 to regulate papaya fruit ripening via integrating calcium, ABA and ethylene signals
Source: Plant Biotechnol J. 2024 Feb 6;22(6):1703–23. doi: 10.1111/pbi.14297 (PMC11123395; doi:10.1111/pbi.14297)
Supplement: Supplementary file 1 — Method S1 ABA treatment. Method S2 CpCML15 and CpPP2Cs protein evolutionary analyses and sequence analyses. Method S3 Subcellular localization analysis. Method S4 Tomato transformation. Method S5 Seed germination and primary root growth assay. Method S6 Triple‐response assay. Method S7 RNA‐seq analysis and RT–qPCR expression validation analysis. [file PBI-22-1703-s004.docx]

**Supplemental Methods**

**Supplemental Method S1. ABA treatment**

Papaya fruit were pretreated as described in the main text. According to the pre-experiment results, an ABA solution with a concentration of 200 mg·L^−1^ and soaking for 30 min were used for ABA treatment. Fruit were then taken out and air-dried at ambient temperature, and a single fruit was packed in a polyethylene film bag (thickness of 0.02 mm) without sealing and placed at 25 ± 1°C. The experiment contained three replicates for each treatment, and each replicate contained 30 fruit. Fruit were periodically observed for physiological changes, including color index (L, C, h value) and ethylene production, as in our previous work (Gao et al. 2020). Samples were collected 0, 2, 4, 6, 8, and 11 days after treatment.

**Supplemental Method S2. CpCML15 and CpPP2Cs protein evolutionary analyses and sequence analyses**

Sequence alignment and phylogenetic tree analysis of CpCML15 and CpPP2Cs were conducted using CLUSTALW and MEGA7 software. The sequences of CpCML15 and CpPP2Cs from other species were extracted from the NCBI database (https://www.ncbi.nlm.nih.gov/). Protein alignment and conserved sequence identification were conducted and drawn using DNAMAN software.

**Supplemental Method S3. Subcellular localization analysis**

Full-length *CpCaM7*, *CpCML15*, and *CpPP2C46, and -65* cDNAs without the stop codon were ligated into pEAQ-GFP-HT vector. All primers used in the present work are presented in Table S3. The appropriate restriction site was selected in the vector, and connected with the gene amplification product, resulting in CpCaM7-GFP, CpCML15-GFP, and CpPP2C46, and -65-GFP fusion proteins. Leaves of similar size on 3- to 6-week-old *Nicotiana benthamiana* plants were selected and infiltrated with *Agrobacterium tumefaciens* strain GV3101 containing the pEAQ-GFP-HT constructs. The fluorescence signals were observed 36 h after infiltration using confocal laser scanning microscopy (Zeiss Axioskop 2 Plus). All fluorescence images were repeated at least three times. The empty pEAQ-GFP-HT vector that carries free GFP acts as an experimental control.

**Supplemental Method S4. Tomato transformation**

Tomato transformation for ectopic overexpression of *CpCML15* was performed following Song et al. ( 2022). Briefly, the full CDS of *CpCML15* was inserted into the pMDC32 vector, and the resulting constructs were introduced into *A*. *tumefaciens* strain GV3101 using electroporation methods. Tomato (*Solanum lycopersicum* L. cv. ‘Micro-Tom’) seedlings were used for transformation following standard methods (Fillatti et al. 1987). The positive plants were screened out on a hygromycin-containing medium and identified by the expression level of *CpCML15*. More than 10 positive transformed lines were obtained. Three representative independent positive lines (T2) were selected and used for the phenotype experiments. The firmness, color index, ethylene production, and respiration rate of the wild type (WT) and *CpCML15-*overexpressing tomato fruit were measured at different developmental stages. Samples at different developmental stages were collected and stored at -80°C.

**Supplemental Method S5. Seed germination and primary root growth assay**

Wild type and T3 generation *CpCML15*-OE and *CpPP2C46*-OE tomato seeds were washed and soaked in water for 6 h at room temperature. Tomato seeds were sterilized and sown in Murashige and Skoog (MS) medium containing 0 or 10 µM ABA (about 25 seeds per replicate), germinated at 25℃, and the germination rate was counted daily. For the primary root growth experiment, tomato seeds were first sowed in MS medium and grown for 3 days, and then the seedlings with the same primary root length were selected and transferred to MS medium containing 0 or 10 µM ABA and grown for 5 days.

**Supplemental Method S6. Triple-response assay**

The triple-response experiment was conducted as by Deng et al. (2022). The sterilized seeds of WT and *CpCML15*-OE lines were sowed on MS/2 medium and put at 4°C for 2 days, and then moved to 25°C for germination and keep in darkness for 3 days. Then the seedlings were transferred to MS/2 medium with or without 10 μM ACC, or treated with 1 µL L^–1^ of 1-MCP for 24-48h in darkness. The seedling triple-response was scored by assessing hypocotyl length, which was measured using image J software (https://imagej.nih.gov/ij/), and at least 50 seedlings were measured for each line.

**Supplemental Method S7. RNA-seq analysis and RT-qPCR expression validation analysis**

The WT tomato samples and *CpCML15* overexpression lines 1 and 5 (OE1, and OE5) at the breaker (BR) and BR + 2 (BR + 2 d) stages were selected for RNA-seq analysis. All cDNA libraries were prepared and sequenced using Biomarker Biotechnology Co., Ltd. (Beijing, China) on the Illumina Hi-Seq 2500 platform. RNA-seq analysis was conducted following the method used in our previous work (Zhu et al. 2019).

Gene expression analysis and RNA-seq validation were performed using quantitative real-time PCR analysis (RT-qPCR). The RT-qPCR experiment was conducted as described by Zhu et al. (2012). All primers used are presented in Table S3. The *CpTBP1* and *CpTBP2* were used as reference genes, as validated in our previous work (Zhu et al. 2012). The expression of the target genes was analyzed using the comparative CT method, which calculated using the 2^–ΔCt^ or 2^–ΔΔCt^ formula (Schmittgen and Livak, 2008). Three biological replicates were performed for the analysis.

**Reference:**

**Deng H., Chen Y., Liu Z., Liu Z., Shu P., Wang R., Hao Y., Su D., Pirrello J., Liu Y., Li Z., Grierson D., Giovannoni JJ., Bouzayen M., Liu M**. (2022). SlERF.F12 modulates the transition to ripening in tomato fruit by recruiting the co-repressor TOPLESS and histone deacetylases to repress key ripening genes. The Plant Cell. **34**:1250-1272.

**Fillatti J.J., Kiser J., Rose R., Comai L.** (1987). Efficient transfer of a glyphosate tolerance gene into tomato using a binary *Agrobacterium tumefaciens* vector. Nat. Biotechnol. **5:** 726-730.

**Gao Q., Tan Q., Song Z., Chen W., Li X., Zhu X.** (2020). Calcium chloride postharvest treatment delays the ripening and softening of papaya fruit. J. Food Process Pres. 44.

**Schmittgen, T., Livak, K. (2008).** Analyzing real-time PCR data by the comparative CT method. Nat. Protoc. **3:**1101-1108**.**

**Song Z., Lai X., Yao Y., Qin J., Ding X., Zheng Q., Pang X., Chen W., Li X., Zhu X.** (2022). F-box protein EBF1 and transcription factor ABI5-like regulate banana fruit chilling-induced ripening disorder. Plant Physiol. **188:** 1312-1334.

**Zhu X., Li X., Chen W., Chen J., Lu W., Chen L., Fu D.** (2012). Evaluation of new reference genes in papaya for accurate transcript normalization under different experimental conditions. Plos One **7:** e44405.

**Zhu X., Ye L., Ding X., Gao Q., Xiao S., Tan Q., Huang J., Chen W., Li X.** (2019). Transcriptomic analysis reveals key factors in fruit ripening and rubbery texture caused by 1-MCP in papaya. BMC Plant Biol. **19:**309.
