# Supplementary material for: Calmodulin‐like protein CML15 interacts with PP2C46/65 to regulate papaya fruit ripening via integrating calcium, ABA and ethylene signals
Source: Plant Biotechnol J. 2024 Feb 6;22(6):1703–23. doi: 10.1111/pbi.14297 (PMC11123395; doi:10.1111/pbi.14297)
Supplement: Supplementary file 4 — Table S3 Primers used in present work. [file PBI-22-1703-s003.docx]

**Table S3** Primers used in the present study.

| **Experiment** | **Primer name** | **Gene ID** | **Primer (5’-3’)** |
| --- | --- | --- | --- |
| RT-qPCR | CpTBP1-qPCR-F | JQ678780 | GGTAGTAGTAGTTAGGTATGTG |
|  | CpTBP1-qPCR-R |  | GGCAATCTGGTCTCACTT |
|  | CpTBP2-qPCR-F | JQ678779 | TGTGAATACTGGTGCTGAG |
|  | CpTBP2-qPCR-R |  | GGCATGAGACAAGACCTATA |
|  | SlUBI-qPCR-F | NM_001346406.1 | GCCGACTACAACATCCAGAAGG |
|  | SlUBI-qPCR-R |  | TGCAACACAGCGAGCTTAACC |
|  | CpCML15-qPCR-F | XM_022049925.1 | GAGAGTTCGAGGAGCAGGAT |
|  | CpCML15-qPCR-R |  | GCCGTTAGAGTCCATATTAGCA |
|  | CpPP2C46-qPCR-F | XM_022047980.1 | CTCTGCAAGAAGCAGCTAAGA |
|  | CpPP2C46-qPCR-R |  | CGAGGTTTGAGTCTAGGAACAC |
|  | CpPP2C65-qPCR-F | XM_022053045.1 | GAGAACTTGGCTGGTGAAGATA |
|  | CpPP2C65-qPCR-R |  | GACCTCTTGATGGCTGAATGA |
|  | CpACO1-qPCR-F | XM_022034413.1 | ATCTCGGGCTGGAAAAAGGG |
|  | CpACO1-qPCR-R |  | GCCGTCTTTGAGGAGTTGGA |
|  | CpACS10-qPCR-F | XM_022046047.1 | ACGATTTGTGGCAGGTTTGC |
|  | CpACS10-qPCR-R |  | AAAGCTCAAGCTCCCCCTTC |
|  | CpERF110-qPCR-F | XM_022042971.1 | CTGGTTCTGGTCTCTGGATTG |
|  | CpERF110-qPCR-R |  | GACCGCCTCTATAAACCCTTG |
|  | CpPG1-qPCR-F | XM_022056889.1 | TGGTGGTGCGTATAGATGGA |
|  | CpPG1-qPCR-R |  | ACAAAACCCAGTACCCACCA |
|  | CpPG2-qPCR-F | XM_022037696.1 | TCCTGAAGCTCACCCTTCAT |
|  | CpPG2-qPCR-R |  | CCTCAATGCCTTTGAAGCTC |
|  | CpEXP1-qPCR-F | XM_022054114.1 | GCTGTGGTTCTTGCTACGAA |
|  | CpEXP1-qPCR-R |  | TGCTGGAGAGGAGGATTGC |
|  | CpPME1-qPCR-F | XM_022051068.1 | TCTTCGGCATCTCCTACTTCC |
|  | CpPME1-qPCR-R |  | AGTACTGGTACAGAAGTGGATCTC |
|  | CpXYL-qPCR-F | XM_022036672.1 | GCTTCCGCTGTGTTTTATGG |
|  | CpXYL-qPCR-R |  | ATGATTGGATCGACCTCAGC |
|  | CpABIL1-qPCR-F | XM_022032117.1 | GCGGAACATTCACTGAGATAGA |
|  | CpABIL1-qPCR-R |  | CCCGTGCTTGTGGACTAAA |
|  | CpABI2-like-qPCR-F | XM_022039118.1 | AGCACCATAAGCGGTACATATT |
|  | CpABI2-like-qPCR-R |  | GTGAGTACTCTCCAGCACTAAAG |
|  | CpABIL46-like-qPCR-F | XM_022041004.1 | TGCCTCTCCTGTGCATTTAG |
|  | CpABIL46-like-qPCR-R |  | GCAATCCTCCAACCTGAACTA |
|  | CpABI5-qPCR-F | XM_022052748.1 | GTGAGAGAACAGTGTGCAGTAG |
|  | CpABI5-qPCR-R |  | CACGTTCGCATTGTTGGTATTT |
|  | CpABI5-like-qPCR-F | XM_022054167.1 | GGAATCAACGGCTCTCAGTATC |
|  | CpABI5-like-qPCR-R |  | TTCCACTGCCCTCCTCTTA |
|  | CpABF2-like-qPCR-F | XM_022055605.1 | GACACAAAGAGAAGCAGCATTG |
|  | CpABF2-like-qPCR-R |  | ATCTCAATTAACGGCGGGATAG |
|  | CpAAO-like-qPCR-F | XM_022036913.1 | CCGAGACAGGTTGGTGATTTA |
|  | CpAAO-like-qPCR-R |  | CAAGGGCAGTTTGTGTTTCC |
|  | CpCYP707A1-like-qPCR-F | XM_022036322.1 | CCCAACACTTTCATGCCATTT |
|  | CpCYP707A1-like-qPCR-R |  | GTGGTAAGATGATGGAGGAAGAC |
|  | CpCYP707A4-like-qPCR-F | XM_022042854.1 | CTCTACTCTCAGCACCCAAATC |
|  | CpCYP707A4-like-qPCR-R |  | GCATGAGTCACCAACACAAATC |
|  | CpGT-qPCR-F | XM_022053433.1 | CCTCCGTCTGAACAATCCTAAC |
|  | CpGT-qPCR-R |  | GAACGAACTACAGTGCAGAAGA |
|  | CpNCED3-qPCR-F | XM_022039358.1 | CCAGTAGGAAGCCCAACATATC |
|  | CpNCED3-qPCR-R |  | GTGTTTGGGAGGAGAGACAAT |
|  | CpPYR1/PYL-qPCR-F | XM_022055475.1 | TCTGGTCGATTGTTCGGCAA |
|  | CpPYR1/PYL-qPCR-R |  | TTCACCGTGCAGCTCTTGAT |
|  | CpPYL8-qPCR-F | XR_002541342.1 | TTGCGGTGCAAGATCGTACT |
|  | CpPYL8-qPCR-R |  | CTCCGAGATACGAAAGCCCC |
|  | CpPYL9-qPCR-F | XM_022056759.1 | TGGTCTCTGGTGAGGAGATT |
|  | CpPYL9-qPCR-R |  | GGACTTGACGTTGACTTCTCTC |
|  | CpPYL1-like-qPCR-F | XM_022055475.1 | GGTGAAGGAAGGGTTTAGGATT |
|  | CpPYL1-like-qPCR-R |  | CATCCAGTATGTCAAGCCTCTC |
|  | ACS1-like-qPCR-F | XM_022037840.1 | GAGTGACGTTCGACCCAGAG |
|  | ACS1-like--qPCR-R |  | TTCGCCATCTCAGGTCCCTA |
|  | ACO-h1-like1-qPCR-F | XM_022043900.1 | CCCAAGAATCTCTGTGGCGT |
|  | ACO-h1-like1-qPCR-R |  | CTCTGTAGACTGGCGGGTTG |
|  | ACO-h4-like-qPCR-F | XM_022036769.1 | TCCAGAAACTCACAGACGCC |
|  | ACO-h4-like-qPCR-R |  | CGTTTGGCTGCTCGTGAAAA |
|  | SRK2E-qPCR-F | XM_022040901.1 | GTCACTGAGGCATCCGAACA |
|  | SRK2E--qPCR-R |  | AAAAGCGTGCCTCATCCTCA |
|  | SRK2A-like-qPCR-F | XM_022034165.1 | AGGCTTTTGAGGCACAAGGA |
|  | SRK2A-like --qPCR-R |  | GCCAAATGGGTAGGGGTCAA |
|  | PYL4-qPCR-F | XM_022053138.1 | ATCCCCAGGCATACAAGCAC |
|  | PYL4-qPCR-R |  | TGTCTCTCGTCGTCCAGGAT |
|  | ERF003-like-qPCR-F | XM_022034979.1 | CGAAGCCGCTAGACTCATGT |
|  | ERF003-like-qPCR-R |  | AGCCATGTAACAGCGGTGAA |
|  | CpPYL4-like-qPCR-F | XM_022048356.1 | GGACAGTTGTCGTCGAATCATA |
|  | CpPYL4-like-qPCR-R |  | GCAAGTTGCAGCGAACAAT |
|  | CpZEP-qPCR-F | XM_022041950.1 | AGGTGGATGCATGGCTATCG |
|  | CpZEP-qPCR-R |  | CCTCCCTTCGAGATTCGAGC |
| RT-PCR | CpCML15-qPCR-F |  | GAGAGTTCGAGGAGCAGGAT |
|  | CpCML15-qPCR-R |  | GCCGTTAGAGTCCATATTAGCA |
| In vitro calcium binding / GST-pull down | CpCML15-pET28a-F |  | CAGCAAATGGGTCGCGGATCCATGGGAGAGTTCGAGGAGCAGGATC |
|  | CpCML15-pET28a-R |  | TGCGGCCGCAAGCTTGTCGACCGAGAAGTGAAAGCCAAAGA |
|  | CpCML15-pMAL-F |  | AGGGAAGGATTTCAGAATTCATGGGAGAGTTCGAG |
|  | CpCML15-pMAL-R |  | AGGTCGACTCTAGAGGATCCTCACGAGAAGTGAAA |
|  | AtCaM1-pET28a-F |  | CAGCAAATGGGTCGCGGATCCATGGCGGATCAACTC |
|  | AtCaM1-pET28a-R |  | TGCGGCCGCAAGCTTGTCGACCTTAGCCAT |
|  | CpPP2C46-pETGST-F |  | TTTCAGGGTCCAGCAGGATCCATGTTATCTGGGTTGATGAACTTTTTAAG |
|  | CpPP2C46-pETGST-R |  | ATGGTGGCTAGCGCTGAATTCCCAACAGAAGCCGTTGGCTAC |
|  | CpPP2C65-pETGST-F |  | TTTCAGGGTCCAGCAGGATCCATGGGAACCTGTTGTAGCAAGGAAC |
|  | CpPP2C65-pETGST-R |  | ATGGTGGCTAGCGCTGAATTCGGAACAGAATTGACTACTTTTC |
| Y2H | CpCML15-pGBKT7-F |  | ATGGCCATGGAGGCCGAATTCATGGGAGAGTTCGAG |
|  | CpCML15-pGBKT7-R |  | TGCGGCCGCTGCAGGTCGACGTCACGAGAAGTGAAA |
|  | AtCaM1-pGBKT7-F |  | ATGGCCATGGAGGCCGAATTCATGGCGGATCAACTC |
|  | AtCaM1-pGBKT7-R |  | GGCCGCTGCAGGTCGACGTCACTTAGCCAT |
|  | CpPP2C46-pGADT7-F |  | ATGGCCATGGAGGCCAGTGAATTCATGTTATCTGGGTTG |
|  | CpPP2C46-pGADT7-R |  | TGCAGCTCGAGCTCGATGGATCCCTCAGTAGCCAACGGC |
|  | CpPP2C65-pGADT7-F |  | ATGGCCATGGAGGCCAGTGAATTCATGGGAACCTGTTGT |
|  | CpPP2C65-pGADT7-R |  | TGCAGCTCGAGCTCGATGGATCCCTCAAAACACATGAGA |
|  | CpPP2C46-pGBKT7-F |  | ATGGCCATGGAGGCCGAATTCATGTTATCTGGGTTG |
|  | CpPP2C46-pGBKT7-R |  | TGCGGCCGCTGCAGGTCGACGTCAGTAGCCAACGGC |
|  | CpPP2C65-pGBKT7-F |  | ATGGCCATGGAGGCCGAATTCATGGGAACCTGTTGT |
|  | CpPP2C65-pGBKT7-R |  | TGCGGCCGCTGCAGGTCGACGTCAAAACACATGAGA |
|  | CpABI5-pGBKT7-F |  | ATGGCCATGGAGGCCGAATTCATGGAACTTAAAAAA |
|  | CpABI5-pGBKT7-R |  | TGCGGCCGCTGCAGGTCGACGTTACGGCCAACTCAC |
|  | CpERF003-like -pGBKT7-F |  | ATGGCCATGGAGGCCGAATTCATGGCCAGACCACAA |
|  | CpERF003-like -pGBKT7-R |  | TGCGGCCGCTGCAGGTCGACGCGTTGCCTGAGCCTG |
|  | CpABI5- pGADT7-F |  | ATGGCCATGGAGGCCAGTGAATTCATGGAACTTAAAAAA |
|  | CpABI5-pGADT7-R |  | TGCAGCTCGAGCTCGATGGATCCCTTACGGCCAACTCAC |
|  | CpERF003-like-pGADT7-F |  | ATGGCCATGGAGGCCAGTGAATTCATGGCCAGACCACAA |
|  | CpERF003-like-pGADT7-R |  | TGCAGCTCGAGCTCGATGGATCCCCGTTGCCTGAGCCTG |
| BiFC | CpCML15-pSP-F |  | GGCGCGCCACTAGTGGATCCATGGGAGAGTTCGAG |
|  | CpCML15- pSP -R |  | GCGGTACCCTCGAGGTCGACCGAGAAGTGAAA |
|  | CpPP2C46- pSP -F |  | GGCGCGCCACTAGTGGATCCATGTTATCTGGGTTG |
|  | CpPP2C46- pSP -R |  | GCGGTACCCTCGAGGTCGACGTAGCCAACGGC |
|  | CpPP2C65- pSP -F |  | GGCGCGCCACTAGTGGATCCATGGGAACCTGTTGT |
|  | CpPP2C65- pSP -R |  | GCGGTACCCTCGAGGTCGACAAACACATGAGA |
|  | CpABI5- pSP -F |  | GGCGCGCCACTAGTGGATCCATGGAACTTAAAAAA |
|  | CpABI5- pSP -R |  | CGGTACCCTCGAGGTCGACTTACGGCCAACTCA |
|  | CpERF003-like- pSP -F |  | GGCGCGCCACTAGTGGATCCATGGCCAGACCACAA |
|  | CpERF003-like- pSP -R |  | CGGTACCCTCGAGGTCGACCGTTGCCTGAGCCTG |
| Subcellular location | CpCML15-GFP-F |  | TATTCTGCCCAAATTCGCGACCGGTATGGGAGAGTTCGAG |
|  | CpCML15-GFP-R |  | AAAGTTCTTCTCCTTTGCTAGTCATCGAGAAGTGAAA |
|  | CpPP2C46-GFP-F |  | TATTCTGCCCAAATTCGCGACCGGTATGTTATCTGGGTTG |
|  | CpPP2C46-GFP-R |  | AAAGTTCTTCTCCTTTGCTAGTCATGTAGCCAACGGC |
|  | CpPP2C65-GFP-F |  | TATTCTGCCCAAATTCGCGACCGGTATGGGAACCTGTTGT |
|  | CpPP2C65-GFP-R |  | AAAGTTCTTCTCCTTTGCTAGTCATAAACACATGAGA |
| Transgenic tomato | CpCML15-pMDC32-F |  | GGGGACAAGTTTGTACAAAAAAGCAGGCTATGGGAGAGTTCGAG |
|  | CpCML15-pMDC32-R |  | GGGGACCACTTTGTACAAGAAAGCTGGGTTCACGAGAAGTGAAA |
| Transient overexpression | CpCML15-pBI121-F |  | AGAGAACACGGGGGACTATGGGAGAGTTCGAG |
|  | CpCML15-pBI121-R |  | GGTACCCTCGAGGTCACGAGAAGTGAAA |
|  | CpPP2C46-pMDC32-F |  | GGGGACAAGTTTGTACAAAAAAGCAGGCTATGTTATCTGGGTTG |
|  | CpPP2C46-pMDC32-R |  | GGGGACCACTTTGTACAAGAAAGCTGGGTTCAGTAGCCAACGGC |
|  | CpPP2C65-pMDC32-F |  | GGGGACAAGTTTGTACAAAAAAGCAGGCTATGGGAACCTGTTGT |
|  | CpPP2C65-pMDC32-R |  | GGGGACCACTTTGTACAAGAAAGCTGGGTTCAAAACACATGAGA |
| VIGS | CpCML15-pTRV2-F |  | TACCGAATTCTCTAGAATGGGAGAGTTCGAGGAGCAGGATCAGCTG |
|  | CpCML15-pTRV2-R |  | GGGACATGCCCGGGCGAGAAGTGAAAGCCAAA |
|  | CpPP2C46-pTRV2-F |  | TACCGAATTCTCTAGAAACAATTTACTTGAG |
|  | CpPP2C46-pTRV2-R |  | CTTCGGGACATGCCCGGGATCGTGGAAATGCCT |
|  | CpPP2C65-pTRV2-F |  | TACCGAATTCTCTAGAAGGGGATCAATCAAG |
|  | CpPP2C65-pTRV2-R |  | CTTCGGGACATGCCCGGGATCAATCCTTGAACA |
