# Supplementary material for: Calmodulin‐like protein CML15 interacts with PP2C46/65 to regulate papaya fruit ripening via integrating calcium, ABA and ethylene signals
Source: Plant Biotechnol J. 2024 Feb 6;22(6):1703–23. doi: 10.1111/pbi.14297 (PMC11123395; doi:10.1111/pbi.14297)
Supplement: Supplementary file 5 — Figure S1 The expression profile of SlCML15 during fruit development (a) and under ACC treatment (b). The RNA‐seq data for SlCML15 were obtained from the TomExpress database (http://tomexpress.toulouse.inra.fr). DPA: days post‐anthesis. Figure S2 Y2H assay showed that AtCaM1 do not interact with CpPP2C46/65. Figure S3 Sequences analysis of CpPP2C46/65. Figure S4 Heatmap of the expression profiles of CpPP2Cs during fruit ripening. Figure S5 Effect of 1‐MCP and ethephon treatments on the transcription of genes involved in ethylene and ABA synthesis and signal pathway. Figure S6 The transient overexpression or silencing of CpCML15 in papaya fruit altered the expression of genes related to ethylene and fruit softening. Figure S7 The transient overexpression or silencing of CpPP2C46 alters the expression profiles of genes associated with ethylene and fruit softening during fruit ripening. Figure S8 The transient overexpression or silencing of CpPP2C65 alters the expression profiles of genes associated with ethylene and fruit softening during fruit ripening. Figure S9 Samples correlation map and volcano map show the distribution of significant DEGs. Figure S10 Overexpression of CpCML15 alters the transcript profiles of tomato fruit. Figure S11 Comparison of the DEGs of the WT and CpCML15‐OE lines in GO classification. Figure S12 The heterologous overexpression of CpCML15 and CpPP2C46 in tomato altered plant sensitivity to ABA‐mediated inhibition of seeds germination. Figure S13 The heterologous overexpression of CpCML15 and CpPP2C46 in tomato altered plant sensitivity to ABA‐mediated inhibition of primary root growth. Figure S14 The triple‐response experiment of CpCML15‐OE and WT lines. Figure S15 Y2H was used to verify the interactions of CpCML15 (a), CpPP2C46 (b) and CpPP2C65 (c) with proteins in ethylene synthesis and ABA signal transduction pathway. SD medium for yeast growth was lacking Trp, His, Leu and Ade. Blue plaques display the interaction of protein staining w [file PBI-22-1703-s005.pdf]

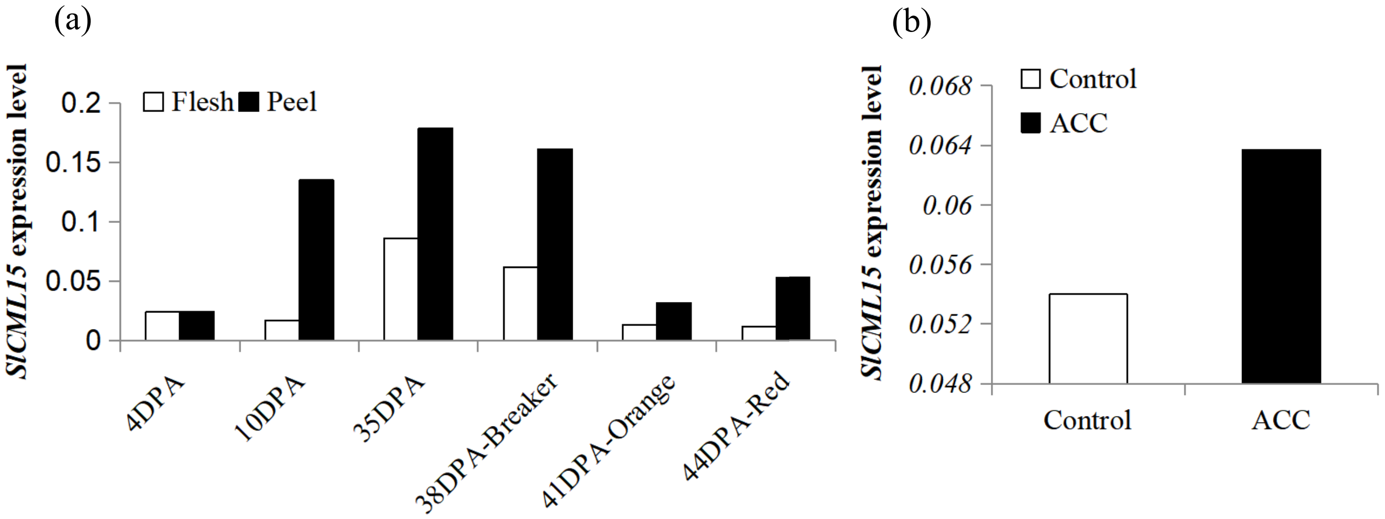

Figure S1. The expression profile of *SlCML15* during fruit development (a) and under ACC treatment (b). The RNA-seq data for *SlCML15* were obtained from the TomExpress database (http://tomexpress.toulouse.inra.fr). DPA: days post-anthesis.

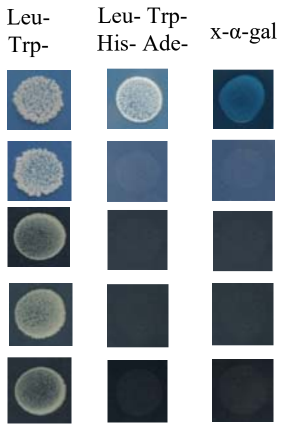

BD	AD	Interaction
p53	pGADT7-T	+
Lamin	pGADT7-T	-
AtCaM1	pGADT7-T	-
AtCaM1	CpPP2C46	-
AtCaM1	CpPP2C65	-

Figure S2. Y2H assay showed that AtCaM1 do not interact with CpPP2C46/65. AtCaM1 was introduced into the pGBKT7 vector. The AtCaM1 recombinant protein served as a bait protein. CpPP2C46/65 were the prey proteins. The Y2H yeast strains were co-transformed with AtCaM1 + CpPP2C46/65. pGADT7-T + pGBKT7-53 or pGADT7-T + pGBKT7-lam were set as the positive and negative controls, respectively. SD medium for yeast growth was lacking Trp, His, Leu, and Ade. Blue plaques display the interaction of protein staining with X-α-gal.

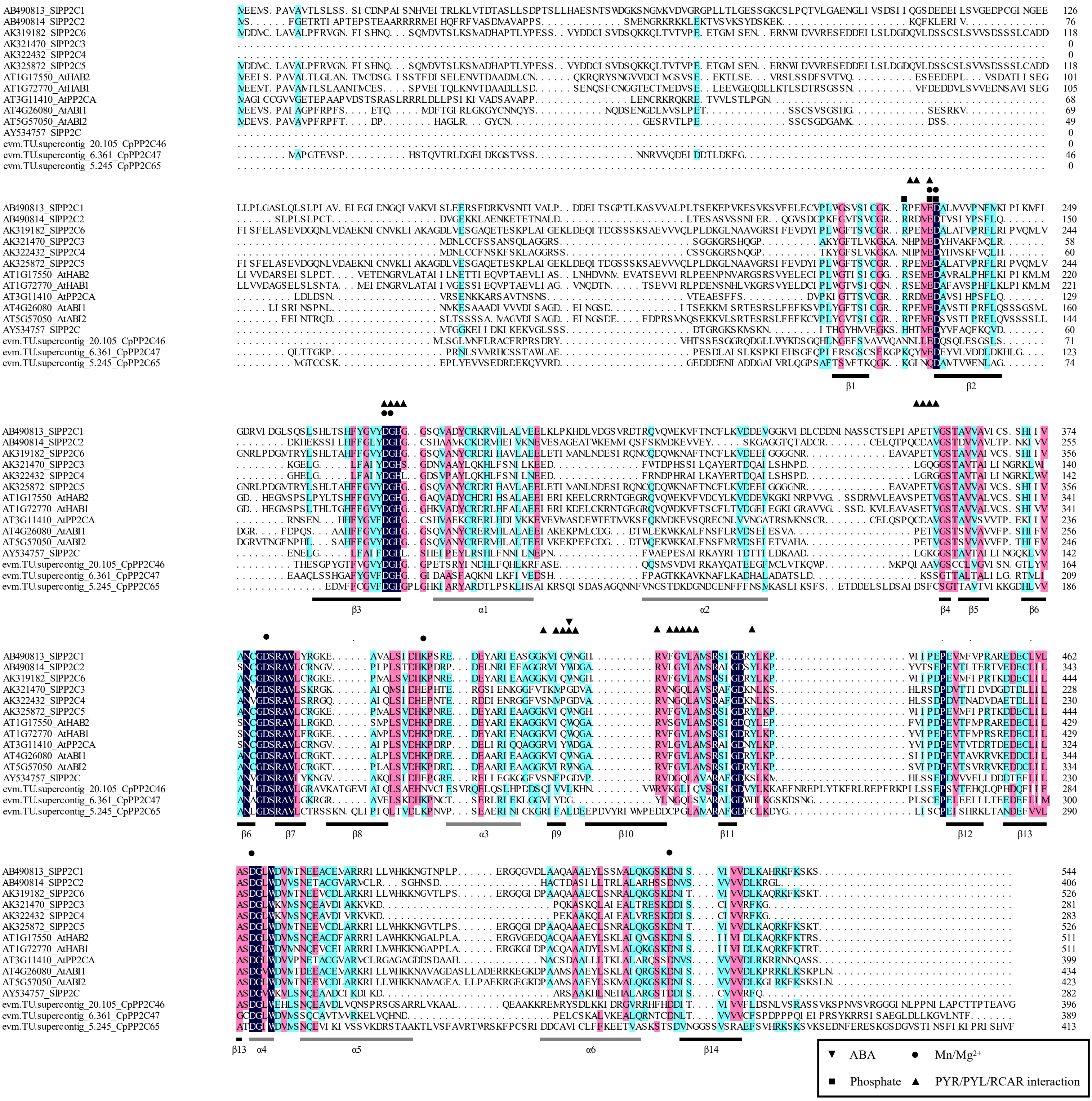

Figure S3. Sequences analysis of CpPP2C46/65.

Amino acid sequences alignment of CpPP2C46/65 and PP2Cs from *Arabidopsis* and tomato, with secondary structure elements of ABI1 below the sequences. The alignment was performed by DNAMAN. The conserved residues were marked with coloring and shadowing, and alignment gaps are indicated by dashes. Residues interacted with ABA, PYLs and Mn/Mg ions and phosphatase sites are marked with different shape in lower right corner. Functional residues and domains and HAB1 secondary structure elements are marked according to work of Sun et al. (2011).

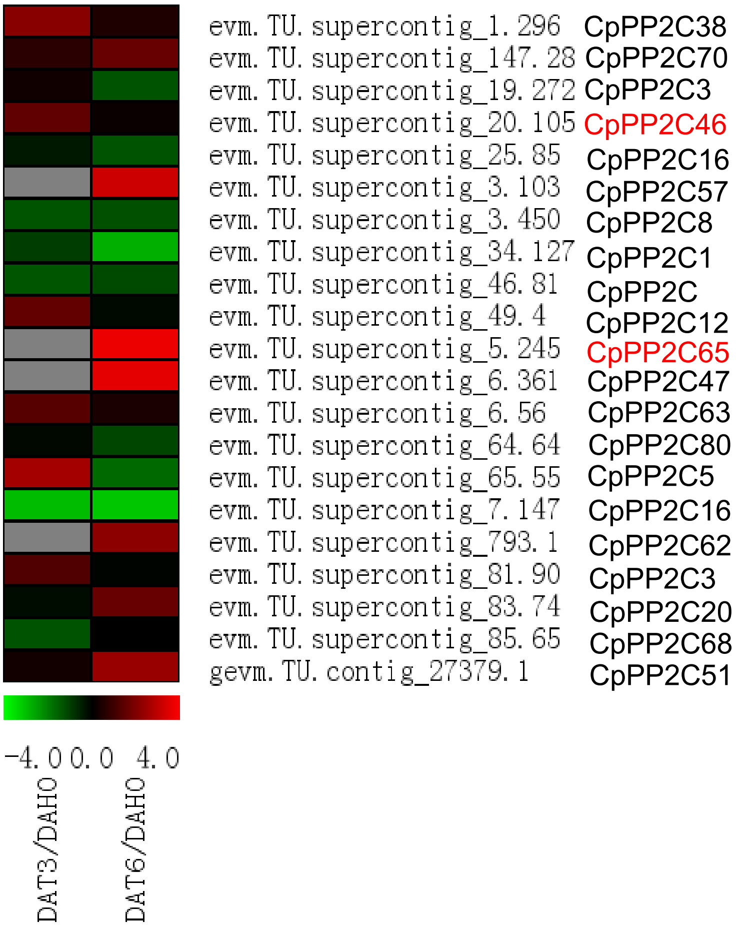

Figure S4. Heatmap showing the expression profiles of *CpPP2Cs* during fruit ripening. Expression data were extracted from our previous RNA-seq analysis, samples after ethephon treatment and during the ripening period. The heatmap was drawn using MeV software.

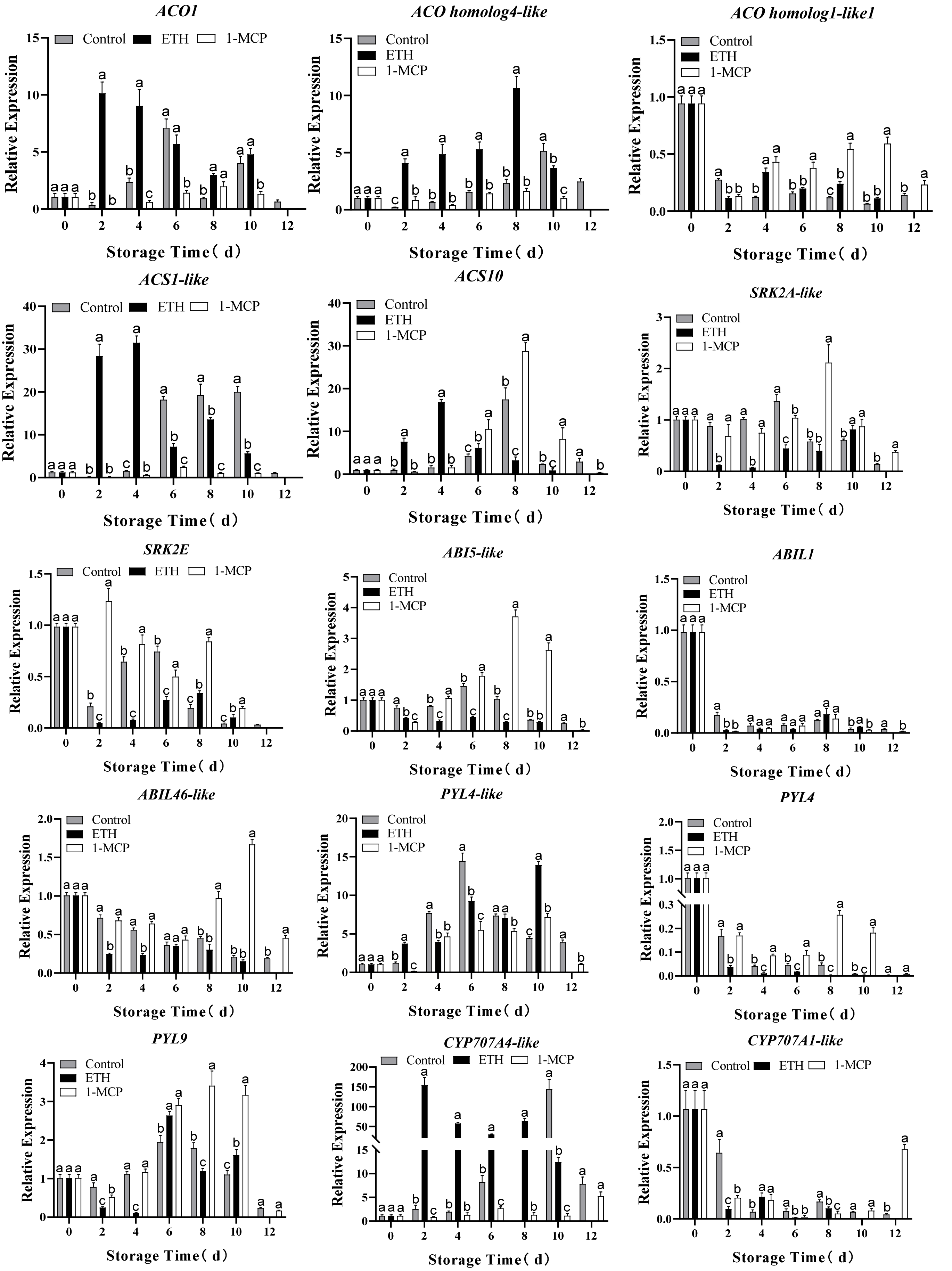

Figure S5. The effect of 1-MCP and ethephon treatments on the transcription of genes involved in ethylene and ABA synthesis and signal pathway. The expression analysis was conducted by RT-qPCR. Expression data at different sampling days are relative to 0 d (untreated fruits), which was set as 1. The *CpTBP1* and *CpTBP2* were used as reference genes. Data are presented as the means ±SD of three biological replicates. The different letters between treatments indicate statistical differences at the 5% level.

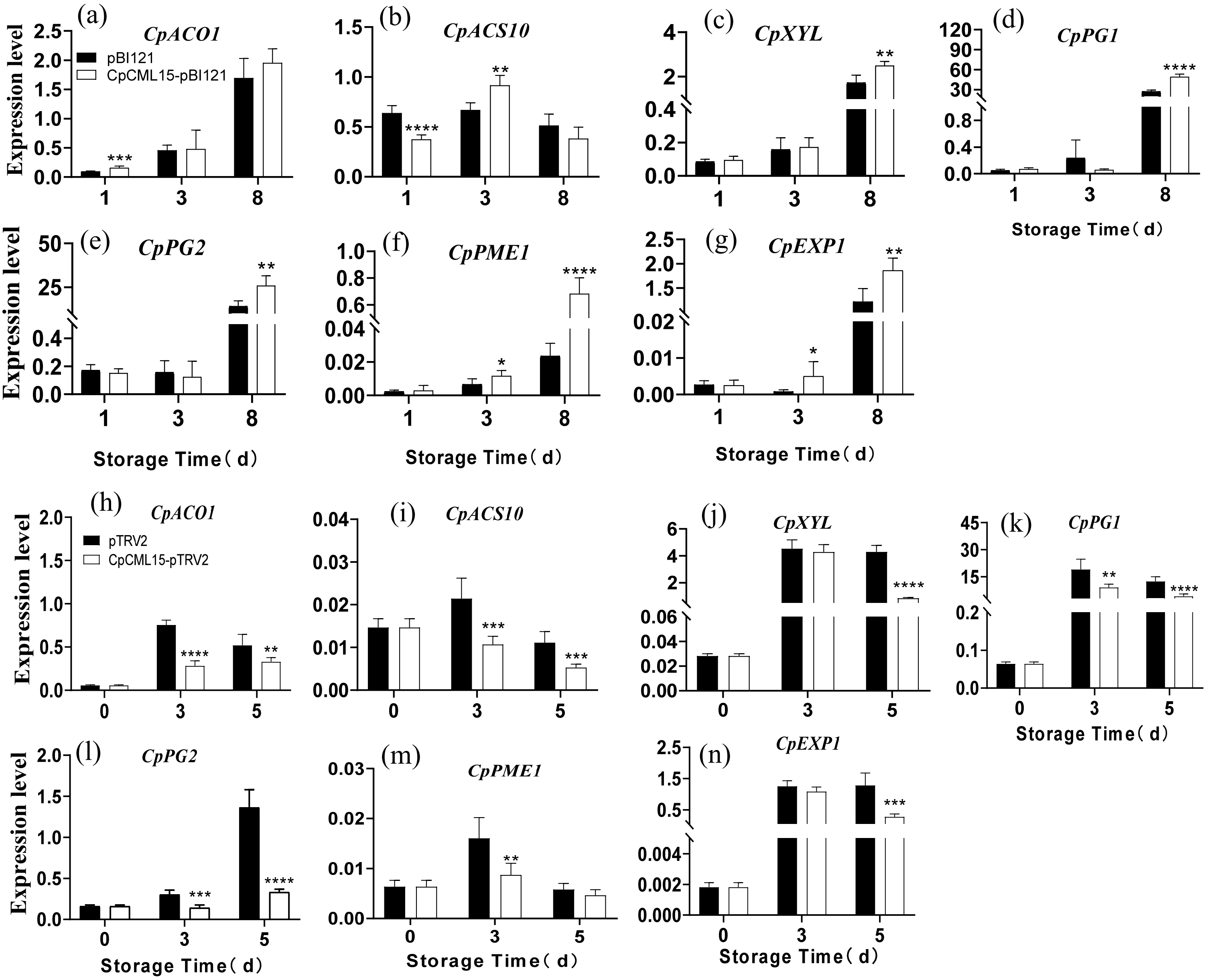

Figure S6. The transient overexpression or silencing of *CpCML15* in papaya fruit altered the expression of genes related to ethylene and fruit softening. (a-g) Transcripts of genes related to ethylene and fruit softening were determined using RT-qPCR in transient overexpression of *CpCML15* fruit around the injection sites. (h-n) Transcripts of genes related to ethylene and fruit softening were determined using RT-qPCR in transient silencing of *CpCML15* fruit around the injection sites. The *CpTBP1* and *CpTBP2* were used as reference genes. The gene expression level was related to the reference genes (geometric mean of *CpTBP1* and *CpTBP2*), which calculated by $2^{-\Delta Ct}$ formula. Data are presented as the means ± SD of three biological replicates. Asterisks (*, **, *** and ****) present significant differences at the $P < 0.05$, $P < 0.01$, $P < 0.001$ and $P < 0.0001$ level.

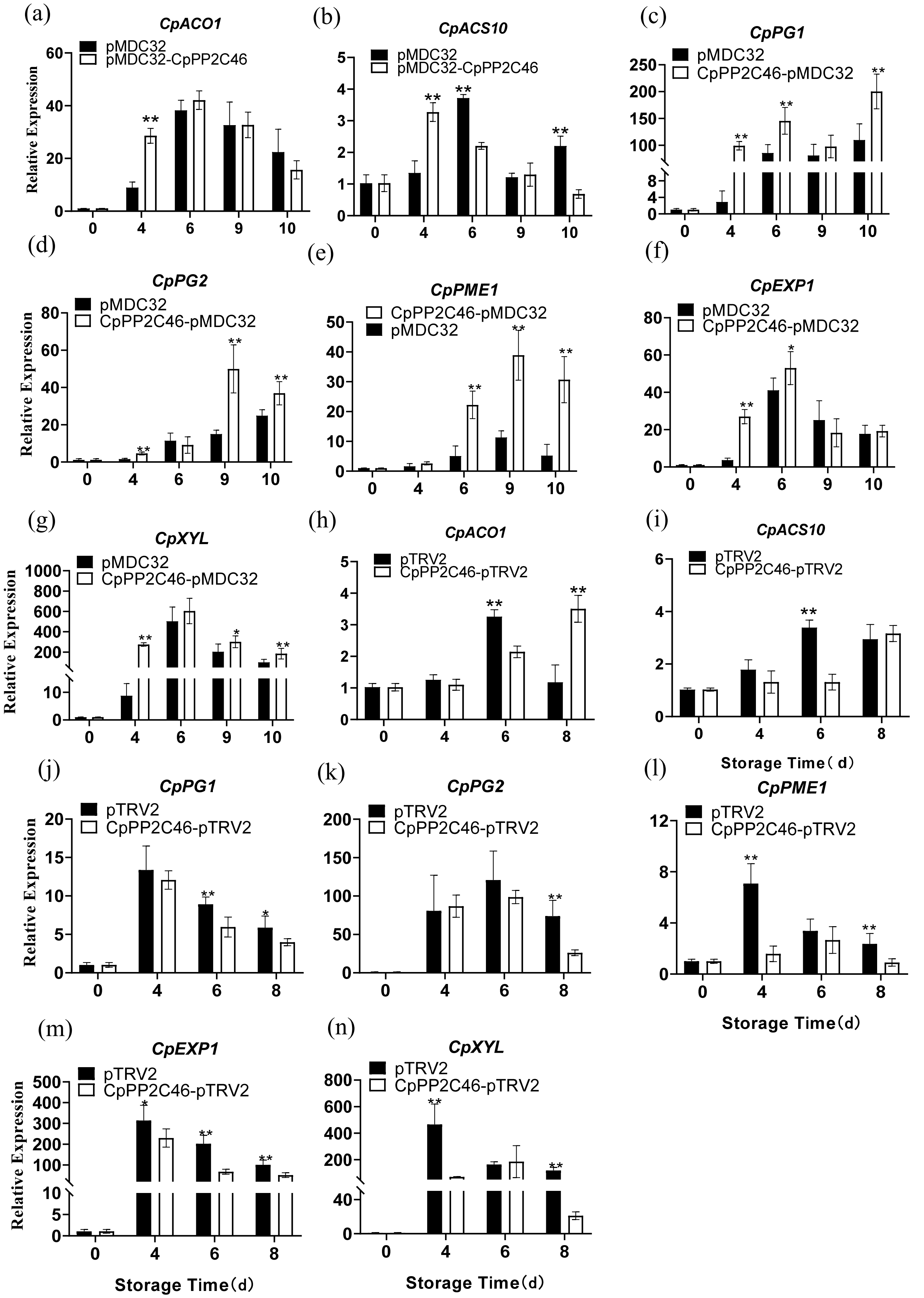

Figure S7. The transient overexpression silencing of *CpPP2C46* alter the expression profiles of genes associated with ethylene and fruit softening during fruit ripening process. (a-g) Transcripts of genes related to fruit softening were determined using RT-qPCR in transient over-expression fruit around the injection sites. (h-n) Expression profiles of genes related to ethylene and fruit softening were determined using RT-qPCR in transient silencing fruit around the injection sites. Expression data on different sampling days were relative to 0 d (untreated fruit), which was set as 1. The *CpTBP1* and *CpTBP2* were used as reference genes. Data are presented as the means ± SD of three biological replicates. *, ** indicate statistical differences at the 0.05 and 0.01 level.

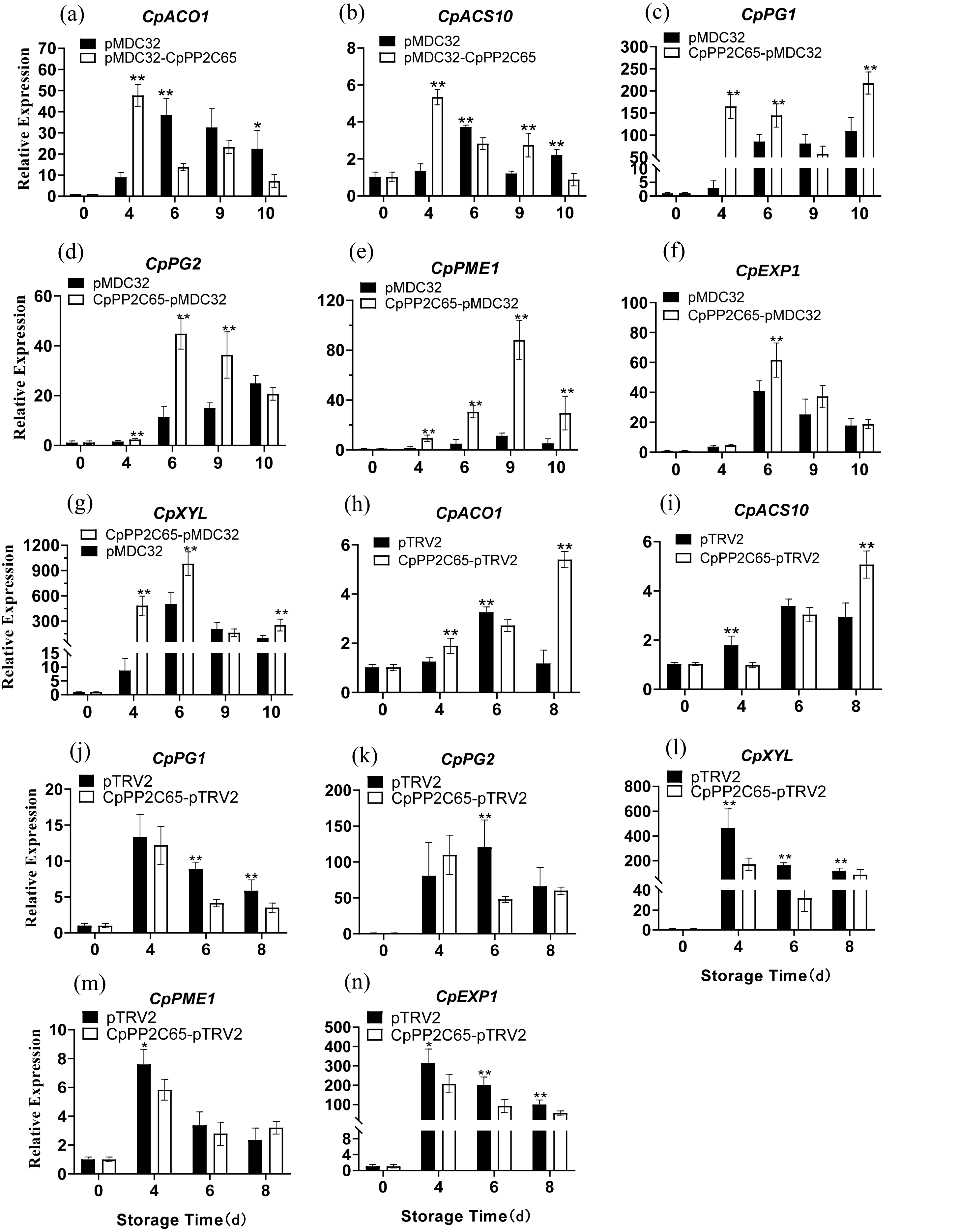

Figure S8. The transient over-expression silencing of *CpPP2C65* alter the expression profiles of genes associated with ethylene and fruit softening during fruit ripening process. (a-g) Transcripts of genes related to fruit softening were determined using RT-qPCR in transient over-expression fruit around the injection sites. (h-n) Expression profiles of genes related to ethylene and fruit softening were determined using RT-qPCR in transient silencing fruit around the injection sites. Expression data on different sampling days were relative to 0 d (untreated fruit), which was set as 1. The *CpTBP1* and *CpTBP2* were used as reference genes. Data are presented as the means ± SD of three biological replicates. *, ** indicate statistical differences at the 0.05 and 0.01 level.

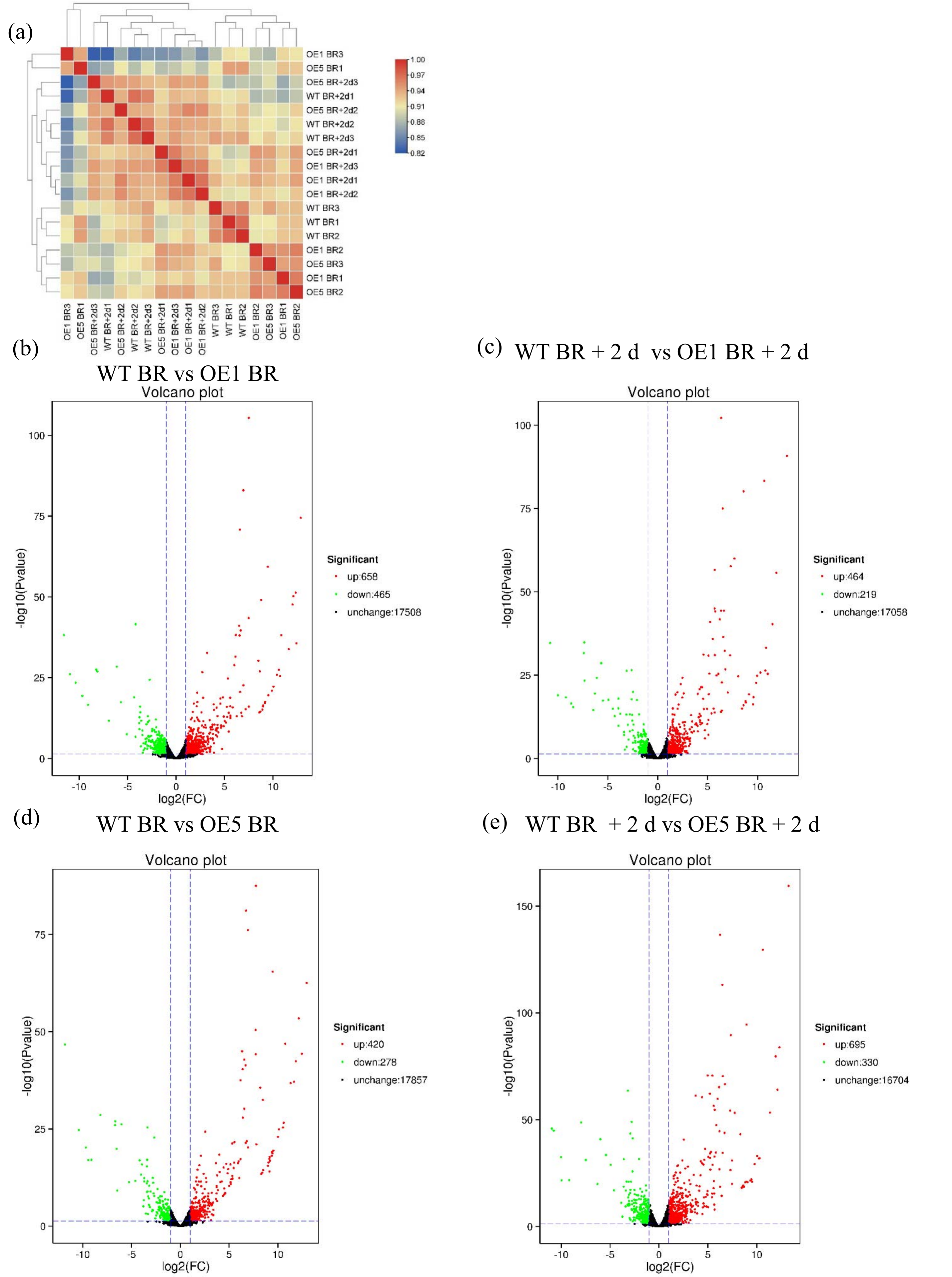

Figure S9. Samples correlation map and volcano map shows distribution of significantly DEGs. Samples correlation map (a); (b-c), volcano map analysis comparing the WT and *CpCML15*-OE1 line at BR (b) and BR+2 d (c) stages. (d-e), volcano map analysis comparing the WT and *CpCML15*-OE5 line at BR (d) and BR+2 d (e) stages. The up- or down-regulated DEGs were indicated by red or green dot in the volcano map, with the numbers.

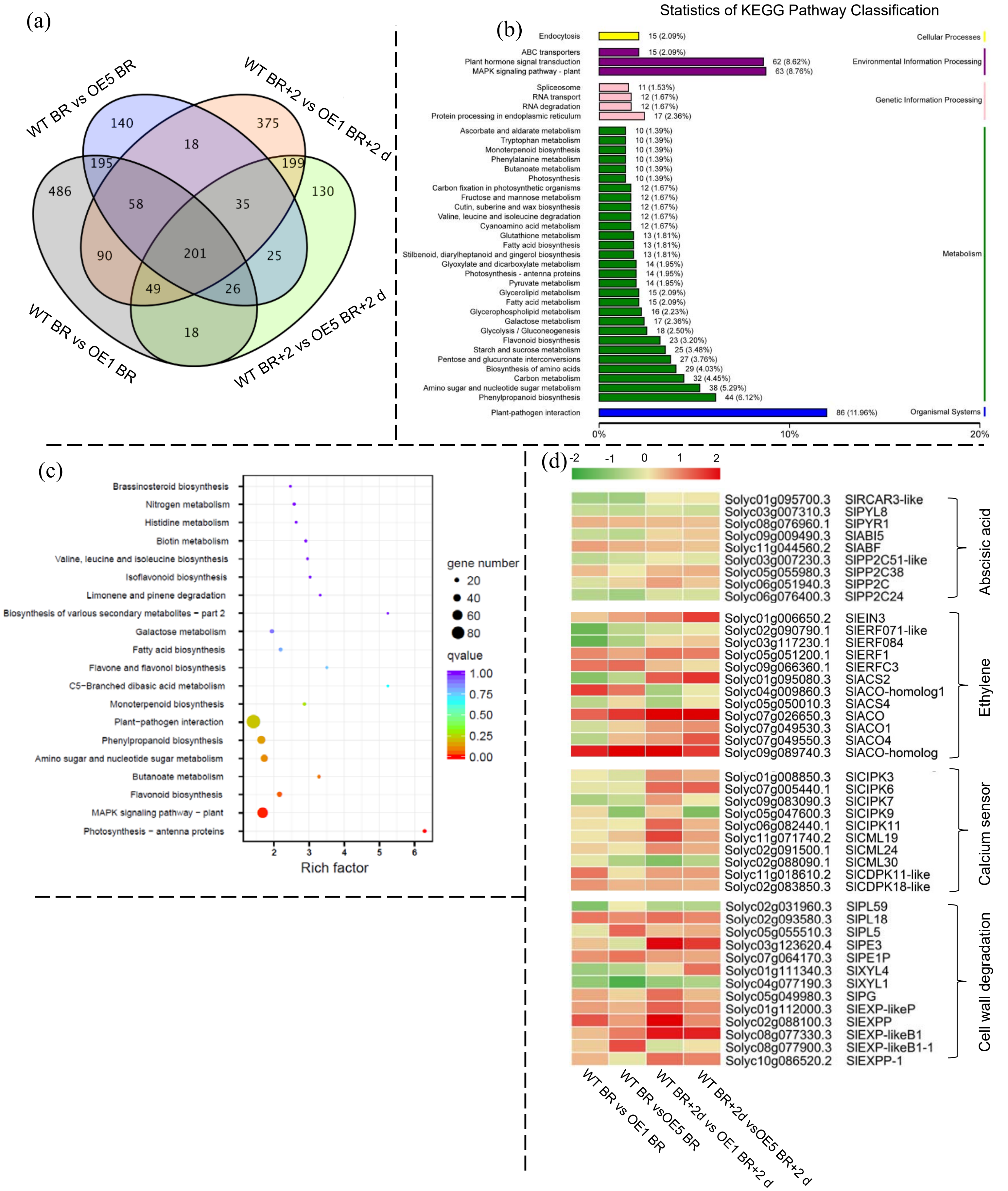

(a)
WT BR vs OE5 BR
WT BR+2 vs OE1 BR+2 d
WT BR vs OE1 BR
WT BR+2 vs OE5 BR+2 d
140
375
18
195
199
486
58
35
130
90
201
25
49
26
18
(b)
Statistics of KEGG Pathway Classification
Endocytosis 15 (2.09%)
ABC transporters 15 (2.09%)
Plant hormone signal transduction 62 (8.62%)
MAPK signaling pathway - plant 63 (8.76%)
Spliceosome 11 (1.53%)
RNA transport 12 (1.67%)
RNA degradation 12 (1.67%)
Protein processing in endoplasmic reticulum 17 (2.36%)
Ascorbate and aldarate metabolism 10 (1.39%)
Tryptophan metabolism 10 (1.39%)
Monoterpenoid biosynthesis 10 (1.39%)
Phenylalanine metabolism 10 (1.39%)
Butanoate metabolism 10 (1.39%)
Photosynthesis 10 (1.39%)
Carbon fixation in photosynthetic organisms 12 (1.67%)
Fructose and mannose metabolism 12 (1.67%)
Cutin, suberine and wax biosynthesis 12 (1.67%)
Valine, leucine and isoleucine degradation 12 (1.67%)
Cyanoamino acid metabolism 12 (1.67%)
Glutathione metabolism 13 (1.81%)
Fatty acid biosynthesis 13 (1.81%)
Stilbenoid, diarylheptanoid and gingerol biosynthesis 13 (1.81%)
Glyoxylate and dicarboxylate metabolism 14 (1.95%)
Photosynthesis - antenna proteins 14 (1.95%)
Pyruvate metabolism 14 (1.95%)
Glycerolipid metabolism 15 (2.09%)
Fatty acid metabolism 15 (2.09%)
Glycerophospholipid metabolism 16 (2.23%)
Galactose metabolism 17 (2.36%)
Glycolysis / Gluconeogenesis 18 (2.50%)
Flavonoid biosynthesis 23 (3.20%)
Starch and sucrose metabolism 25 (3.48%)
Pentose and glucuronate interconversions 27 (3.76%)
Biosynthesis of amino acids 29 (4.03%)
Carbon metabolism 32 (4.45%)
Amino sugar and nucleotide sugar metabolism 38 (5.29%)
Phenylpropanoid biosynthesis 44 (6.12%)
Plant-pathogen interaction 86 (11.96%)
Cellular Processes
Environmental Information Processing
Genetic Information Processing
Metabolism
Organismal Systems
0%
10%
20%
(c)
Brassinosteroid biosynthesis
Nitrogen metabolism
Histidine metabolism
Biotin metabolism
Valine, leucine and isoleucine biosynthesis
Isoflavonoid biosynthesis
Limonene and pinene degradation
Biosynthesis of various secondary metabolites − part 2
Galactose metabolism
Fatty acid biosynthesis
Flavone and flavonol biosynthesis
C5−Branched dibasic acid metabolism
Monoterpenoid biosynthesis
Plant−pathogen interaction
Phenylpropanoid biosynthesis
Amino sugar and nucleotide sugar metabolism
Butanoate metabolism
Flavonoid biosynthesis
MAPK signaling pathway − plant
Photosynthesis − antenna proteins
2
3
4
5
6
Rich factor
gene number
20
40
60
80
qvalue
1.00
0.75
0.50
0.25
0.00
(d)
-2
-1
0
1
2
Solyc01g095700.3 SlRCAR3-like
Solyc03g007310.3 SlPYL8
Solyc08g076960.1 SlPYR1
Solyc09g009490.3 SlABI5
Solyc11g044560.2 SlABF
Solyc03g007230.3 SlPP2C51-like
Solyc05g055980.3 SlPP2C38
Solyc06g051940.3 SlPP2C
Solyc06g076400.3 SlPP2C24
Abscisic acid
Solyc01g006650.2 SlEIN3
Solyc02g090790.1 SlERF071-like
Solyc03g117230.1 SlERF084
Solyc05g051200.1 SlERF1
Solyc09g066360.1 SlERFC3
Solyc01g095080.3 SlACS2
Solyc04g009860.3 SlACO-homolog1
Solyc05g050010.3 SlACS4
Solyc07g026650.3 SlACO
Solyc07g049530.3 SlACO1
Solyc07g049550.3 SlACO4
Solyc09g089740.3 SlACO-homolog
Ethylene
Solyc01g008850.3 SlCIPK3
Solyc07g005440.1 SlCIPK6
Solyc09g083090.3 SlCIPK7
Solyc05g047600.3 SlCIPK9
Solyc06g082440.1 SlCIPK11
Solyc11g071740.2 SlCML19
Solyc02g091500.1 SlCML24
Solyc02g088090.1 SlCML30
Solyc11g018610.2 SlCDPK11-like
Solyc02g083850.3 SlCDPK18-like
Calcium sensor
Solyc02g031960.3 SlPL59
Solyc02g093580.3 SlPL18
Solyc05g055510.3 SlPL5
Solyc03g123620.4 SlPE3
Solyc07g064170.3 SlPE1P
Solyc01g111340.3 SlXYL4
Solyc04g077190.3 SlXYL1
Solyc05g049980.3 SlPG
Solyc01g112000.3 SlEXP-likeP
Solyc02g088100.3 SlEXPP
Solyc08g077330.3 SlEXP-likeB1
Solyc08g077900.3 SlEXP-likeB1-1
Solyc10g086520.2 SlEXPP-1
Cell wall degradation
WT BR vs OE1 BR
WT BR vsOE5 BR
WT BR+2d vs OE1 BR+2 d
WT BR+2d vsOE5 BR+2 d

Figure S10. Overexpression of *CpCML15* alters the transcript profiles of tomato fruit.

(a) Venn diagram analysis of the wild-type (WT) and *CpCML15*-OE lines at the BR and BR + 2 d stages. (b) KEGG classification of DEGs comparison of the WT and *CpCML15*-OE lines at the BR and BR + 2 d stages. (c) Top 20 KEGG enrichment pathways of the comparison between WT and *CpCML15*-OE lines at the BR and BR + 2 d stages. (d) The expression profiles of selected key genes related to the abscisic acid, calcium, ethylene, and cell wall pathways between the comparison of WT and *CpCML15*-OE lines at the BR and BR + 2 d stages in a heatmap. Software package (http://bioinformatics.psb.ugent.be/webtools/) was used for the Venn diagram and KEGG analysis. The heatmap was drawn using MeV software. BR: break stage.

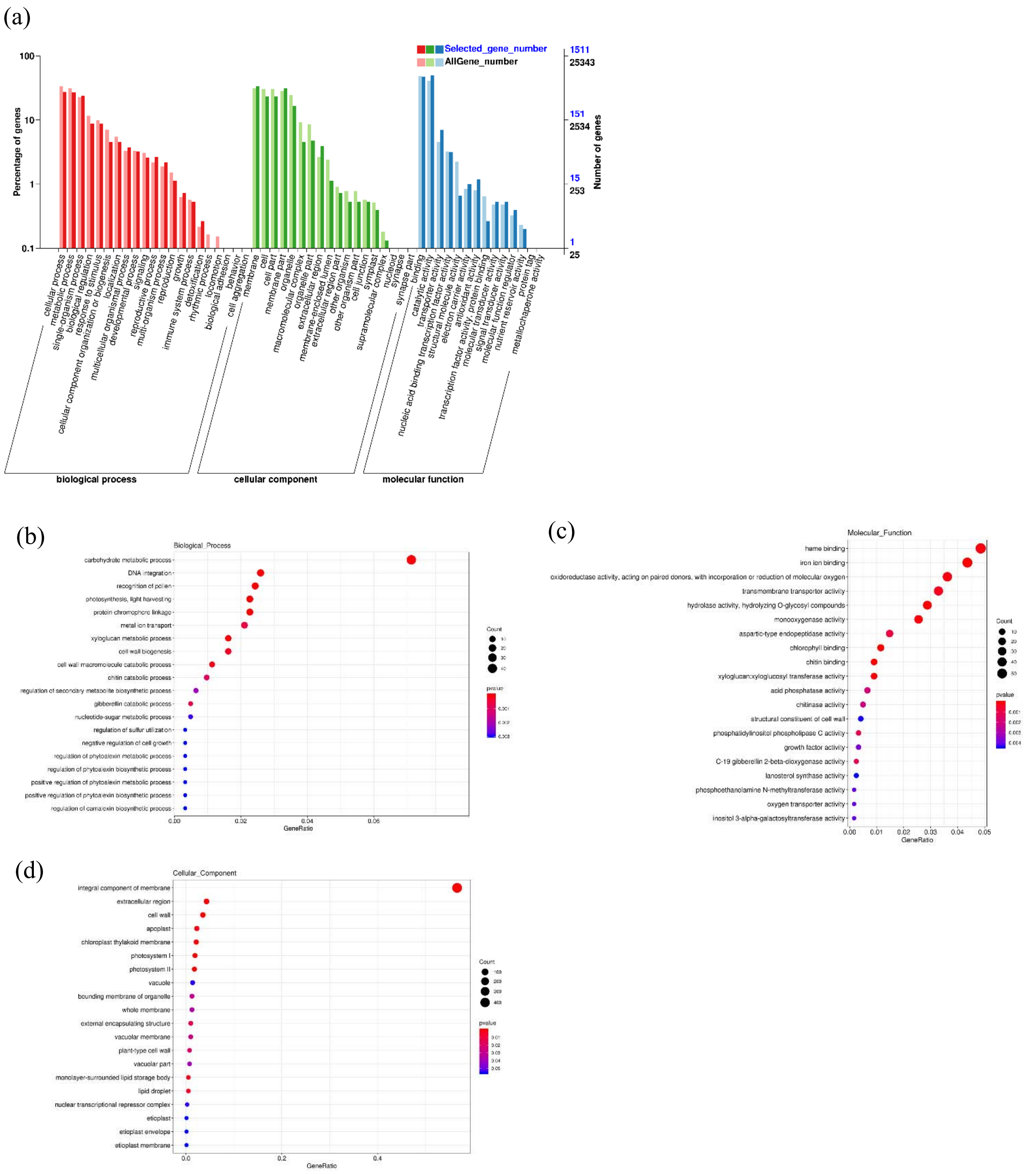

Figure S11. Comparison of the DEGs of the WT and *CpCML15*-OE lines in GO classification.

(a) GO classification of DEGs comparison of the WT and *CpCML15*-OE lines at BR and BR +2 d stages. (b) TOP 20 GO enrichment pathways comparing the WT and *CpCML15*-OE lines at BR and BR +2 d stages in biological process. (c) TOP 20 GO enrichment pathways comparing the WT and *CpCML15*-OE lines at BR and BR +2 d stages in molecular function. (d) TOP 20 GO enrichment pathways comparing the WT and *CpCML15*-OE lines at BR and BR +2 d stages in cellular component.

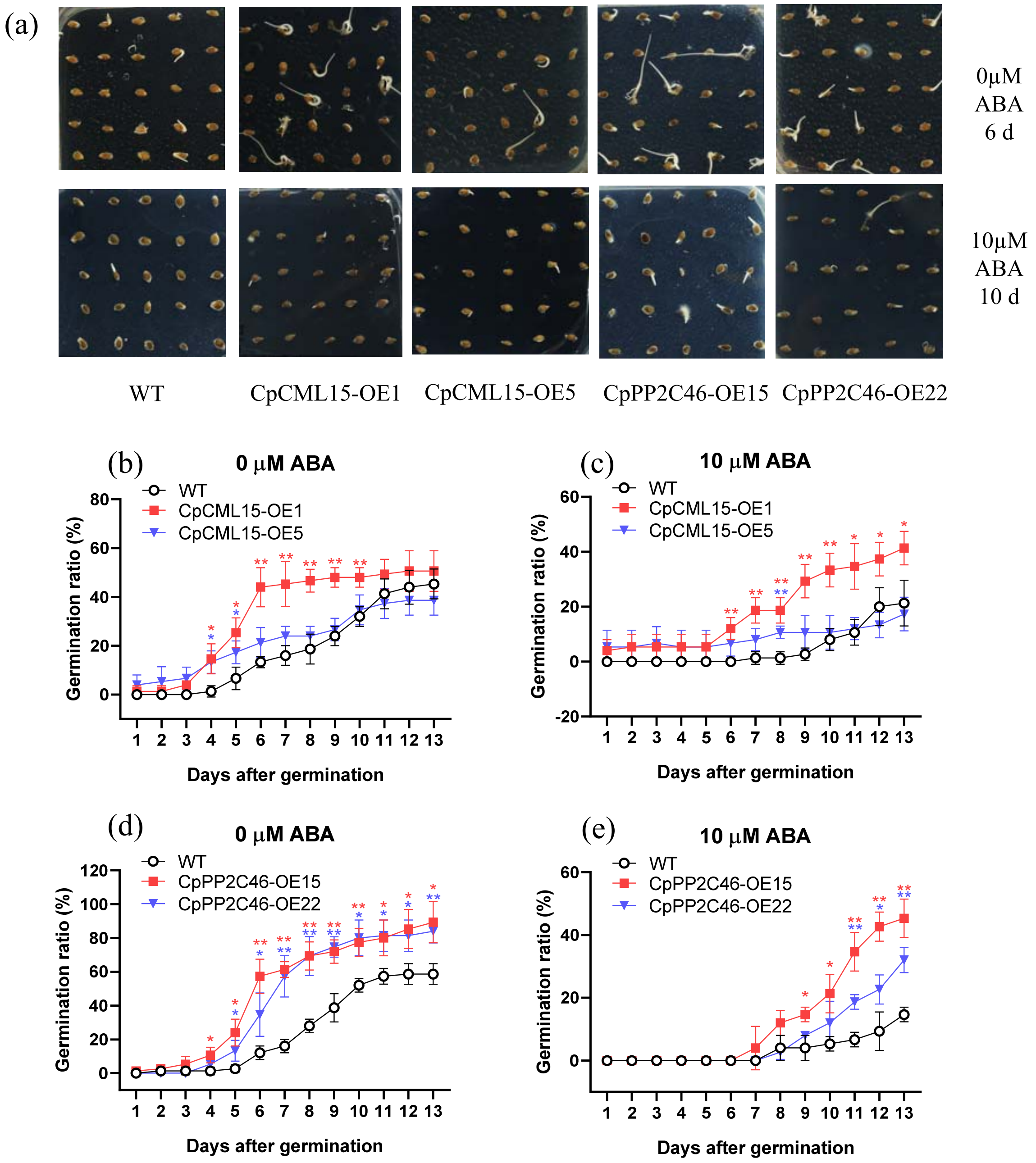

Figure S12. The heterologous overexprssion of *CpCML15* and *CpPP2C46* in tomato altered plant sensitivity to ABA-mediated inhibition of seeds germination. (a) Images of tomato seed germination of the WT, *CpCML15*-OE lines and *CpPP2C46*-OE lines, with or without ABA. (b-e) Germination rate of tomato seeds in WT, *CpCML15*-OE and *Cp*PP2C46-OE lines. Approximately 25 seeds of WT, *CpCML15*-OE1, *CpCML15*-OE5, *CpPP2C46*-OE15 and *CpPP2C46*-OE22 lines (three independent experiments) were sown on MS medium with or without 10 μM ABA. Data are presented as the means ± SD of three biological replicates. Asterisks (* and **) present significant differences at the $P < 0.05$ and $P < 0.01$ level.

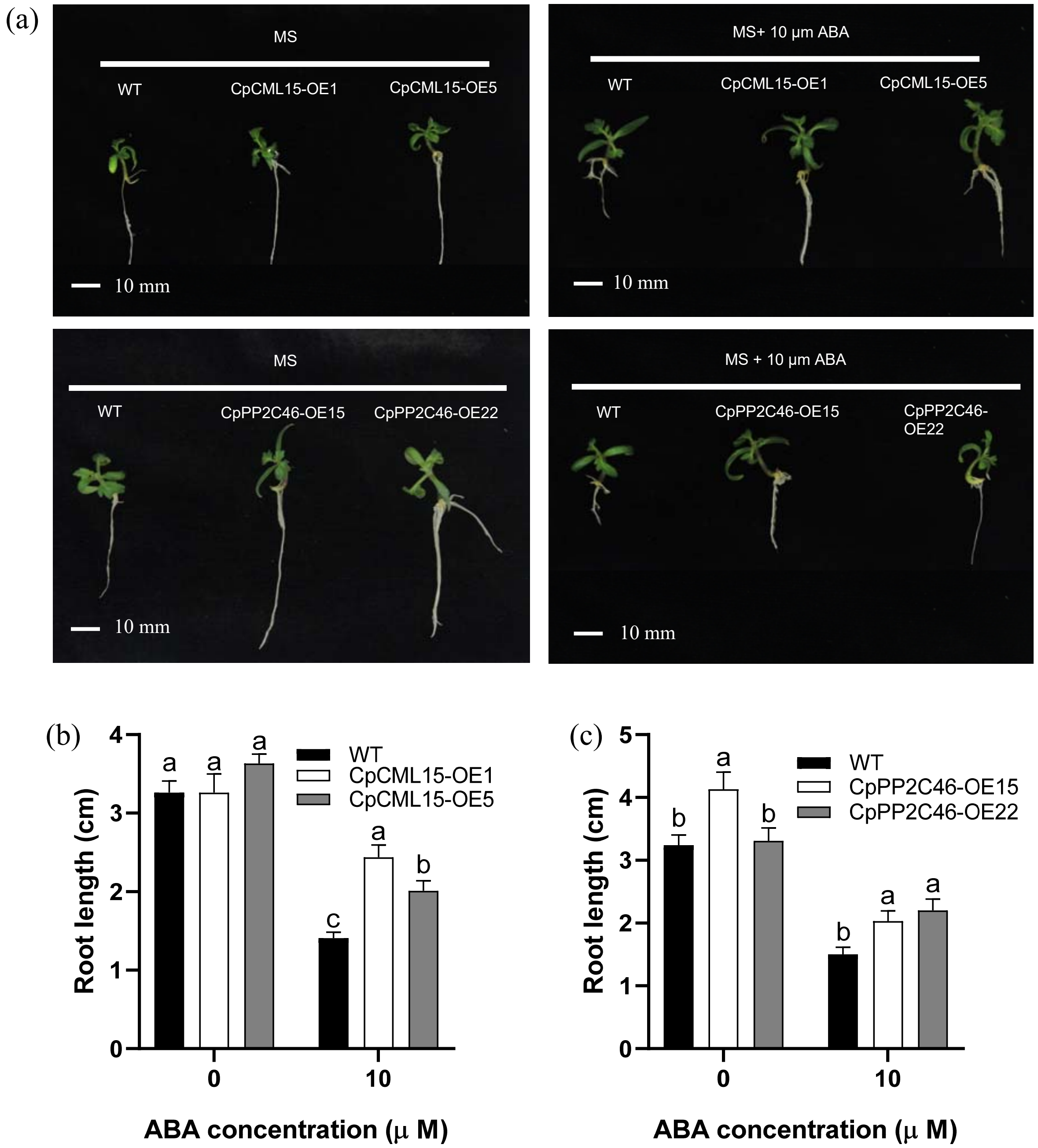

Figure S13. The heterologous overexpression of *CpCML15* and *CpPP2C46* in tomato altered plant sensitivity to ABA-mediated inhibition of primary root growth. (a) Representative images of tomato roots of WT and different transgenic lines. (b, c) Primary root length of *CpCML15*-OE and *CpPP2C46*-OE lines. Primary roots grown for 5 d on MS medium with or without 10 μM ABA. Data are presented as the means ± SD of three biological replicates. The different letters indicate statistical differences between treatments at the 5% level.

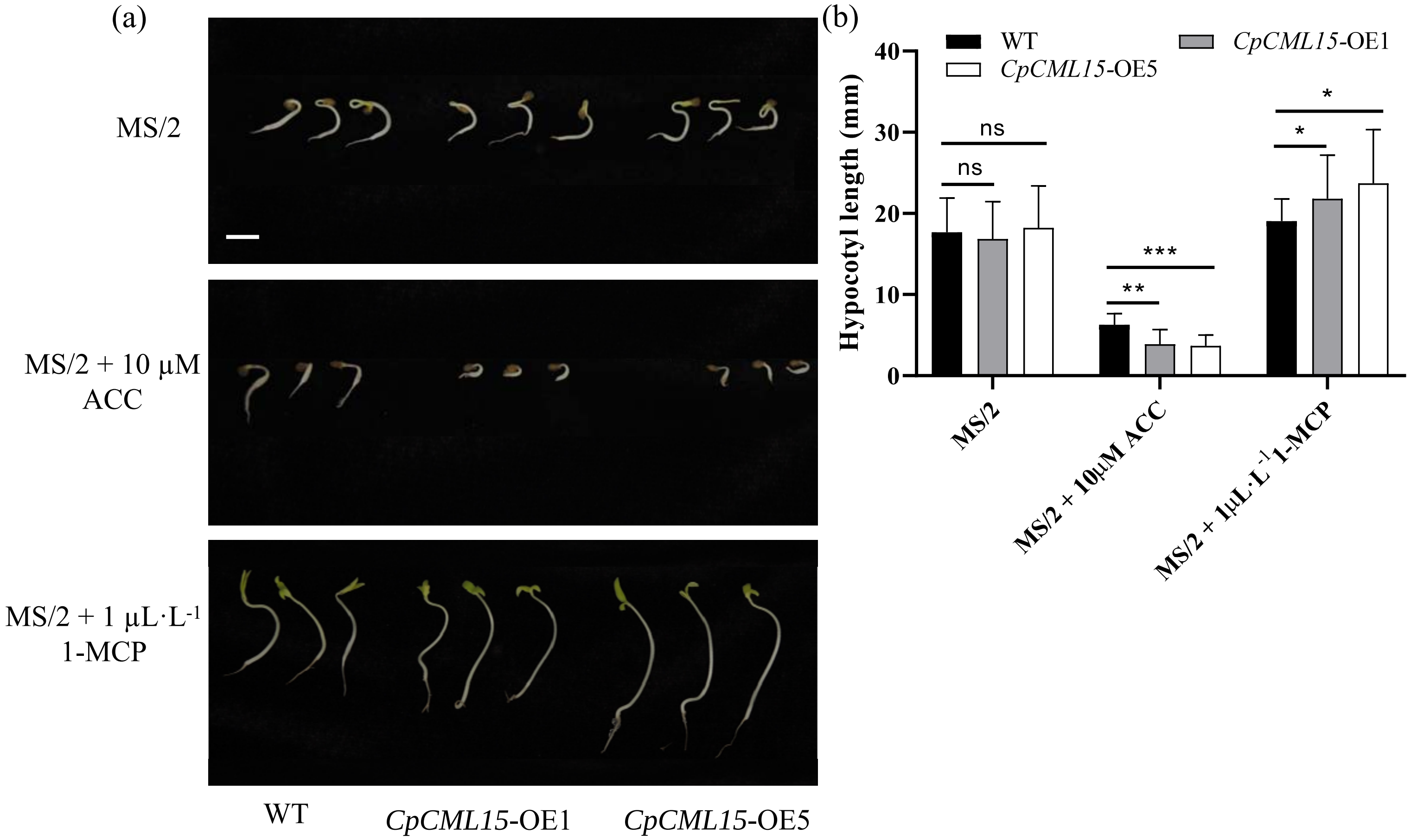

Figure S14. The triple-response experiment of *CpCML15*-OE and WT lines.

The seedlings of *CpCML15*-OE1/5 and WT lines were transferred to MS/2 medium with 10 µM ACC or without ACC, or treated with 1 µL L^{-1} of 1-MCP for 40 h in darkness. The seedling triple-response was scored by assessing hypocotyl length. The length of hypocotyl was measured using image J software (https://imagej.nih.gov/ij/), and at least 50 seedlings were measured for each line. Asterisks (*, ** and ***) present significant differences at the $P < 0.05$, $P < 0.01$ and $P < 0.001$ level. scar bar=10 mm.

(a)

Leu-Trp- | Leu-Trp-His- Ade- | X-α-gal

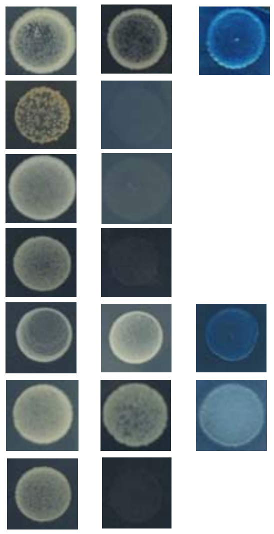

BD	AD	interaction
p53	pGADT7-T	+
Lamin	pGADT7-T	-
CpACO1	CpCML15	-
CpACS1 -like	CpCML15	-
CpACO-h1-like2	CpCML15	self activated
CpACO-h1-like2	pGADT7-T	self activated
CpACO-h4-like	CpCML15	-

(b)

Leu-Trp- | Leu-Trp-His- Ade- | X-α-gal

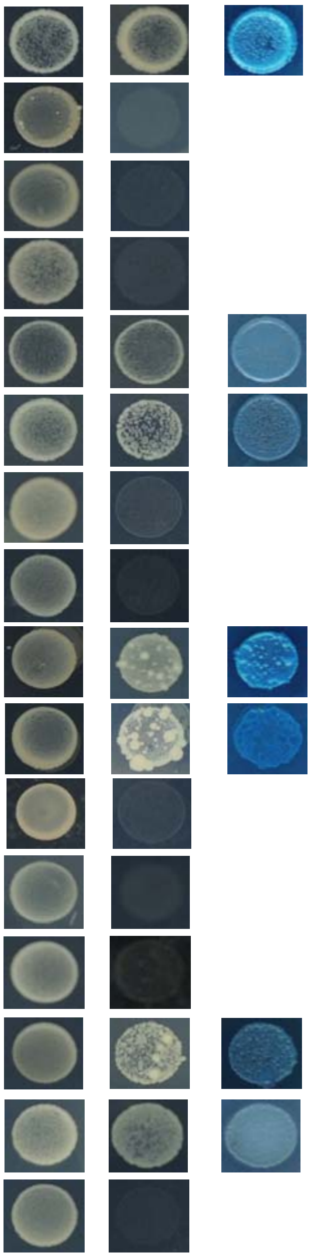

BD	AD	interaction
p53	pGADT7-T	+
Lamin	pGADT7-T	-
CpPYL4	CpPP2C46	-
CpPYL9	CpPP2C46	-
CpSRK2E	CpPP2C46	self activated
CpSRK2E	pGADT7-T	self activated
CpSRK2A	CpPP2C46	-
CpSRK2A -like	CpPP2C46	-
CpACO1	CpPP2C46	+
CpPP2C46	CpACO1	+
CpACO1	pGADT7-T	-
CpACS1-like	CpPP2C46	-
CpACO-h1-like1	CpPP2C46	-
CpACO-h1-like2	CpPP2C46	self activated
CpACO-h1-like2	pGADT7-T	self activated
CpACO-h4-like	CpPP2C46	-

(c)

Leu-Trp- | Leu-Trp-His- Ade- | X-α-gal

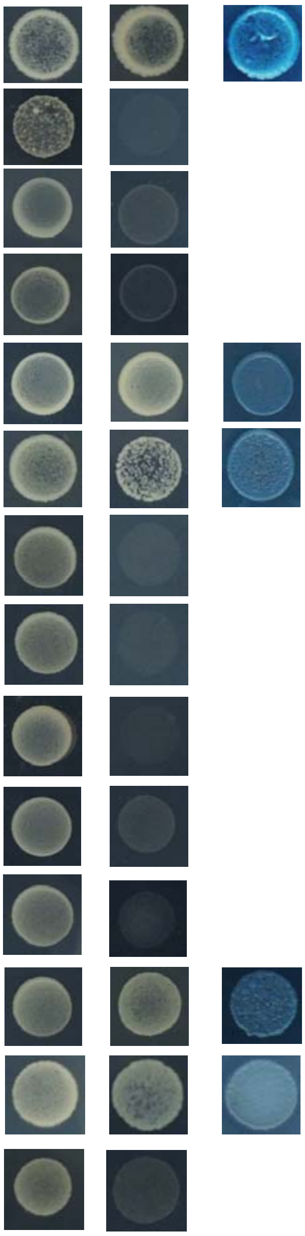

BD	AD	interaction
p53	pGADT7-T	+
Lamin	pGADT7-T	-
CpPYL4	CpPP2C65	-
CpPYL9	CpPP2C65	-
CpSRK2E	CpPP2C65	self activated
CpSRK2E	pGADT7-T	self activated
CpSRK2A	CpPP2C65	-
CpSRK2A-like	CpPP2C65	-
CpACO1	CpPP2C65	-
CpACS1-like	CpPP2C65	-
CpACO-h1-like1	CpPP2C65	-
CpACO-h1-like2	CpPP2C65	self activated
CpACO-h1-like2	pGADT7-T	self activated
CpACO-h4-like	CpPP2C65	-

Figure S15. Y2H was used to verify the interactions of CpCML15 (a), CpPP2C46 (b) and CpPP2C65 (c) with proteins in ethylene synthesis and ABA signal transduction pathway. SD medium for yeast growth was lacking Trp, His, Leu, and Ade. Blue plaques display the interaction of protein staining with X-α-gal.

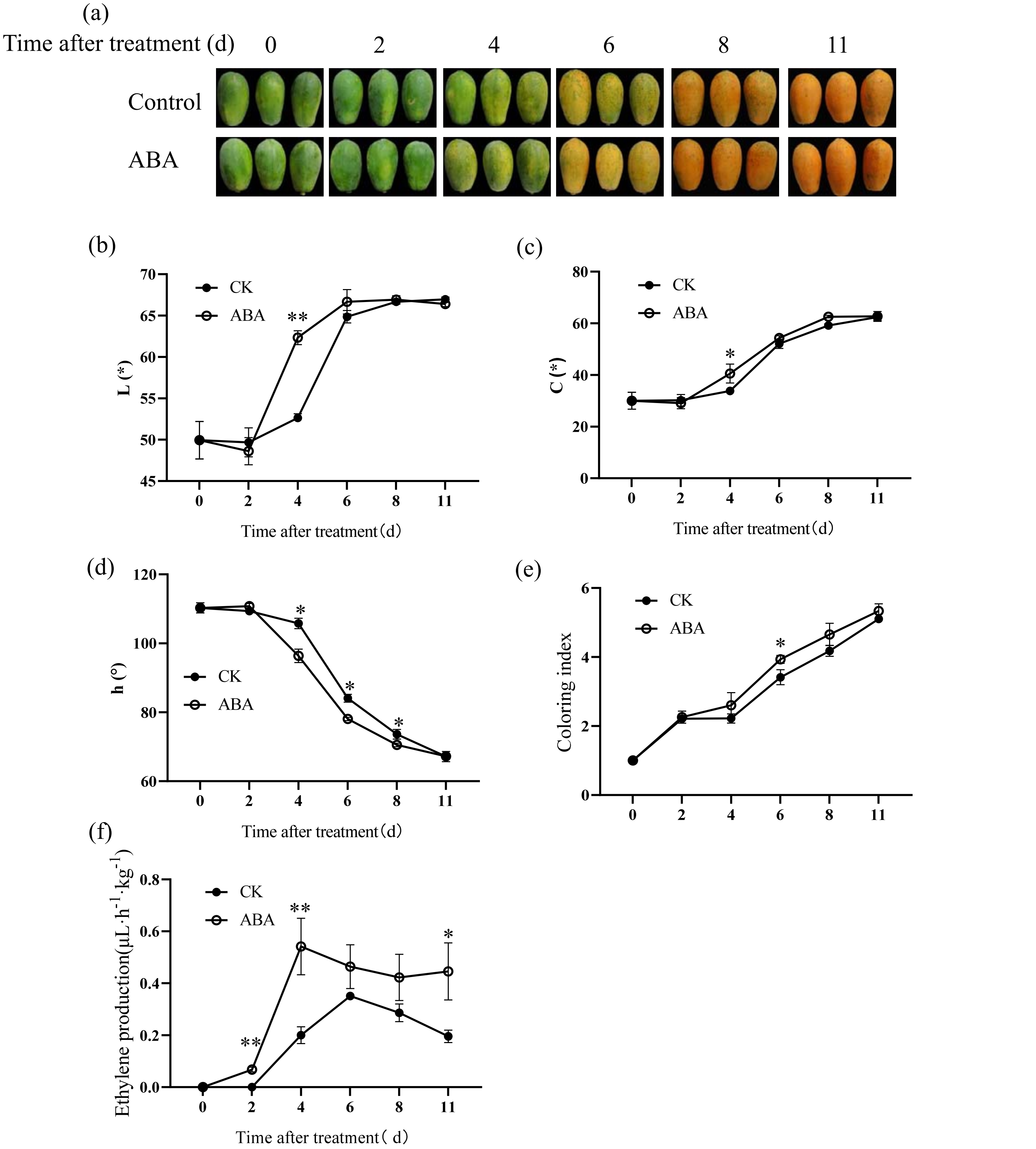

Figure S16. The ABA treatment accelerated the fruit-ripening process.

(a). Photographs of papaya fruit treated with or without ABA during the storage; (b-e). fruit coloring change during ripening process, including L value (b), C value (c), h value (d) and coloring index (e). (f) Fruit ethylene production treatment during the storage. Data are presented as the mean ± S.E. (n=3).The different letters indicate statistical differences between treatments at the 5% level.

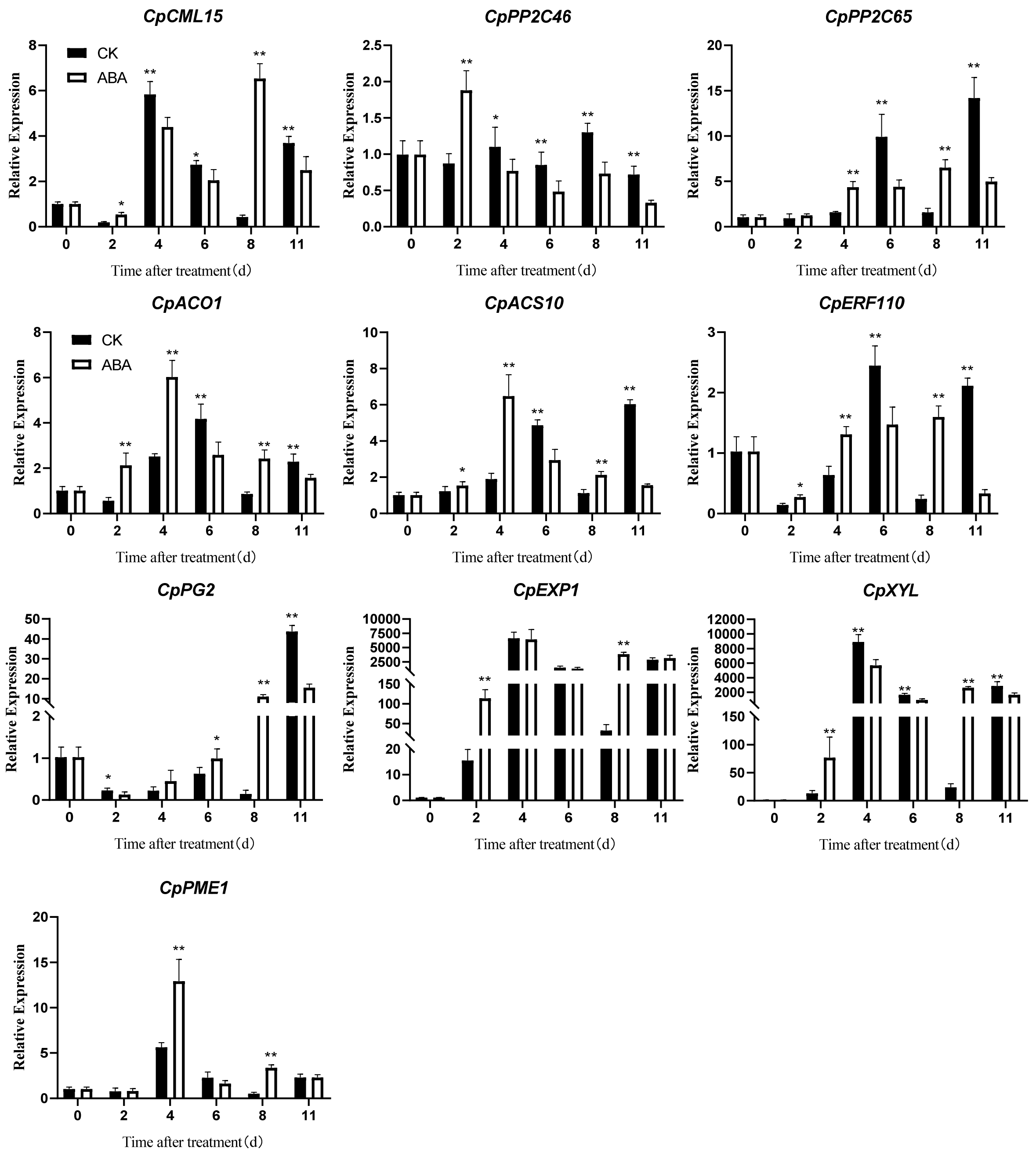

Figure S17. Transcripts of *CpCML15* and *CpPP2C46/65* and other genes involved in ethylene signal and fruit softening in fruit after ABA treatment were determined by RT-qPCR. Expression data at different sampling days are relative to 0 d (untreated fruits), which was set as 1. The CpTBP1 and CpTBP2 were used as reference genes. Data are presented as the means ±SD of three biological replicates. The different letters between treatments indicate statistical differences at the 5% level.

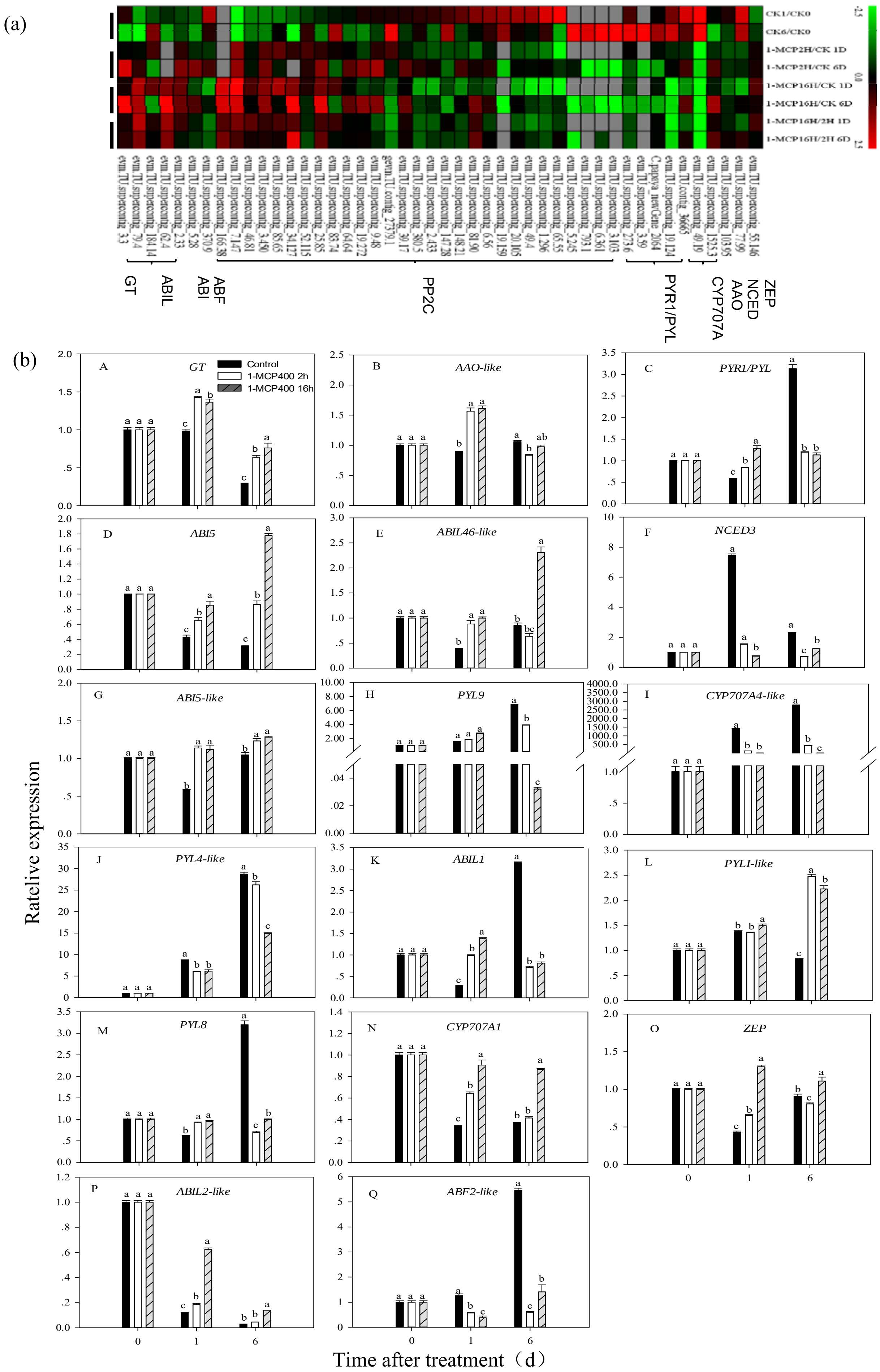

Figure S18. The expression profiles of differential expression genes in ABA pathways under different 1-MCP treatments. (a) Heatmap of the DEGs involved in ABA signal pathways. The data of the heatmap was extracted from our previous RNA-seq data (Zhu et al., 2019). (b) Expression profile validation by RT-qPCR. Expression data at different sampling days are relative to 0 d (untreated fruits), which was set as 1. The *CpTBP1* and *CpTBP2* were used as reference genes. Data are presented as means ±SD of three biological replicates. The different letters between treatments indicate statistical differences at the 5% level.

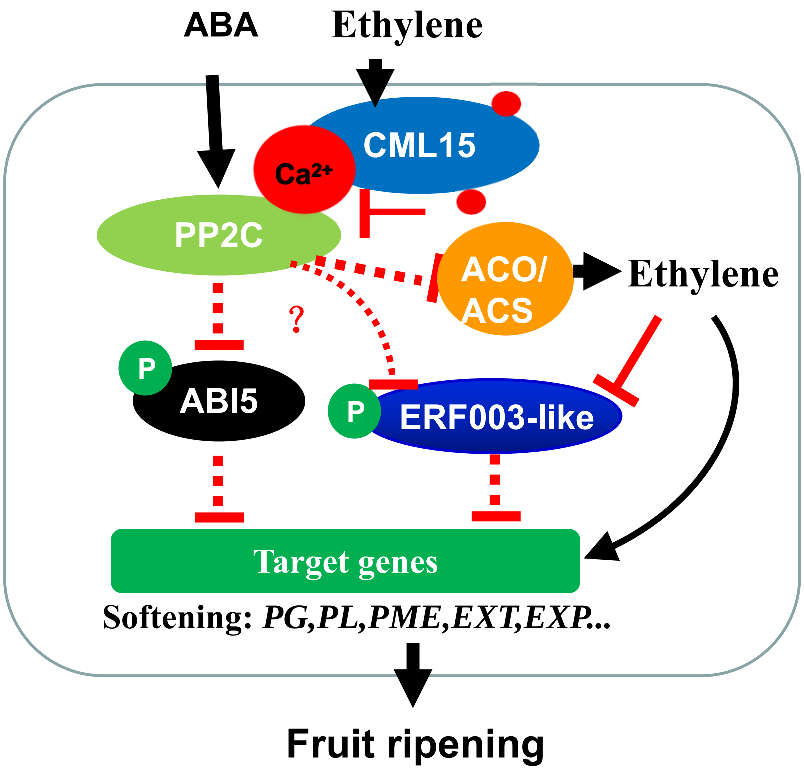

Figure S19. A proposed model of CML15-PP2Cs-ABI5/ERF003-like on papaya fruit ripening. ABA promoted papaya fruit ripening via the ABA signaling pathway. Ethylene accelerated fruit ripening and induced the expression of *CML15* and *PP2C46/65*. CML15 interacted with PP2Cs and regulated PP2C activity, thereby affecting PP2C phosphorylation and modification of function of transcriptional factor ABI5 and ERF003-like, then regulating the expression of downstream fruit ripening-related genes, and regulating fruit ripening.

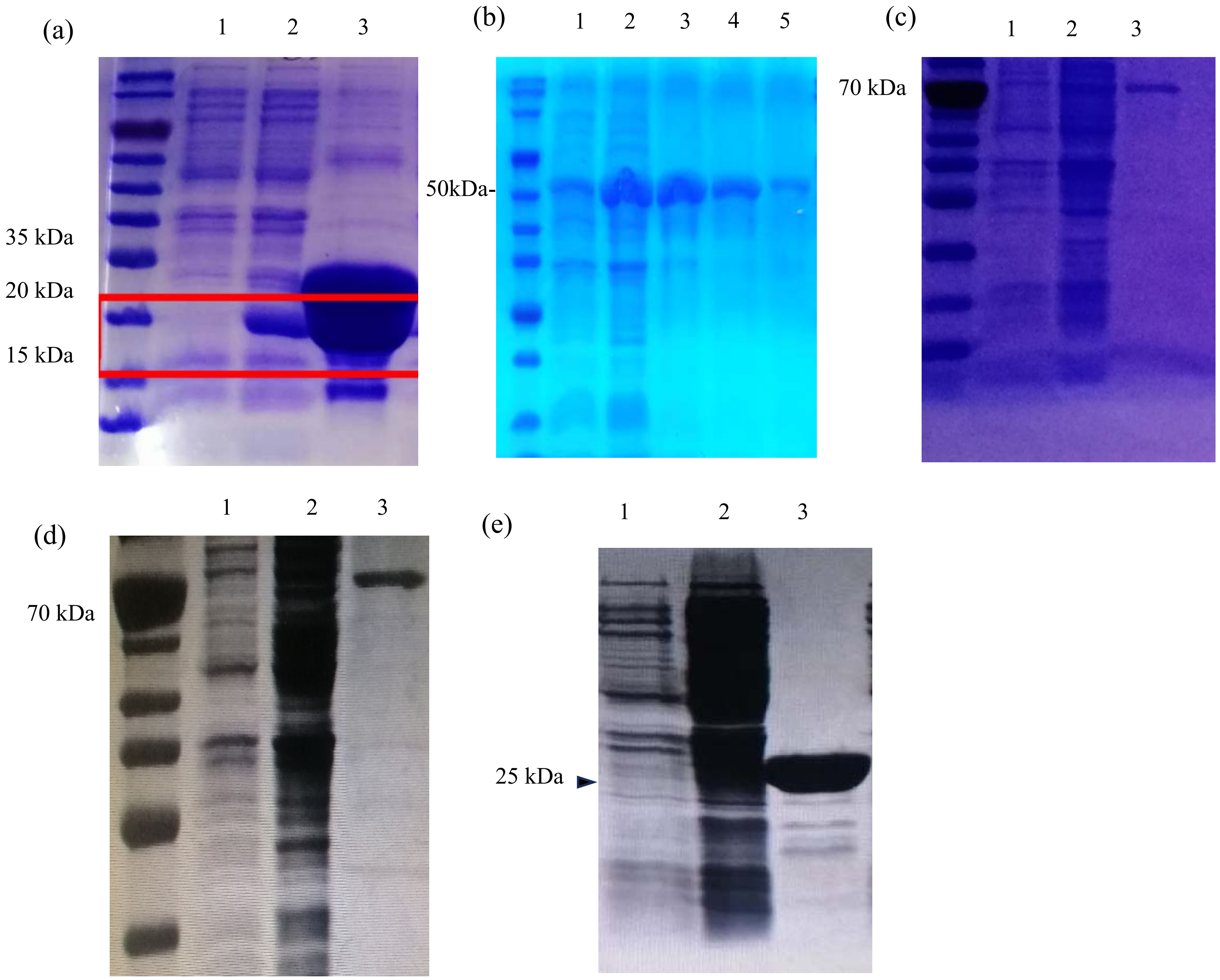

Figure S20 The recombinant proteins of CpCML15-His (a), CpCML15-MBP(b), CpPP2C46-GST (c), CpPP2C65-GST(d) and GST(e).

SDS–PAGE gel stained with coomassie blue demonstrating affinity purification of the recombinant proteins. Lane 1: non-induced protein; lane2: induced protein; lane 3(4,5): after purification of the induced protein.
